# Pharmacotherapy, acupoint stimulation, and psychotherapy for perimenopausal women with anxiety, depression, and panic disorder: a systematic review and network meta-analysis of randomized controlled trials

**DOI:** 10.3389/fpsyt.2026.1845876

**Published:** 2026-07-17

**Authors:** Jiamei Zhuang, Yan Zhou, Guangbin Yu, Honghui Cheng, Xiong Chen, Rui Qian

**Affiliations:** 1 The Seventh Clinical College of Guangzhou University of Chinese Medicine, Shenzhen Bao’an Chinese Medicine Hospital, Guangzhou University of Chinese Medicine, Shenzhen, Guangdong, China; 2 Clinical Medical College of Acupuncture Moxibustion and Rehabilitation, Guangzhou University of Chinese Medicine, Guangzhou, Guangdong, China

**Keywords:** acupoint stimulation, anxiety, depression, panic disorder, perimenopause, pharmacotherapy, psychotherapy

## Abstract

**Background:**

Perimenopausal women frequently experience physiological and psychological symptoms, including anxiety, depression, and panic disorders, mainly due to declining ovarian function and hormonal changes. Current options include pharmacotherapy, acupoint stimulation (AcuStim), and psychotherapy (psych), but their comparative efficacy and safety remain controversial.

**Objective:**

This network meta-analysis (NMA) systematically compared pharmacotherapy, AcuStim, and psychotherapy for perimenopausal anxiety, depression, and panic disorder, assessing clinical efficacy, adverse events (AEs), and changes in the Hamilton Depression Rating Scale (HAMD), Hamilton Anxiety Rating Scale (HAMA), Kupperman Index (KI), Self-rating Depression Scale (SDS), Self-rating Anxiety Scale (SAS), Pittsburgh Sleep Quality Index (PSQI), and serum hormone levels.

**Methods:**

We searched PubMed, Embase, Cochrane Library, Web of Science, CNKI, Wanfang, VIP, and SinoMed from inception to June 14, 2026, for randomized controlled trials (RCTs). A Bayesian NMA was performed, and the Surface Under the Cumulative Ranking Curve (SUCRA) was calculated.

**Results:**

The study included 131 RCTs, encompassing 11457 perimenopausal women diagnosed with emotional disorders. These trials evaluated three distinct treatment strategies. The NMA showed that the highest SUCRA probabilities were observed for drug_psych across HAMD (SUCRA = 92.4%), KI (SUCRA = 97.9%), SDS (SUCRA = 94.5%), PSQI (SUCRA = 98.1%), and follicle-stimulating hormone (FSH) (SUCRA = 96.1%) reduction and estradiol (E2) (SUCRA = 0.1%) elevation; for AcuStim_psych (SUCRA = 93.7%) in HAMA reduction; for psych (SUCRA = 98.9%) in SAS reduction; for drug_AcuStim in clinical efficacy (SUCRA = 9.0%) and luteinizing hormone (LH) reduction (SUCRA = 100%); and for control (SUCRA = 65.5%) in safety outcomes. In pharmacotherapy subgroup analyses, antidepressants (ADs)_Traditional Chinese medicine (TCM) ranked highest for HAMD (SUCRA = 87.2%) and safety (SUCRA = 82%), ADs_antipsychotics (AP) (SUCRA = 97.5%) for HAMA, and ADs_hormone replacement therapy (HRT) (SUCRA = 10.2%) for clinical efficacy.

**Conclusion:**

Pharmacological, acupoint stimulation, and psychological interventions each demonstrated therapeutic benefits for perimenopausal women with emotional disorders. Combination therapies generally showed more favorable efficacy across multiple psychological and endocrine outcomes than single-modality interventions, while no single treatment strategy was consistently superior across all outcomes. These findings may provide evidence to support individualized treatment selection according to patients’ clinical characteristics and therapeutic goals.

**Systematic review registration:**

https://www.crd.york.ac.uk/PROSPERO/, identifier CRD420261340530.

## 1 Introduction

The female perimenopausal period marks the transition from declining ovarian function to menopause, often accompanied by various physiological and psychological changes. In the late perimenopausal stage, gonadotropin (Gn) levels rise, along with increased plasma follicle-stimulating hormone (FSH) levels, indicating impaired ovarian function and decreased estradiol secretion. These changes can lead to symptoms such as hot flashes, night sweats, sleep disorders, and genitourinary discomfort (1). Additionally, they may affect the central nervous system, increasing the likelihood of emotional disorders like anxiety, depression, and panic disorder (2). Perimenopausal panic disorder is characterized by recurrent panic attacks (3). Research has linked emotional responses in animals facing different threat levels to human psychiatric conditions. Generalized anxiety disorder (GAD) and anticipatory anxiety are associated with primary defense mechanisms against potential threats, while proximal threats can induce panic-like behaviors and even panic disorder (4).

There are currently three primary intervention methods for treating mental disorders in perimenopausal women: pharmacotherapy, acupoint stimulation (AcuStim), and psychotherapy (psych). Pharmacotherapy is the most prevalent due to its rapid effectiveness. hormone replacement therapy (HRT) shows a clear advantage. For instance, Bazedoxifene combined with conjugated estrogen has been shown to markedly improve depressive symptoms and reduce scores on the Meno-D, a specific menopause depression rating scale (5). Large-sample clinical trials have demonstrated that desvenlafaxine, a serotonin-norepinephrine reuptake inhibitor (SNRI), significantly enhances the Hamilton Depression Rating Scale (HAMD) score in severe depression cases among perimenopausal women, effectively reducing symptoms of anxiety and depression (6). However, long-term HRT may increase the risk of breast cancer (7) and cardiovascular diseases (8). Traditional antidepressants, such as SNRIs and selective serotonin reuptake inhibitors (SSRIs), can also have persistent adverse effects(AEs), including osteoporosis, sexual dysfunction, and hyperprolactinemia (9). Acupoint stimulation has a favorable safety profile. It aids in treating insomnia by inhibiting sympathetic nerve activity and down-regulating the hypothalamic-pituitary-adrenal (HPA) axis (10). Improving sleep is an important preventive strategy against the development of mood disorders (11). Nonetheless, methodological limitations in acupuncture research are notable. Many trials in this field are constrained by small sample sizes and suboptimal methodological rigor. Double-blinding is particularly difficult to implement given the procedural nature of the intervention and ethical restrictions in China. Sham acupuncture, though frequently adopted as a control, remains a subject of debate: some authors contend that it may not be physiologically inert but rather elicit both non–specific and specific effects, thereby complicating the attribution of between–group differences (12). Psychological interventions are also effective. Cognitive Behavioral Therapy (CBT) significantly enhances emotions, behaviors, and interpersonal relationships by improving patients’ cognition. Substantial evidence supports its efficacy in relieving anxiety, depression, and sleep disorders (13). Mindfulness-Based Stress Reduction (MBSR) is a well-structured program that combines body scan exercises, sitting meditation, and gentle yoga postures, effectively alleviating psychological, physiological, and sexual symptoms in perimenopausal women (14). However, psychotherapy does not improve endocrine indicators in perimenopausal women, is less effective for severe mood disorders, and requires high patient compliance.

Most current randomized controlled trials (RCTs) adopt a head-to-head design, comparing a single treatment against control or another active comparator. Direct comparisons across pharmacotherapy, acupoint stimulation, and psychotherapy are scarce, and the comparative efficacy of combination strategies remains largely unexplored. Conventional pairwise meta-analyses cannot simultaneously evaluate all available interventions or combination regimens. For clinicians treating perimenopausal women with anxiety, depression, or panic disorder, the existing evidence offers limited support for selecting among treatment options. To overcome these limitations, we performed a network meta-analysis (NMA) integrating different intervention types, with subgroup analyses stratified by treatment category. We ranked interventions by surface under the cumulative ranking (SUCRA) probabilities for efficacy and safety, and provided league table estimates with 95% credible intervals (CrIs) alongside these rankings to furnish clinicians with a robust evidence base for treatment selection.

## 2 Materials and methods

This systematic review and NMA followed the Preferred Reporting Items for Systematic Reviews and Meta-Analyses of Network Meta-Analyses (PRISMA-NMA) guidelines. The complete checklist of items is available in **Supplementary Material 1**. The study protocol was prospectively registered on the PROSPERO platform (CRD420261340530).

### 2.1 Data sources and search strategies

We searched four English-language databases (PubMed, Embase, Cochrane, and Web of Science) and four Chinese-language databases (CNKI, Wanfang, VIP, and SinoMed) from the inception of each database to June 14, 2026. Our strategy included four components: population (including terms like “perimenopause”, “climacterium”, “anxiety disorder”, “anxiety”, “depressive disorder”, “panic disorder”), intervention (such as “psychotropic drugs”, “acupuncture therapy”, “psychotherapy”), and study type (including “Randomized Controlled Trial”, “randomized”, “placebo”). We limited our search to studies published in English and Chinese. Detailed information regarding the search strategy is provided in **Supplementary Material 2**.

### 2.2 Inclusion criteria

Included studies met the following criteria (1): Study type: RCTs (2). Study population: Women aged≥40 years with perimenopausal anxiety/depression/panic disorder, diagnosed per the Stages of Reproductive Aging Workshop (STRAW) (15), International Classification of Diseases (ICD-10) (16), Diagnostic and Statistical Manual of Mental Disorders (DSM-IV/V) (17, 18), or Chinese Classification and Diagnostic Criteria of Mental Disorders (CCMD-3) (19), or by validated scale cutoffs: HAMD≥8, Hamilton Anxiety Rating Scale (HAMA)≥14, Self-Rating Depression/Anxiety Scales (SDS/SAS)≥50, or Kupperman Index (KI)≥15 with concurrent mood elevation. Studies using only climacteric symptoms without mood thresholds were excluded (3). Intervention: Interventions comprised pharmacotherapy, Acupoint Stimulation, or psychotherapy, with combined treatment regimens indicated by an underscore. Combination therapy was included only when both intervention methods are explicitly implemented as independent, active treatment modalities. Definitions of combined interventions are detailed in **Table 1** (4). Outcomes: Studies were required to report at least one outcome measure. Primary outcomes included the Hamilton Depression Rating Scale (HAMD), the Hamilton Anxiety Rating Scale (HAMA) (with higher scores indicating greater symptom severity on both scales), clinical efficacy, and AEs. Clinical efficacy was defined as a ≥25% reduction in the HAMD score from baseline. AEs were defined as any adverse medical occurrence during the treatment period, regardless of causality. Secondary outcomes comprised the Kupperman Index (KI) for menopausal symptom severity, the Self-Rating Depression Scale (SDS) and Self-Rating Anxiety Scale (SAS) for mood assessment, the Pittsburgh Sleep Quality Index (PSQI) for sleep quality (with higher scores indicating poorer sleep), and serum hormone levels including FSH, luteinizing hormone (LH) and estradiol (E2).

**Table 1:** Definitions of intervention included in this study.

Interventions	Definitions
drug_AcuStim	A combined treatment using pharmacotherapy and Acupoint Stimulation
drug_psych	A combined treatment using pharmacotherapy and psychotherapy
AcuStim_psych	A combined treatment using Acupoint Stimulation and psychotherapy
ADs_TCM	A combined treatment using antidepressants and traditional chinese medicine
ADs_HRT	A combined treatment using antidepressants and hormone replacement therapy
ADs_AP	A combined treatment using antidepressants and antipsychotics
ADs_HRT_TCM	A combined treatment using antidepressants, hormone replacement therapy and traditional chinese medicine

### 2.3 Exclusion criteria

The following criteria were used for exclusion: (1) reviews and meta-analyses, (2) case reports, books, conferences, guidelines, letters, editorials, short surveys, notes, pre-published studies, and retracted studies, (3) studies with duplicate publications, (4) studies involving interventions or diseases not meeting the inclusion criteria, (5) studies for which full texts could not be retrieved, (6) studies on animal experiments, (7) non-English studies and non-Chinese studies, (8) studies still under registration, (9) studies lacking relevant outcomes and data, and (10) cohort studies.

### 2.4 Literature screening and data extraction

All records were imported into EndNote 20, and duplicates were removed using the software. Two researchers, Jiamei Zhuang and Yan Zhou, screened all titles and abstracts to identify potentially relevant studies for full-text review. Subsequently, two independent reviewers assessed these studies based on the inclusion and exclusion criteria. Only studies meeting the inclusion criteria underwent literature quality evaluation and data extraction for the subsequent NMA. The included studies were assigned numbers, and data were collected in an Excel sheet. This data included the author, publication year, country, study type, patient details, sample size, age, interventions, as well as the sample size and age of both intervention and control groups, duration, diagnosis and outcomes.

Two researchers conducted the literature screening and data extraction. If disagreements arose during this process, a third researcher was invited to discuss and resolve the issues.

### 2.5 Risk of bias assessment

Two researchers (Jiamei Zhuang and Yan Zhou) assessed the risk of bias in RCTs using Cochrane Risk of Bias Tool 2.0 (20). The evaluation criteria for each study encompassed randomization process, deviations from intended interventions, missing outcome data, measurement of the outcome and selection of the reported result. Each domain was rated as low, high, or unclear risk of bias and any discrepancies were resolved through consensus. If the risk of bias assessment results for all domains were “low risk,” then the overall risk of bias was “low risk”; If the risk of bias assessment result for any domain was “some risk” and there were no domains with “high risk,” then the overall risk of bias was “some risk”; If the risk of bias assessment for even one domain was rated as “high,” then the overall risk of bias was “high”.

### 2.6 Statistical analysis methods

In this study, we utilized Stata 17.0 and Rstudio 4.3.1 for all statistical analyses. Initially, we constructed a network structure diagram to visually display the direct and indirect comparison relationships among various intervention measures from the included studies. Additionally, we evaluated the network’s connectivity.

In the Bayesian framework, a random-effects model was used to perform a NMA. For binary outcome variables, the Relative Risk (RR) and its 95% credible interval (CrI) served as the effect size to assess the relative efficacy of different interventions. For continuous outcomes, the Standardized Mean Difference (SMD) was applied.

We stratified analyses into two dimensions: (a) overall networks comparing the three major intervention categories (pharmacotherapy, acupoint stimulation, psychotherapy) and their combinations, and (b) subgroup networks stratified by drug class, acupoint stimulation modality, and psychotherapeutic approach, to determine whether inconsistency arose from cross–category or within–class differences.

To assess the relative effectiveness of different interventions, we calculated the SUCRA values for each intervention and created a treatment ranking plot. A higher SUCRA value signifies superior performance of the intervention across various comparisons.

The evaluation of the network consistency hypothesis involves two primary tests: 1) the loop-specific method assesses local inconsistency by examining closed loops, and 2) the global inconsistency test, which is based on the design-by-treatment interaction model. A P-value < 0.05 suggests a significant inconsistency between direct and indirect evidence within the network, necessitating cautious interpretation of the relevant comparison results. For loop inconsistency, results were generated using Stata (**Supplementary Material 5**). For global inconsistency, the test was performed using R (**Supplementary Material 4**), and the corresponding forest plot was constructed using Stata (**Supplementary Material 6**).

The study constructed a league table to display the effect estimates and CrIs for each intervention compared to the reference control. To evaluate potential publication bias, researchers created a comparison-adjusted funnel plot and conducted a symmetry analysis.

In this study, results were considered statistically significant when the 95% CrI for the RR did not include 1 or the 95% CrI for the SMD did not include 0. Heterogeneity was quantified using the I² statistic, by conventional thresholds, I² ≤ 50% indicated low heterogeneity, whereas I² > 50% indicated substantial heterogeneity among studies. For comparisons with I² ≤ 50%, a fixed–effect model was applied; for those with I² > 50%, a random–effects model was used instead. To explore heterogeneity sources, subgroup analyses were performed stratified by control type (drug, SSRI, ADs_HRT, and control), intervention category, and outcome measure, using the corresponding model based on the observed I². Detailed subgroup results are provided in **Supplementary Material 8**, and heterogeneity results for all comparisons are shown in **Supplementary Material 4**.

## 3 Results

### 3.1 Literature retrieval

**Figure 1:**
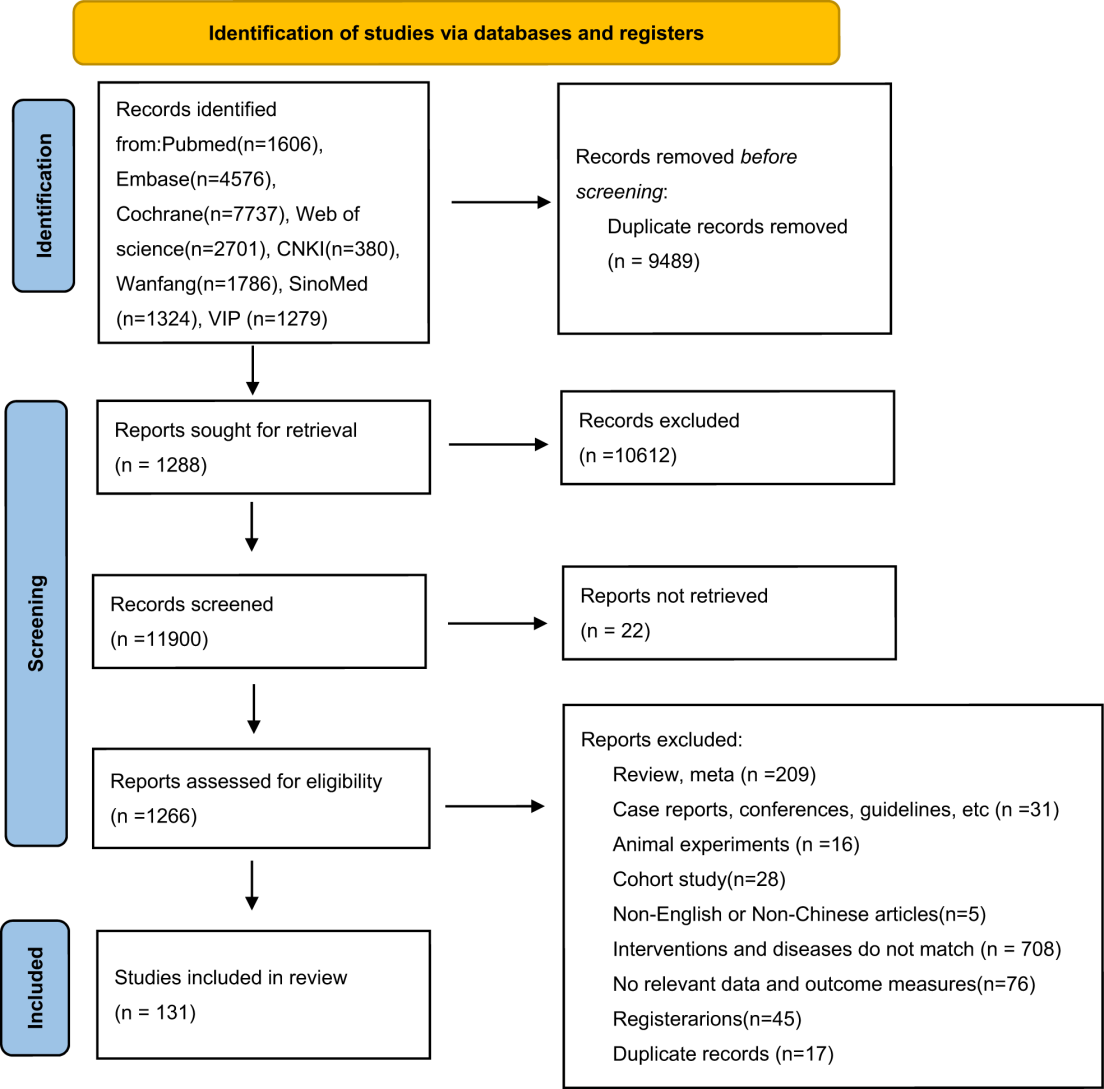
PRISMA flow diagram.

We identified 21389 articles from the database and selected 1266 for full-text screening. Of these, 1135 articles were excluded for the following reasons: reviews and meta-analyses (n = 209), case reports, conferences, guidelines, etc. (n = 31), animal experiments (n = 16), cohort studies (n = 28), non-English or non-Chinese articles (n = 5), non-matching interventions and diseases (n = 708), absence of relevant data and outcomes (n = 76), duplication (n = 17), and registered studies (n = 45). Ultimately, 131 articles (5, 6, 21–149) met the inclusion criteria. **Figure 1** illustrates the process of study retrieval, screening, and selection. All included studies were RCTs.

### 3.2 Basic characteristics of the included studies

Among the 131 included studies, all were RCTs. Specifically, 114 studies took place in the China, 9 in USA, 3 in Australia, 2 in Iran, and 1 each in the Republic of Türkiye, Mexico and Japan. These studies were published between 1999 and 2026. A total of 11457 participants aged≥40 years participated in these studies, with follow-up periods ranging from 1 to 12 months. The definitions of perimenopausal anxiety, depression, and panic disorder were homogeneous across the trials. The interventions in the studies were categorized into three main types: pharmacotherapy, Acupoint Stimulation, and psychotherapy. Pharmacotherapy was examined in 148 studies, with the most frequently investigated agents being fluoxetine (20 studies), estrogen (13 studies), Traditional Chinese Medicine (TCM) (14 studies), delexin and citalopram (11 each), fluoxetine_estrogen and paroxetine (9 each), tibolone and sertraline (5 each), amitriptyline (4 studies), and Sequential estrogen-progestogen therapy (SEPT), escitalopram, and duloxetine (3 each). Two studies each focused on fluoxetine_estrogen_TCM, citalopram_TCM, desvenlafaxine, citalopram_quetiapine, amitriptyline_SEPT, paroxetine_tibolone, paroxetine_TCM, fluoxetine_tibolone, and alprazolam. The remaining agents were each examined in a single study, including zolpidem, paroxetine_olanzapinee, paroxetine_estradiol, oryzanol, venlafaxine, sertraline_progesterone, SEPT_alprazolam, agomelatine, fluvoxamine maleate, fluoxetine_TCM, fluoxetine_progesterone, estrogen_anti-anxiety medications, estazolam, delexin_TCM, clomipramine, citalopram_tibolone, buspirone_TCM, bazedoxifene_estrogen, livmin, and Premarin_Medroxyprogesterone. Psychotherapy was the focus of 8 studies, including 3 on general psychotherapy (unspecified type), and 1 each on cognitive behavioral therapy (CBT), emotional freedom techniques (EFT), behavioral weight loss, behavioral weight loss_CBT, and music therapy. Acupoint Stimulation was investigated in 43 studies, with 28 on acupuncture, 7 on electroacupuncture, 3 on Acupoint catgut embedding (ACE). Single studies examined scalp acupuncture, heat-sensitive moxibustion, ear acupuncture, auricular acupressure, and acupuncture_Wheat grain moxibustion. A total of 52 studies explored combination therapies. The most common combination was TCM_acupuncture (17 studies), followed by TCM_psychotherapy, sertraline_acupuncture, TCM_electroacupuncture, and delexin_TCM_acupuncture (3 studies each), and paroxetine_electroacupuncture, delexin_acupuncture, and pharmacotherapy_psychotherapy (2 studies each). The remaining combinations were each reported in a single study: MusicTherapy_ Auricular Point Seed Burying (APS), livmin_acupuncture, fluvoxamine maleate_CBT, fluoxetine_psychotherapy, fluoxetine_estradiol_psychotherapy, estrogen_acupuncture, escitalopram_CBT, duloxetine_group psychotherapy, citalopram_electroacupuncture, citalopram_Musictherapy_Antipsychotics (AP), amitriptyline_ electroacupuncture, acupuncture_psychotherapy, acupuncture_group psychotherapy, fluoxetine_estradiol_TCM_psychotherapy, paroxetine_estradiol_electroacupuncture, pharmacotherapy_CBT, and citalopram_tibolone_electroacupuncture. Additionally, control groups included 19 standard control groups. Among the outcome indicators, 89 studies reported on HAMD, 35 on HAMA, 68 on clinical efficacy, 34 on AE, 34 on KI, 17 on SDS, 11 on SAS, 17 on PSQI, 37 on E2, 33 on FSH, 30 on LH. The basic characteristics of the included studies are detailed in **Supplementary Material 3**.

### 3.3 Assessment of risk of bias

**Figure 2:**
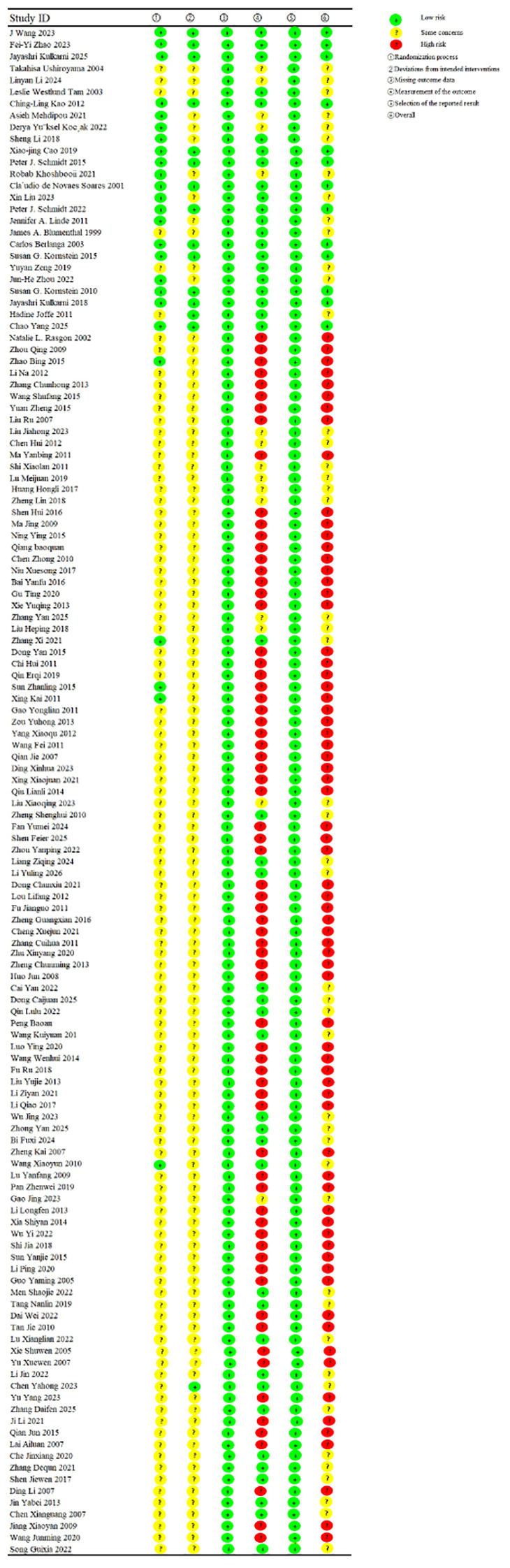
Risk of Bias graph.

In RCTs, risk of bias was assessed using the RoB 2.0 tool across five domains. In the Randomization process domain, 25 studies were rated as low risk, whereas 106 were rated as unclear. In the Deviations from intended interventions domain, 15 studies were rated as low risk and 116 as unclear. All studies were rated as low risk for Missing outcome data. For Measurement of the outcome, 15 studies were rated as unclear, 45 as low risk, and 71 as high risk. All studies were rated as low risk for Selection of the reported result. Overall, 47 studies were judged as having some concerns, 13 as low risk, and 71 as high risk. **Figure 2** provide detailed information on the bias risk of all included studies.

### 3.4 Network meta-analysis

#### 3.4.1 Hamilton depression rating scale

##### 3.4.1.1 Three major intervention categories

**Figure 3:**
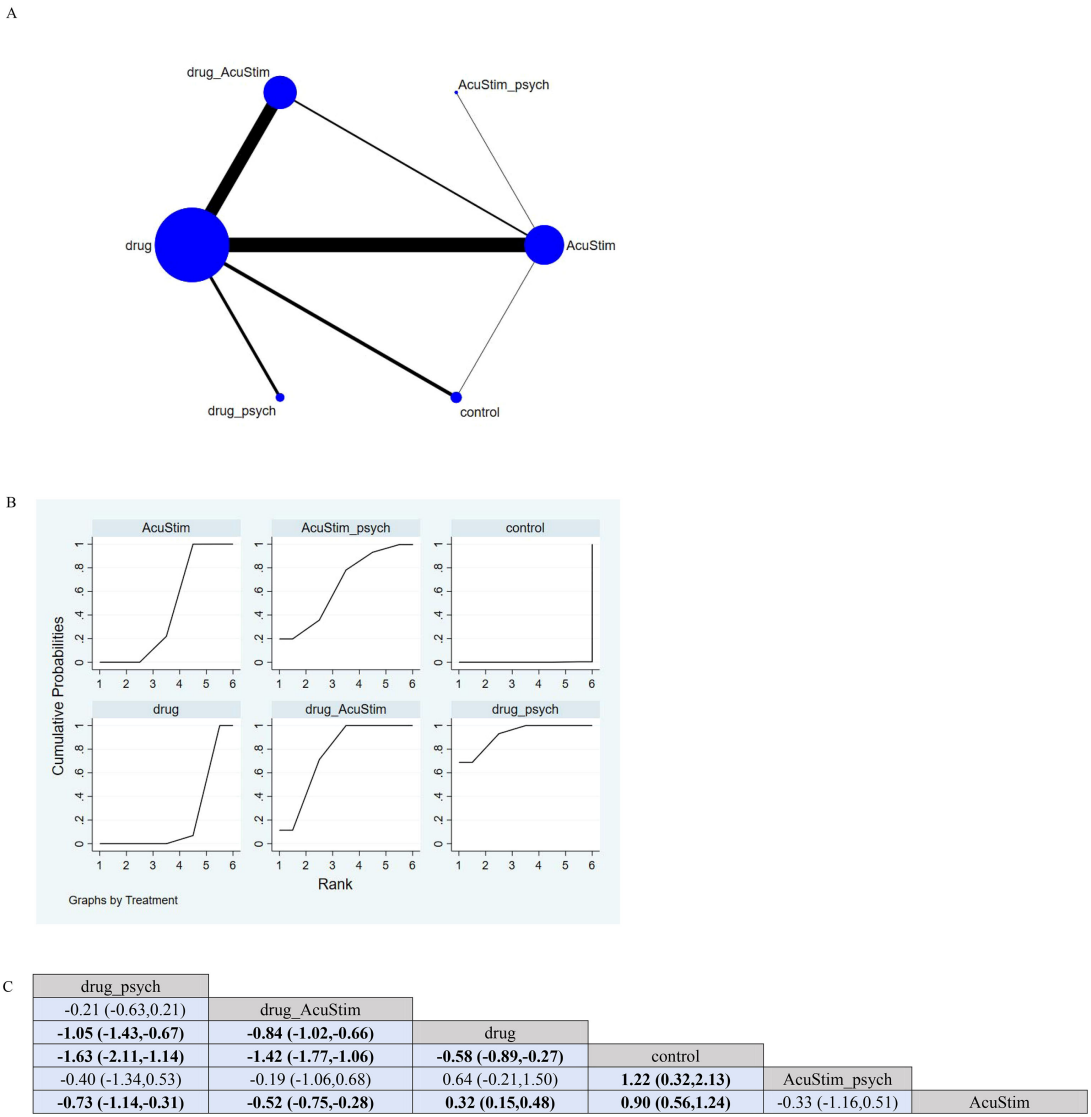
Network meta-analysis results for the HAMD outcome across the three major intervention categories (drug, Acupoint Stimulation, and psychotherapy). **(A)** Network plot of comparisons. **(B)** SUCRA ranking plot of all interventions. **(C)** League table of pairwise comparisons with SMDs and 95% CrIs.

A total of 89 studies reported HAMD. Among them, 55 evaluated the effects of three major intervention categories. The number of intervention arms was 53 for drug, 26 for AcuStim, 21 for drug_AcuStim, 7 for control, 5 for drug_psych, and 1 for AcuStim_psych. The most frequently studied nodes were drug, followed by AcuStim and drug_AcuStim. The most frequent direct comparisons were between drug and drug_AcuStim, drug and AcuStim. In total, 7 pairs of direct relationships were identified among the various interventions. A closed-loop, indicating an indirect relationship, was formed by drug, control, AcuStim and drug_AcuStim. The specific network diagram is depicted in **Figure 3A**.

Based on the SUCRA values, drug_psych (SUCRA = 92.4%) demonstrated the most significant reduction in HAMD among the 6 interventions. It was followed by drug_AcuStim (SUCRA = 76.5%) and AcuStim_psych (SUCRA = 65.2%). **Figure 3B** illustrates the specific overall intervention diagram.

From the league table, compared with control, all active interventions demonstrated significantly greater reductions in HAMD scores: drug_psych (SMD = -1.63, 95% CrI: -2.11 to -1.14), drug_AcuStim (SMD = -1.42, 95% CrI: -1.77 to -1.06), AcuStim (SMD = 0.90, 95% CrI: 0.56 to 1.24), AcuStim_psych (SMD = 1.22, 95% CrI: 0.32 to 2.13), and drug (SMD = -0.58, 95% CrI: -0.89 to -0.27). Notably, the two combination therapies, drug_psych and drug_AcuStim, each surpassed drug and AcuStim in efficacy: drug_psych was superior to drug (SMD = -1.05, 95% CrI: -1.43 to -0.67) and AcuStim (SMD = -0.73, 95% CrI: -1.14 to -0.31), while drug_AcuStim also outperformed drug (SMD = -0.84, 95% CrI: -1.02 to -0.66) and AcuStim (SMD = -0.52, 95% CrI: -0.75 to -0.28). Furthermore, AcuStim showed greater efficacy than drug alone (SMD = 0.32, 95% CrI: 0.15 to 0.48). The comparative results are detailed in **Figure 3C**.

The global inconsistency test indicated no significant inconsistency across the network (P = 0.053, I² = 0.3%). Two closed loops were identified: AcuStim-control-drug and AcuStim-drug-drug_AcuStim. The 95% CrI for the latter included 0, suggesting no significant loop inconsistency. The global inconsistency forest plot and the HAMD forest plot are presented in **Supplementary Materials 6**, **7**, respectively (**Supplementary Figures 1.1, S2.1**).

##### 3.4.1.2 Pharmacotherapies

**Figure 4:**
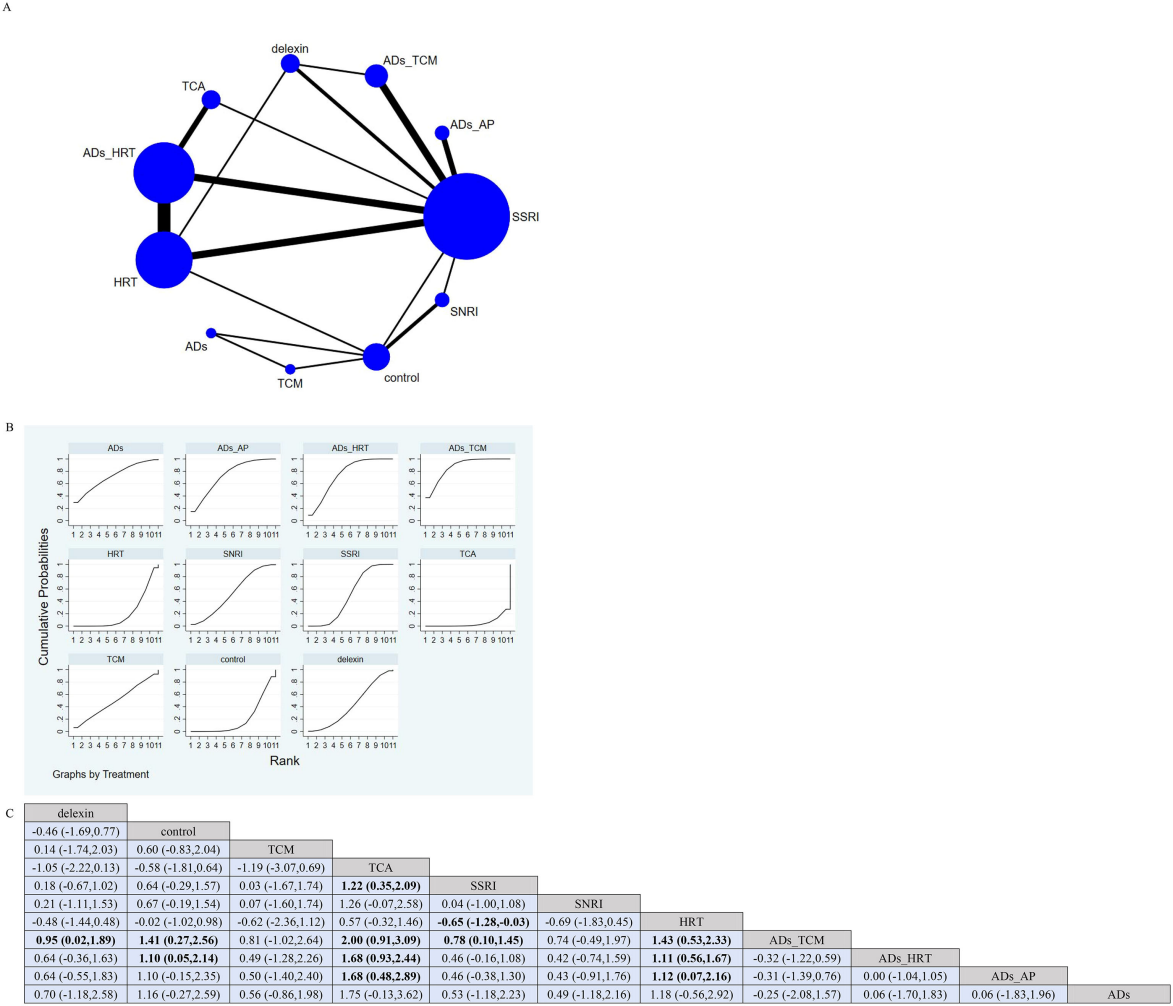
Network meta-analysis results for the HAMD outcome within pharmacotherapy subgroups. **(A)** Network plot of comparisons. **(B)** SUCRA ranking plot of all interventions. **(C)** League table of pairwise comparisons with SMDs and 95% CrIs.

A total of 89 studies reported HAMD. Of these, 28 assessed the pharmacotherapies in this subgroup analysis. The intervention arms numbered 16 for SSRI, 11 for Antidepressants (ADs)_HRT, 9 for HRT, 5 for ADs_TCM, 5 for control, 4 for Tricyclic Antidepressants (TCA), 3 for SNRI, 3 for ADs_AP, 3 for delexin, 1 for TCM, and 1 for ADs. The most commonly examined interventions were SSRI, followed by ADs_HRT, and HRT. The most prevalent pairwise comparisons involved HRT versus ADs_HRT, followed by SSRI versus HRT, SSRI versus ADs_HRT. A total of 17 direct comparisons were identified within the network. four closed loops involving HRT-ADs_HRT-TCA-SSRI, control-SSRI-HRT-delexin-ADs_TCM, ADs-TCM-control, control-SNRI-SSRI provided indirect evidence among these interventions. The network structure is presented in **Figure 4A**.

According to the SUCRA rankings, ADs_TCM (SUCRA = 87.2%) ranked first in reducing HAMD among the 11 included interventions. It was followed by ADs_HRT (SUCRA = 74.7%) and ADs_AP (SUCRA = 73.8%). The ranking plot is shown in **Figure 4B**.

Compared with TCA, several treatments demonstrated significantly greater efficacy in reducing HAMD scores. Specifically, ADs_TCM (SMD = 2.00, 95% CrI: 0.91 to 3.09), ADs_HRT (SMD = 1.68, 95% CrI: 0.93 to 2.44), ADs_AP (SMD = 1.68, 95% CrI: 0.48 to 2.89), and SSRI (SMD = 1.22, 95% CrI: 0.35 to 2.09) all showed significant improvements over TCA. ADs_TCM also demonstrated superior efficacy compared with control (SMD = 1.41, 95% CrI: 0.27 to 2.56), SSRI (SMD = 0.78, 95% CrI: 0.10 to 1.45), HRT (SMD = 1.43, 95% CrI: 0.53 to 2.33), and delexin (SMD = 0.95, 95% CrI: 0.02 to 1.89). ADs_HRT was more effective than control (SMD = 1.10, 95% CrI: 0.05 to 2.14) and HRT (SMD = 1.11, 95% CrI: 0.56 to 1.67). Notably, SSRI outperformed HRT with an SMD of -0.65 (95% CrI: -1.28 to -0.03). Additionally, ADs_AP showed a significant advantage over HRT (SMD = 1.12, 95% CrI: 0.07 to 2.16). The league table is shown in **Figure 4C**.

Global inconsistency was not significant across the pharmacotherapy network, as indicated by the inconsistency test (P = 0.161, I² = 2%). A total of six closed loops were identified: ADs_HRT–SSRI–TCA, HRT–SSRI–control, ADs_HRT–HRT–SSRI, HRT–SSRI–delexin, SNRI–SSRI–control, and ADs_TCM–SSRI–delexin. The 95% CrIs for all identified loops contained 0, suggesting that loop inconsistency was not statistically significant. Detailed results are presented in the supplementary figures: global inconsistency forest plot (**Supplementary Material 6**; **Supplementary Figure 1.2**), and forest plot (**Supplementary Material 7**; **Supplementary Figure 2.2**).

#### 3.4.2 Hamilton anxiety rating scale

##### 3.4.2.1 Three major intervention categories

**Figure 5:**
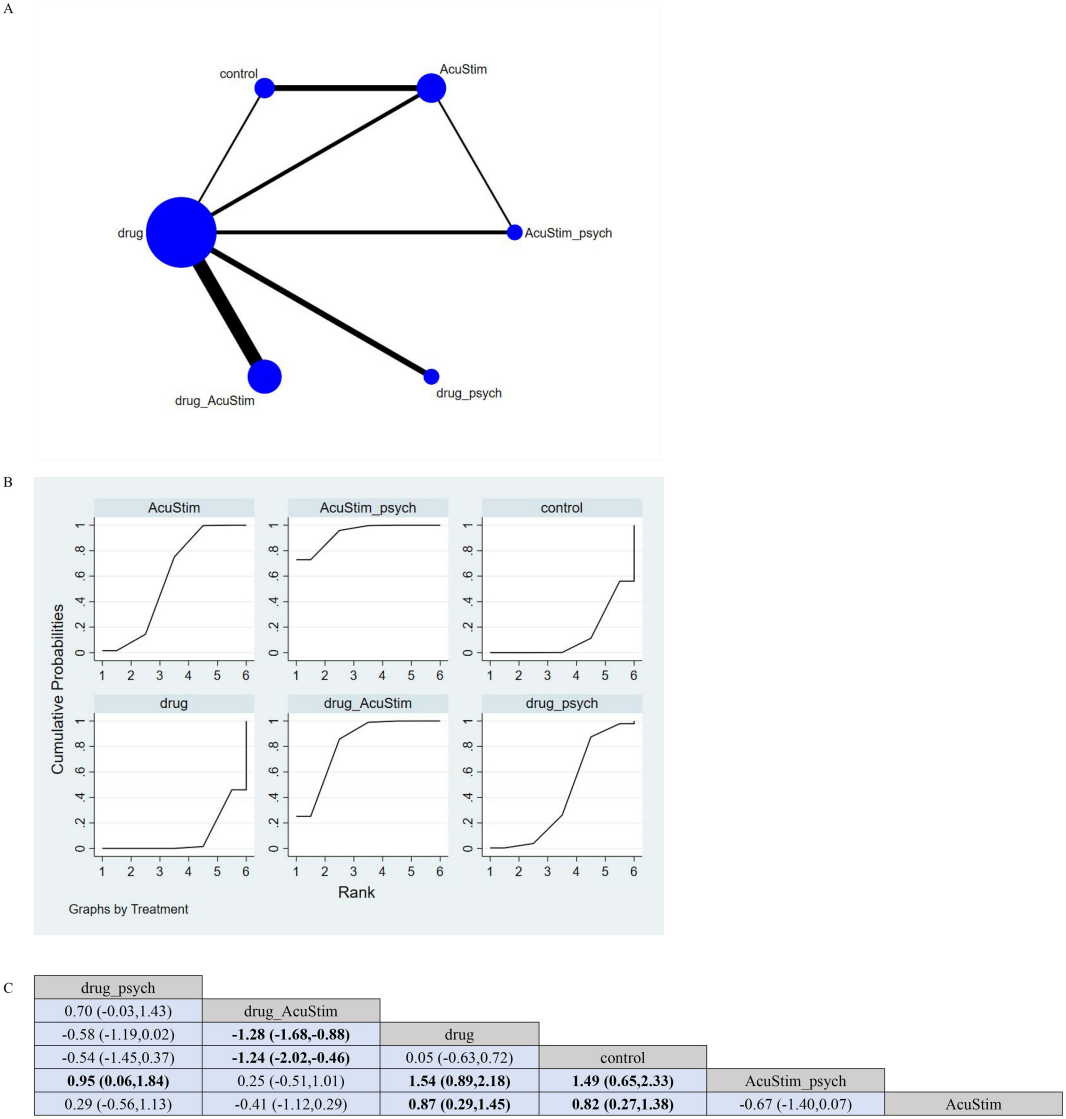
Network meta-analysis results for the HAMA outcome across the three major intervention categories (drug, Acupoint Stimulation, and psychotherapy). **(A)** Network plot of comparisons. **(B)** SUCRA ranking plot of all interventions. **(C)** League table of pairwise comparisons with SMDs and 95% CrIs.

Of the 35 studies that reported HAMA, 19 examined the three major intervention categories (drug, Acupoint Stimulation, and psychotherapy). The distribution of intervention arms was 15 for drug, 7 for drug_AcuStim, 6 for AcuStim, 4 for control, 3 for AcuStim_psych, and 3 for drug_psych. The nodes with the highest frequency were drug, followed by drug_AcuStim and AcuStim. The most frequent comparisons were drug versus drug_AcuStim. The network consisted of 7 direct pairwise comparisons. An indirect evidence loop was formed by drug, AcuStim_psych, AcuStim, and control. The network diagram is illustrated in **Figure 5A**.

Based on the SUCRA values, AcuStim_psych (SUCRA = 93.7%) achieved the most significant reduction in HAMA scores among the 6 interventions. It was followed closely by drug_AcuStim (SUCRA = 82%) and AcuStim (SUCRA = 58.2%). The detailed overall intervention graph is presented in **Figure 5B**.

From the league table, when compared with AcuStim_psych, drug (SMD = 1.54, 95% CrI: 0.89 to 2.18), control (SMD = 1.49, 95% CrI: 0.65 to 2.33), and drug_psych (SMD = 0.95, 95% CrI: 0.06 to 1.84) all showed significantly less efficacy in reducing HAMA scores. Compared with AcuStim, drug (SMD = 0.87, 95% CrI: 0.29 to 1.45) and control (SMD = 0.82, 95% CrI: 0.27 to 1.38) were also significantly less effective. In contrast, drug_AcuStim demonstrated significantly superior efficacy over drug (SMD = -1.28, 95% CrI: -1.68 to -0.88) and control (SMD = -1.24, 95% CrI: -2.02 to -0.46). **Figure 5C** presents the detailed results.

Global inconsistency across the network was not significant (P = 0.343, I² = 4%). Two closed loops were identified: AcuStim–control–drug and AcuStim–AcuStim_psych–drug, both with 95% CrIs containing 0, indicating no significant loop inconsistency. The global inconsistency forest plot and the HAMA forest plot are available in **Supplementary Figure 1.3** (**Supplementary Material 6**), **Supplementary Figure 2.3** (**Supplementary Material 7**).

##### 3.4.2.2 Pharmacotherapies

**Figure 6:**
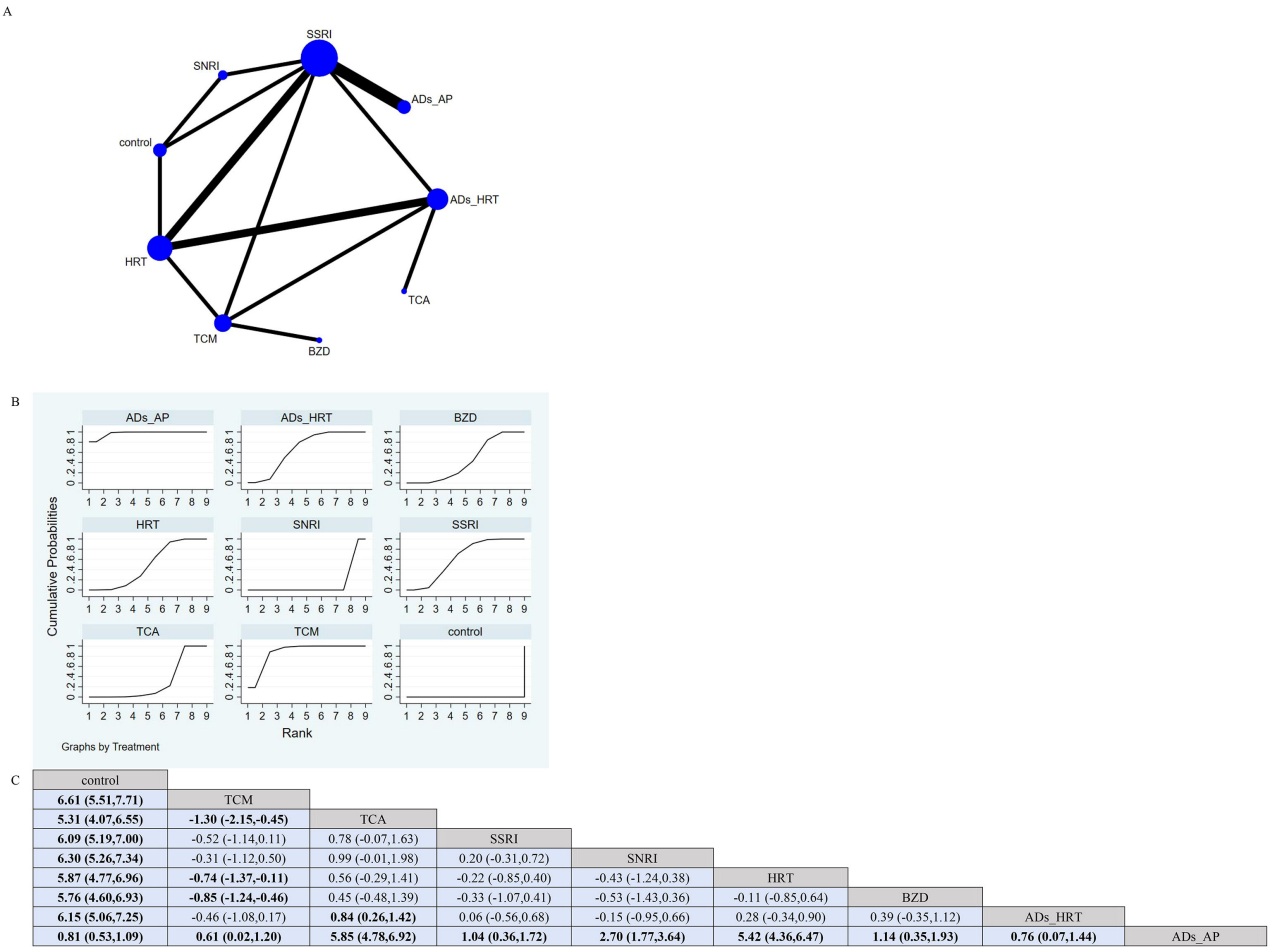
Network meta-analysis results for the HAMA outcome within pharmacotherapy subgroups. **(A)** Network plot of comparisons. **(B)** SUCRA ranking plot of all interventions. **(C)** League table of pairwise comparisons with SMDs and 95% CrIs.

In the 35 studies reporting HAMA, 10 studies investigated various pharmacotherapies within the drug subgroups. The number of arms was 6 for SSRI, 3 for ADs_HRT, 3 for HRT, 3 for ADs_AP, 2 for SNRI, 2 for control, 2 for TCM, 1 for TCA, and 1 for benzodiazepines (BZD). The most frequently evaluated nodes were SSRI, with ADs_HRT and HRT ranking second and third. The most common direct comparisons were between SSRI and ADs_AP, followed by SSRI and HRT, HRT and ADs_HRT. Overall, 13 direct relationships were observed among the interventions. A closed loop was identified, with SSRI, SNRI, control, HRT, TCM and ADs_HRT, forming an indirect comparison pathway. A visual summary of the network structure is provided in **Figure 6A**.

The SUCRA analysis revealed that ADs_AP (SUCRA = 97.5%) was the most effective intervention for HAMA among the 9 treatments within the drug subgroups. TCM (SUCRA = 88.1%) and ADs_HRT (SUCRA = 66.5%) ranked second and third, respectively. **Figure 6B** presents the overall ranking diagram.

From the league table, when compared with control, all other interventions demonstrated significantly better HAMA reduction: TCM (SMD = 6.61, 95% CrI: 5.51 to 7.71), SSRI (SMD = 6.09, 95% CrI: 5.19 to 7.00), SNRI (SMD = 6.30, 95% CrI: 5.26 to 7.34), HRT (SMD = 5.87, 95% CrI: 4.77 to 6.96), BZD (SMD = 5.76, 95% CrI: 4.60 to 6.93), TCA (SMD = 5.31, 95% CrI: 4.07 to 6.55), ADs_HRT (SMD = 6.15, 95% CrI: 5.06 to 7.25), and ADs_AP (SMD = 0.81, 95% CrI: 0.53 to 1.09). ADs_AP was associated with significantly superior efficacy compared with TCA (SMD = 5.85, 95% CrI: 4.78 to 6.92), SNRI (SMD = 2.70, 95% CrI: 1.77 to 3.64), BZD (SMD = 1.14, 95% CrI: 0.35 to 1.93), SSRI (SMD = 1.04, 95% CrI: 0.36 to 1.72), ADs_HRT (SMD = 0.76, 95% CrI: 0.07 to 1.44), TCM (SMD = 0.61, 95% CrI: 0.02 to 1.20), and HRT (SMD = 5.42, 95% CrI: 4.36 to 6.47). TCM also showed significant advantages over TCA (SMD = -1.30, 95% CrI: -2.15 to -0.45), HRT (SMD = -0.74, 95% CrI: -1.37 to -0.11), and BZD (SMD = -0.85, 95% CrI: -1.24 to -0.46). ADs_HRT was significantly more effective than TCA (SMD = 0.84, 95% CrI: 0.26 to 1.42). The detailed results is depicted in **Figure 6C**.

The global inconsistency test revealed significant inconsistency across the pharmacotherapy network (P < 0.001, I² = 5%). A total of five closed loops were identified: SNRI–SSRI–control, HRT–SSRI–TCM, HRT–SSRI–control, ADs_HRT–HRT–SSRI, and ADs_HRT–HRT–TCM. The 95% CrIs for all identified loops excluded 0, suggesting significant loop inconsistency. To explore the source of this inconsistency, subgroup analyses stratified by pharmacotherapy type were performed with SSRI as the reference. The results showed no significant difference between subgroups (p = 0.158), indicating that pharmacotherapy classification did not explain the observed inconsistency. (**Supplementary Material 8**, **Supplementary Figure 3.1**). The global inconsistency forest plot and the HAMA forest plot are presented in **Supplementary Materials 6**, **7**, respectively (**Supplementary Figures 1.4, S2.4**).

#### 3.4.3 Clinical efficacy

##### 3.4.3.1 Three major intervention categories

**Figure 7:**
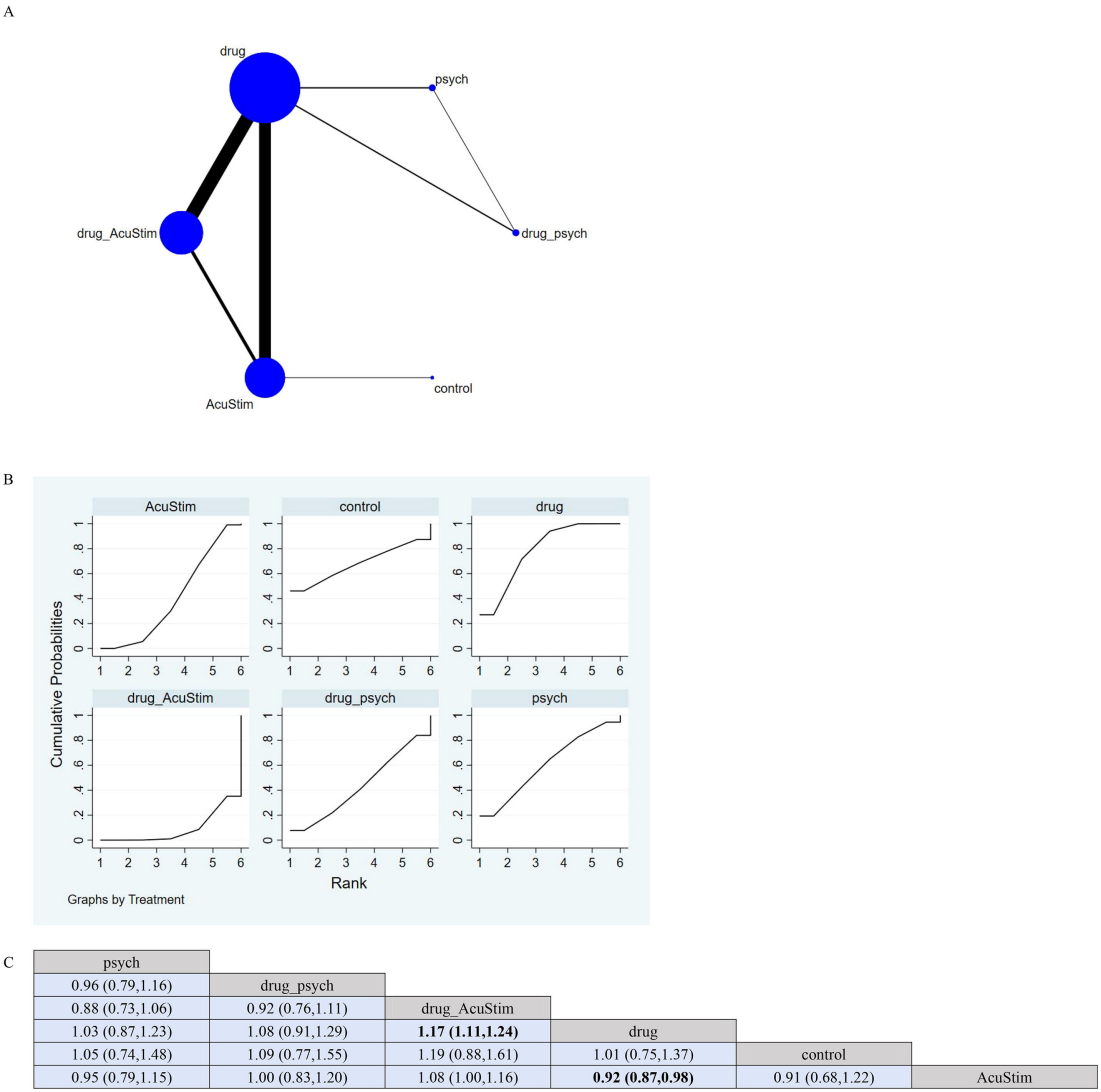
Network meta-analysis results for the clinical efficacy outcome across the three major intervention categories (drug, Acupoint Stimulation, and psychotherapy). **(A)** Network plot of comparisons. **(B)** SUCRA ranking plot of all interventions. **(C)** League table of pairwise comparisons with RRs and 95% CrIs.

Clinical efficacy was assessed in 68 studies. 46 studies compared the various interventions, with 41 arms for drug, 22 for AcuStim, 24 for drug_AcuStim, 3 for psych, 3 for drug_psych, and 1 for control. The most studied intervention nodes were drug, followed in descending order by drug_AcuStim and AcuStim. Regarding direct comparisons, the three most frequent were drug versus drug_AcuStim, drug versus AcuStim, and AcuStim versus drug_AcuStim. The evidence network contained 7 direct comparisons between intervention nodes. Additionally, drug, AcuStim, and drug_AcuStim formed a closed loop, allowing for indirect effect estimation. **Figure 7A** displays the evidence network among the included interventions.

According to the SUCRA rankings, drug_AcuStim demonstrated the greatest clinical efficacy (SUCRA = 9.0%), followed by AcuStim (SUCRA = 40.4%) and drug_psych (SUCRA = 43.5%). The specific ranking plot is presented in **Figure 7B**.

In the league table, only two comparisons reached statistical significance: drug_AcuStim versus drug (RR = 1.17, 95% CrI: 1.11 to 1.24) and AcuStim versus drug (RR = 0.92, 95% CrI: 0.87 to 0.98), both favoring the former over drug. The league table is visually summarized in **Figure 7C**.

Global inconsistency across the network was not statistically significant (P = 0.134), with an I² of 0% indicating no heterogeneity. Two closed loops were detected: AcuStim–drug–drug_AcuStim and drug–drug_psych–psych. The 95% CrI for the second loop crossed 0, suggesting no significant inconsistency. The global inconsistency forest plot in **Supplementary Material 6** (**Supplementary Figure 1.5****),** and the clinical efficacy forest plot in **Supplementary Material 7** (**Supplementary Figure 2.5**).

##### 3.4.3.2 Pharmacotherapies

**Figure 8:**
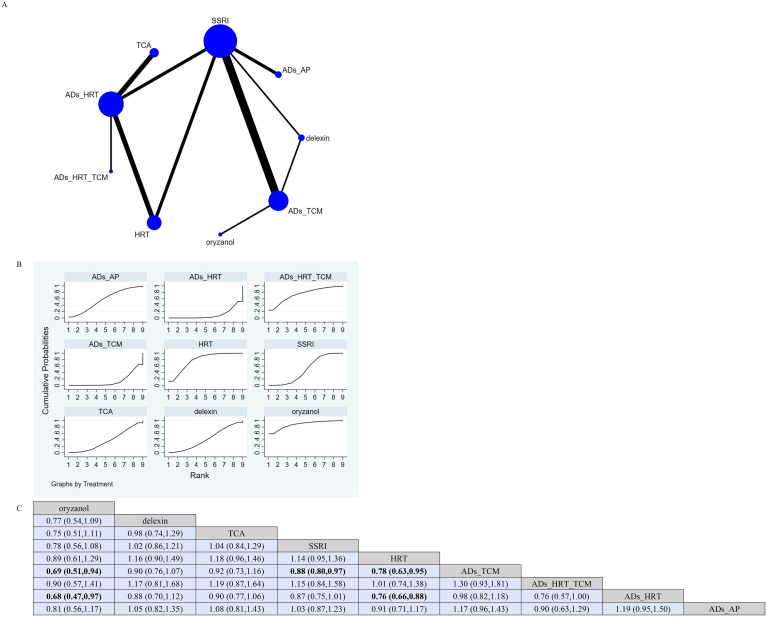
Network meta-analysis results for the clinical efficacy outcome within pharmacotherapy subgroups. **(A)** Network plot of comparisons. **(B)** SUCRA ranking plot of all interventions. **(C)** League table of pairwise comparisons with RRs and 95% CrIs.

Among the 68 studies with clinical efficacy measurements, all 17 evaluated the pharmacotherapy subgroups. The arm counts were 10 for SSRI, 7 for ADs_TCM, 7 for ADs_HRT, 3 for HRT, 3 for TCA, 2 for delexin, 2 for ADs_AP, 1 for ADs_HRT_TCM, and 1 for oryzanol. The most frequently investigated nodes were SSRI, with ADs_TCM and ADs_HRT followed. The most common direct comparisons involved SSRI versus ADs_TCM, HRT versus ADs_HRT, and ADs_HRT versus TCA. In the network, 10 direct pairwise relationships were identified. Two closed loops were observed among SSRI, ADs_HRT, and HRT, and among ADs_TCM, delexin, and SSRI, suggesting the presence of indirect evidence. The topology of the network is shown in **Figure 8A**.

The SUCRA values indicated that ADs_HRT (SUCRA = 10.2%) exhibited the greatest efficacy in improving clinical efficacy among the 9 interventions. It was followed by ADs_TCM (SUCRA = 13.4%) and TCA (SUCRA = 37.7%). **Figure 8B** depicts the overall intervention hierarchy.

From the league table, ADs_HRT exhibited significantly greater clinical efficacy than HRT (RR = 0.76, 95% CrI: 0.66 to 0.88) and oryzanol (RR = 0.68, 95% CrI: 0.47 to 0.97). ADs_TCM was also significantly superior to HRT (RR = 0.78, 95% CrI: 0.63 to 0.95), SSRI (RR = 0.88, 95% CrI: 0.80 to 0.97) and oryzanol (RR = 0.69, 95% CrI: 0.51 to 0.94). **Figure 8C** presents the detailed results.

The global inconsistency test showed no statistical significance (P = 0.698), with an I² of 4% indicating negligible heterogeneity. Two closed loops were identified within the network: ADs_TCM–SSRI–delexin and ADs_HRT–HRT–SSRI. The 95% CrIs for both loops contained 0, suggesting that loop inconsistency was not significant. Detailed results of the global inconsistency forest plot and the clinical efficacy forest plot are available in **Supplementary Materials 6**, **7**, respectively (**Supplementary Figures 1.6, S2.6**).

#### 3.4.4 Adverse events

##### 3.4.4.1 Three major intervention categories

**Figure 9:**
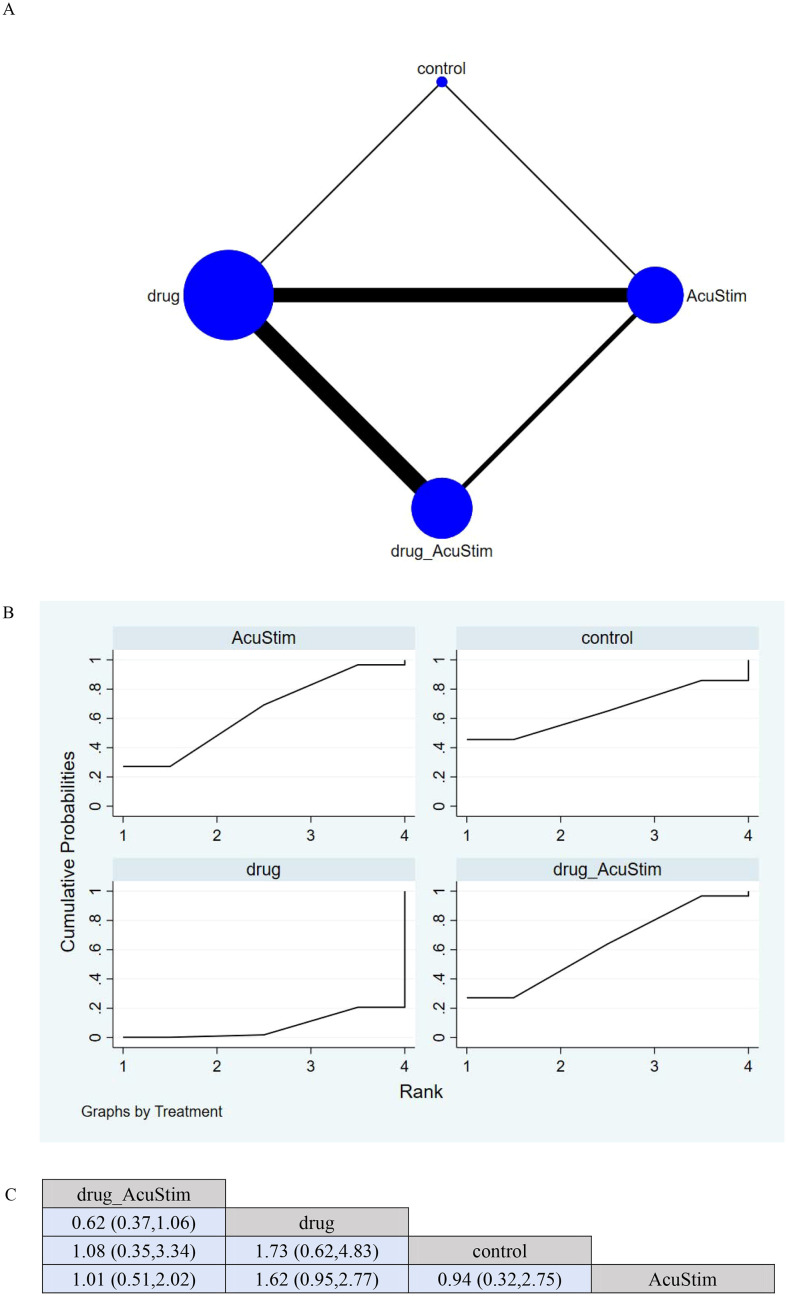
Network meta-analysis results for the AE outcome across the three major intervention categories (drug, Acupoint Stimulation, and psychotherapy). **(A)** Network plot of comparisons. **(B)** SUCRA ranking plot of all interventions. **(C)** League table of pairwise comparisons with RRs and 95% CrIs.

A total of 34 studies examined AEs. Of these, all 21 addressed the three major intervention categories, yielding 19 arms for drug, 11 for AcuStim, 12 for drug_AcuStim, and 2 for control. The nodes with the highest frequency were drug, followed by drug_AcuStim and AcuStim. The most prevalent pairwise comparisons were drug versus drug_AcuStim, drug versus AcuStim, and AcuStim versus drug_AcuStim. A total of 5 direct comparisons were mapped within the intervention network. The nodes drug, AcuStim, drug_AcuStim and control constituted a closed loop, indicating a connected evidence structure. A graphical representation of the network is available in **Figure 9A**.

Among the 4 interventions, control had the highest SUCRA value (SUCRA = 65.5%) for AE, followed by AcuStim (SUCRA = 64.4%) and drug_AcuStim (SUCRA = 62.6%). The ranking diagram is presented in **Figure 9B**. The league table showed no statistically significant differences among the interventions, as all 95% CrIs included 1, indicating comparable safety profiles across treatment groups. **Figure 9C** offers a detailed view of the league table.

With a p-value of 0.772, the global inconsistency test showed no statistical significance, and the I² statistic of 2% pointed to low heterogeneity. The network contained 2 closed loops: AcuStim–control–drug and AcuStim–drug–drug_AcuStim. The 95% CrIs for all loops crossed 0, implying that loop inconsistency was not significant. **Supplementary Material 6** provides the global inconsistency forest plot (**Supplementary Figure 1.7**), and **Supplementary Material 7** provides the AE forest plot (**Supplementary Figure 2.7**).

##### 3.4.4.2 Pharmacotherapies

**Figure 10:**
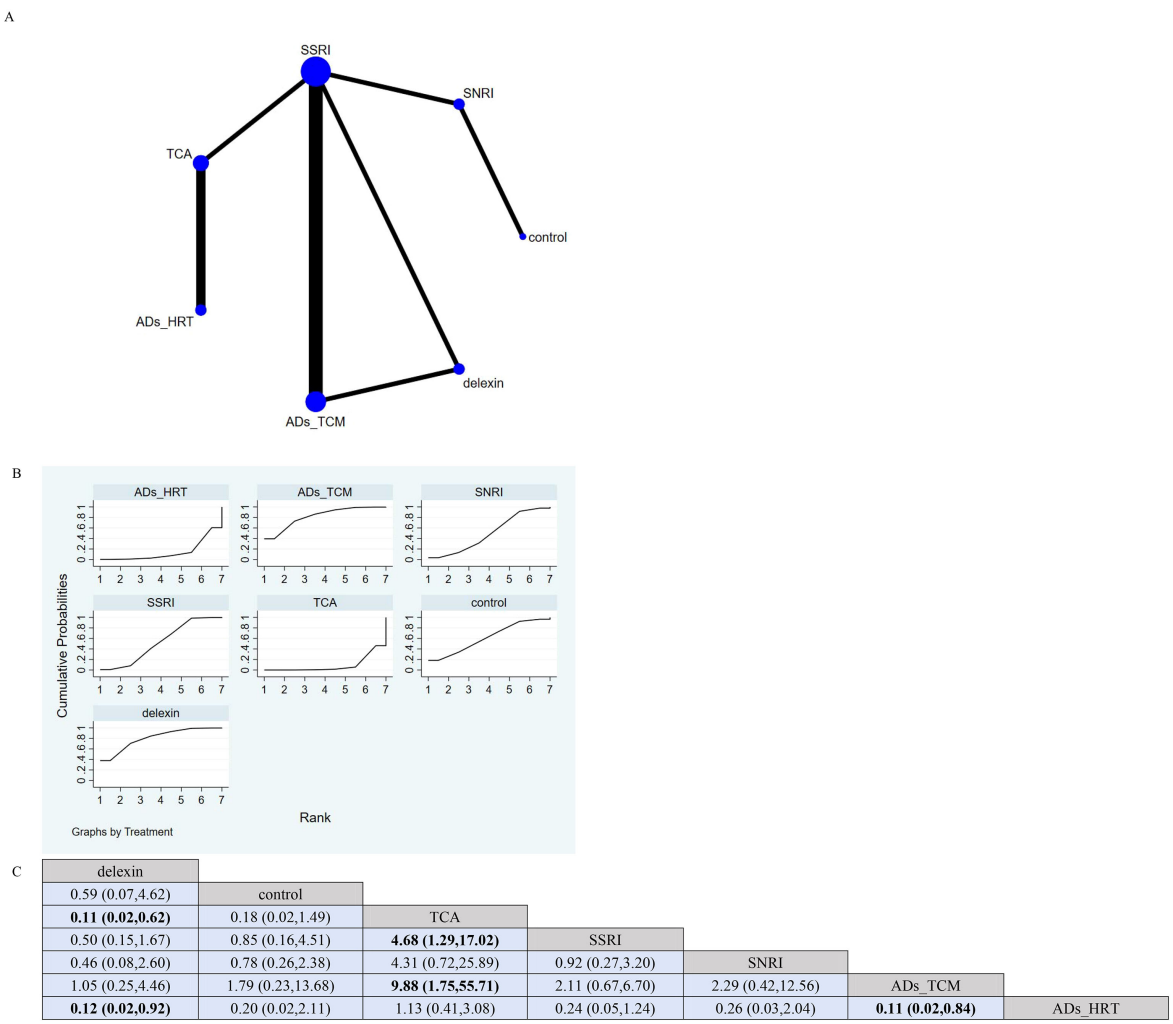
Network meta-analysis results for the AE outcome within pharmacotherapy subgroups. **(A)** Network plot of comparisons. **(B)** SUCRA ranking plot of all interventions. **(C)** League table of pairwise comparisons with RRs and 95% CrIs.

The AEs outcome was reported in 34 studies, of which all 10 compared the pharmacotherapy subgroups. The intervention arms included 6 for SSRI, 4 for ADs_TCM, 3 for TCA, 2 for SNRI, 2 for delexin, 2 for ADs_HRT, and 1 for control. SSRI was the most frequently examined node, with ADs_TCM and TCA as the next most common. The most frequent direct comparisons were between SSRI and ADs_TCM, and between TCA and ADs_HRT. The network comprised 7 direct evidence links. Indirect evidence was available through the closed loop formed by ADs_TCM, delexin, and SSRI. The network connections are visualized in **Figure 10A**.

The SUCRA ranking suggested that ADs_TCM (SUCRA = 82%) was the most promising intervention for AE. delexin (SUCRA = 80.7%) and control (SUCRA = 61.5%) ranked next. **Figure 10B** visualizes the comparative ranking.

In the league table, delexin demonstrated a significantly lower risk of adverse events compared with TCA (RR = 0.11, 95% CrI: 0.02 to 0.62) and ADs_HRT (RR = 0.12, 95% CrI: 0.02 to 0.92). In contrast, TCA was associated with a significantly higher risk of adverse events relative to SSRI (RR = 4.68, 95% CrI: 1.29 to 17.02) and ADs_TCM (RR = 9.88, 95% CrI: 1.75 to 55.71). Additionally, ADs_TCM showed a significantly lower AE risk than ADs_HRT (RR = 0.11, 95% CrI: 0.02 to 0.84). **Figure 10C** presents the detailed results.

The global inconsistency was not significant (P = 0.744), and the I² value of 4% implied a low level of heterogeneity. One closed loop was identified: ADs_TCM–SSRI–delexin. The 95% CrI for this loop contained 0, suggesting that loop inconsistency was not significant. Results are presented as follows: global inconsistency in **Supplementary Material 6** (**Supplementary Figure 1.8**), and AE forest plot in **Supplementary Material 7** (**Supplementary Figure 2.8**).

#### 3.4.5 Kupperman index

**Figure 11:**
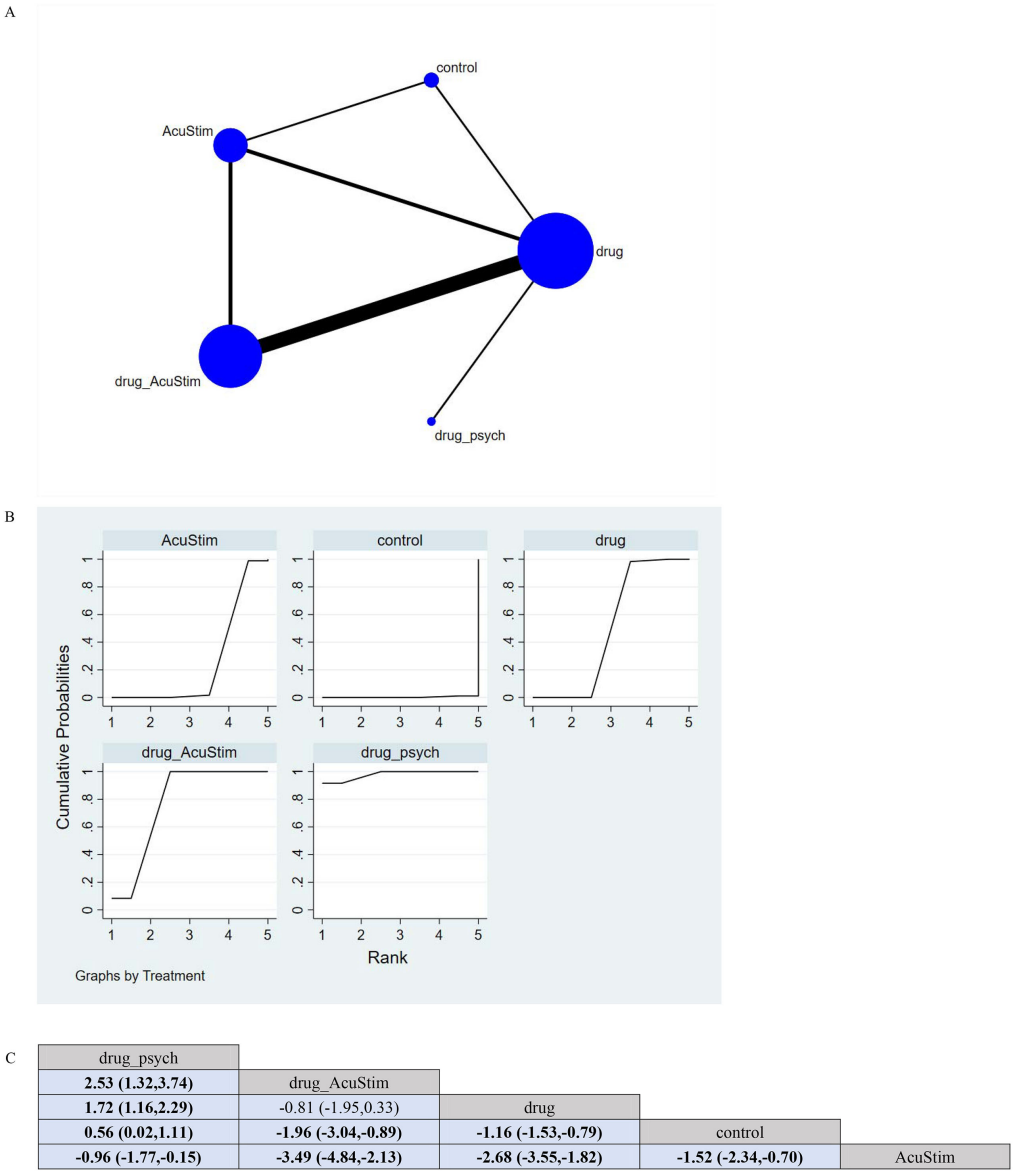
Network meta-analysis results for the KI outcome across the three major intervention categories (drug, Acupoint Stimulation, and psychotherapy). **(A)** Network plot of comparisons. **(B)** SUCRA ranking plot of all interventions. **(C)** League table of pairwise comparisons with SMDs and 95% CrIs.

In terms of KI, 34 studies were identified. Among them, all 27 focused on the three major intervention categories, with 22 arms assigned to drug, 18 to drug_AcuStim, 9 to AcuStim, 4 to control, and 2 to drug_psych. In terms of node frequency, drug ranked highest, followed by drug_AcuStim and AcuStim. Regarding direct evidence, the most common comparisons were drug versus drug_AcuStim. Among the various interventions, 6 direct pairwise comparisons were identified. A closed loop involving drug, AcuStim, drug_AcuStim and control provided a pathway for indirect comparison. **Figure 11A** illustrates the network of direct comparisons.

In terms of SUCRA values, drug_psych (SUCRA = 97.9%) outperformed all other interventions for KI, followed by drug_AcuStim (SUCRA = 77.1%) and drug (SUCRA = 49.6%). The specific ranking plot is shown in **Figure 11B**.

From the league table, drug_psych consistently outperformed all other interventions—drug_AcuStim (SMD = 2.53, 95% CrI: 1.32 to 3.74), drug (SMD = 1.72, 95% CrI: 1.16 to 2.29), control (SMD = 0.56, 95% CrI: 0.02 to 1.11), and AcuStim (SMD = -0.96, 95% CrI: -1.77 to -0.15). drug_AcuStim was significantly superior to control (SMD = -1.96, 95% CrI: -3.04 to -0.89) and AcuStim (SMD = -3.49, 95% CrI: -4.84 to -2.13), while drug was significantly more effective than both control (SMD = -1.16, 95% CrI: -1.53 to -0.79) and AcuStim (SMD = -2.68, 95% CrI: -3.55 to -1.82). AcuStim also showed significantly better efficacy than control (SMD = -1.52, 95% CrI: -2.34 to -0.70). **Figure 11C** presents the detailed results.

A p-value of 0.109 suggested that global inconsistency was not significant, while an I² of 4% reflected low heterogeneity across the network. A total of 2 closed loops were observed: AcuStim–control–drug and AcuStim–drug–drug_AcuStim. The 95% CrI for the second loop contained 0, suggesting no significant loop inconsistency. **Supplementary Material 6** (**Supplementary Figure 1.9**) presents the global inconsistency forest plot, and **Supplementary Material 7** (**Supplementary Figure 2.9**) displays the KI forest plot.

#### 3.4.6 Self-rating depression scale

**Figure 12:**
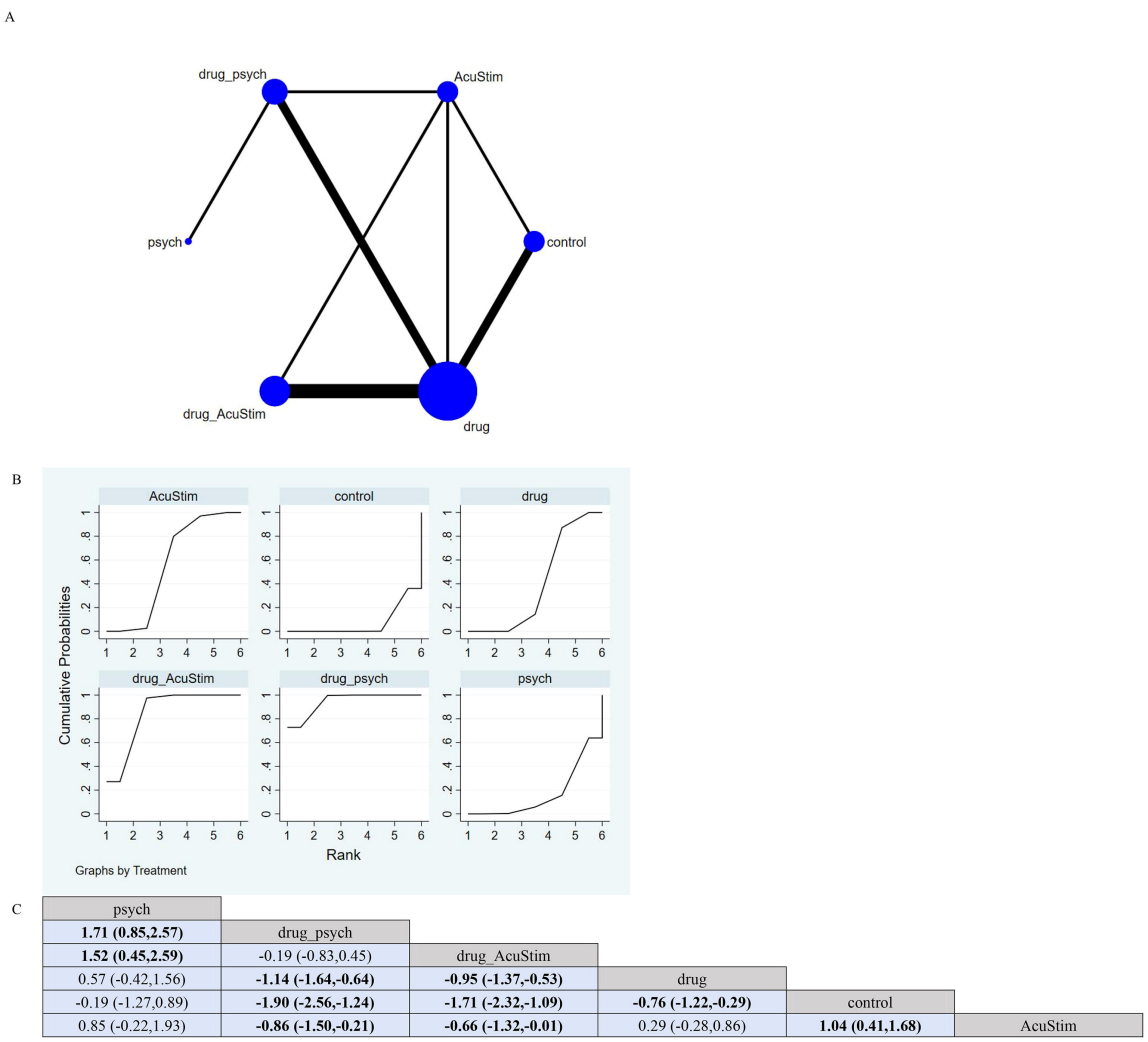
Network meta-analysis results for the SDS outcome across the three major intervention categories (drug, Acupoint Stimulation, and psychotherapy). **(A)** Network plot of comparisons. **(B)** SUCRA ranking plot of all interventions. **(C)** League table of pairwise comparisons with SMDs and 95% CrIs.

A total of 17 studies provided data on the SDS. Among them, all 14 focused on the three major intervention categories. The arms were distributed as 11 for drug, 5 for drug_AcuStim, 5 for drug_psych, 4 for control, 3 for AcuStim, and 1 for psych. The most commonly evaluated nodes were drug, followed in order by drug_AcuStim and drug_psych. The most frequent head-to-head comparisons were drug versus drug_AcuStim, drug versus control, and drug versus drug_psych. The overall network included 8 direct relationships. Two closed loops were identified in the network: one formed by drug, AcuStim, control and drug_AcuStim; and another formed by drug, control, drug_psych and AcuStim, indicating the presence of indirect evidence connections. The structure of the evidence network can be seen in **Figure 12A**.

According to the SUCRA probability estimates, drug_psych (SUCRA = 94.5%) was ranked as the most effective intervention for SDS. It was followed by drug_AcuStim (SUCRA = 84.9%) and AcuStim (SUCRA = 55.9%). The overall intervention hierarchy is depicted in **Figure 12B**.

In the league table, drug_psych demonstrated significantly superior efficacy in reducing SDS scores compared with psych (SMD = 1.71, 95% CrI: 0.85 to 2.57), drug (SMD = -1.14, 95% CrI: -1.64 to -0.64), control (SMD = -1.90, 95% CrI: -2.56 to -1.24), and AcuStim (SMD = -0.86, 95% CrI: -1.50 to -0.21). drug_AcuStim also showed significantly greater efficacy than psych (SMD = 1.52, 95% CrI: 0.45 to 2.59), drug (SMD = -0.95, 95% CrI: -1.37 to -0.53), control (SMD = -1.71, 95% CrI: -2.32 to -1.09), and AcuStim (SMD = -0.66, 95% CrI: -1.32 to -0.01). drug significantly outperformed control (SMD = -0.76, 95% CrI: -1.22 to -0.29), and AcuStim was significantly superior to control (SMD = 1.04, 95% CrI: 0.41 to 1.68). No other significant differences were observed. **Figure 12C** presents the detailed results.

The test for global inconsistency yielded a non-significant result (P = 0.910), and the I² value of 4% was indicative of low heterogeneity. Three closed loops were identified within the network: AcuStim–drug–drug_AcuStim, AcuStim–control–drug, and AcuStim–drug–drug_psych. The 95% CrIs for all loops included 0, suggesting no evidence of significant loop inconsistency. The global inconsistency forest plot and the SDS forest plot are presented in **Supplementary Materials 6**, **7**, respectively (**Supplementary Figures 1.10, S2.10**).

#### 3.4.7 Self-rating anxiety scale

**Figure 13:**
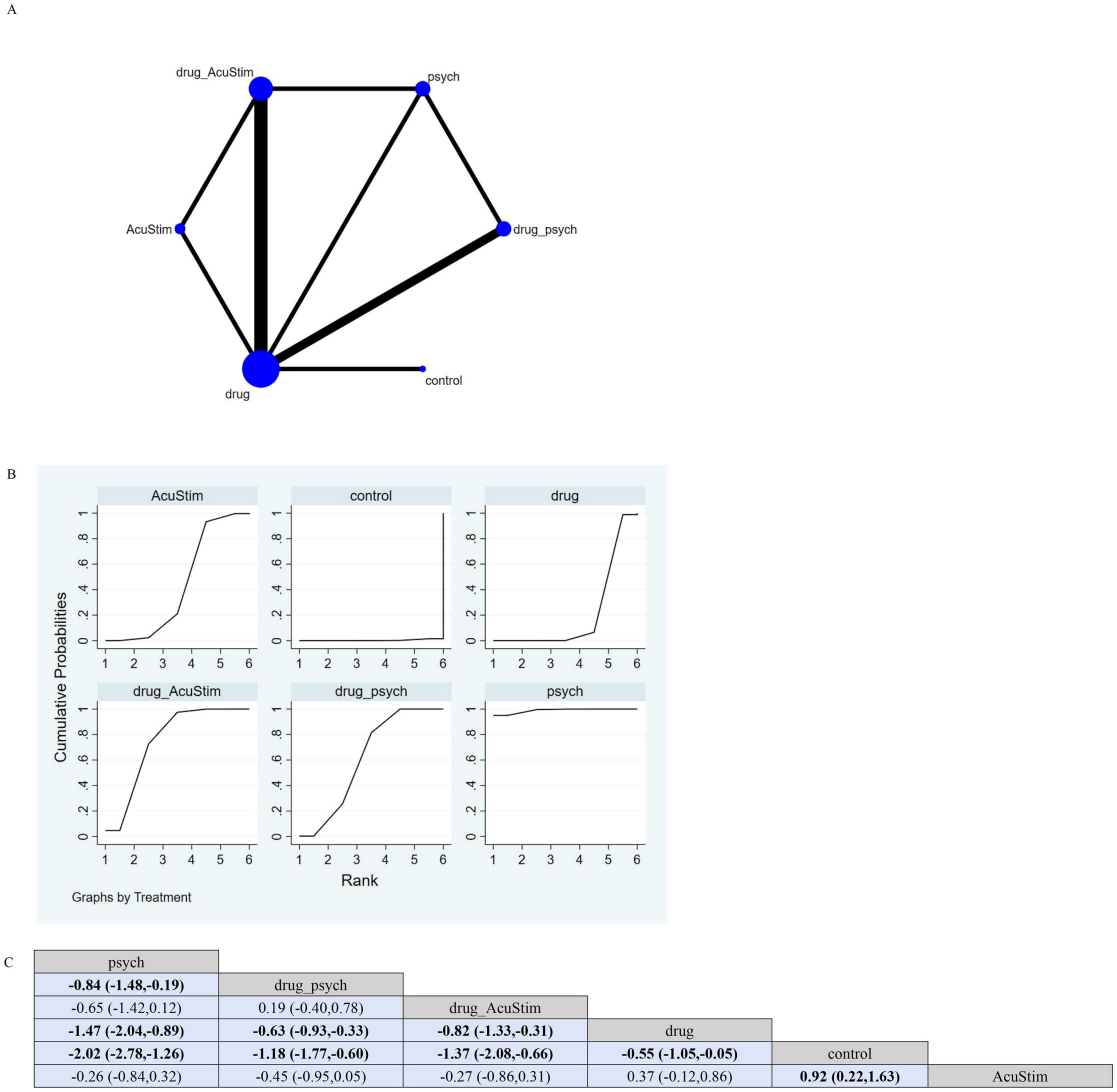
Network meta-analysis results for the SAS outcome across the three major intervention categories (drug, Acupoint Stimulation, and psychotherapy). **(A)** Network plot of comparisons. **(B)** SUCRA ranking plot of all interventions. **(C)** League table of pairwise comparisons with SMDs and 95% CrIs.

A total of 11 studies contributed SAS data. Of these, all 9 examined the three major intervention categories. The arms were 7 for drug, 4 for drug_AcuStim, 3 for drug_psych, 3 for psych, 1 for AcuStim, and 1 for control. Drug was the most frequently studied intervention, with drug_AcuStim being the next most frequent. The most common direct comparisons observed were drug versus drug_AcuStim, and drug versus drug_psych. The network featured 8 direct pairwise comparisons. An indirect comparison loop was established among drug, AcuStim, drug_AcuStim, psych and drug_psych. **Figure 13A** provides an overview of the network topology.

The SUCRA analysis identified psych (SUCRA = 98.9%) as the top-performing intervention for SAS among the 6 treatments. drug_AcuStim (SUCRA = 74.9%) and drug_psych (SUCRA = 61.5%) were the next most effective. **Figure 13B** illustrates the ranking diagram.

From the league table, psych emerged as the most effective intervention, significantly outperforming drug_psych (SMD = -0.84, 95% CrI: -1.48 to -0.19), drug (SMD = -1.47, 95% CrI: -2.04 to -0.89), and control (SMD = -2.02, 95% CrI: -2.78 to -1.26). In the second tier, drug_psych showed significantly better efficacy than drug (SMD = -0.63, 95% CrI: -0.93 to -0.33) and control (SMD = -1.18, 95% CrI: -1.77 to -0.60), while drug_AcuStim also demonstrated significant superiority over drug (SMD = -0.82, 95% CrI: -1.33 to -0.31) and control (SMD = -1.37, 95% CrI: -2.08 to -0.66). Among the remaining interventions, drug significantly outperformed control (SMD = -0.55, 95% CrI: -1.05 to -0.05), and AcuStim was significantly more effective than control (SMD = 0.92, 95% CrI: 0.22 to 1.63). **Figure 13C** presents the detailed results.

The test for global inconsistency yielded a significant result (P < 0.001), although the I² value of 5% indicated low overall heterogeneity. Three closed loops were identified: drug–drug_psych–psych, drug–drug_AcuStim–psych, and AcuStim–drug–drug_AcuStim. The 95% CrIs for the third loop contained 0, suggesting no significant loop inconsistency. Subgroup analyses with drug as the reference revealed that both drug_AcuStim and drug_psych significantly outperformed drug, with low within-group heterogeneity (I² = 0%). The significant between-subgroup difference (p = 0.030) indicates that the type of combination therapy is probably a source of inconsistency in the SAS analysis. Detailed results of the global inconsistency test, SAS forest plot and subgroup analysis, are available in **Supplementary Materials 6**–**8**, respectively (**Supplementary Figure 1.11, S2.11, S3.2**).

#### 3.4.8 Pittsburgh sleep quality index

**Figure 14:**
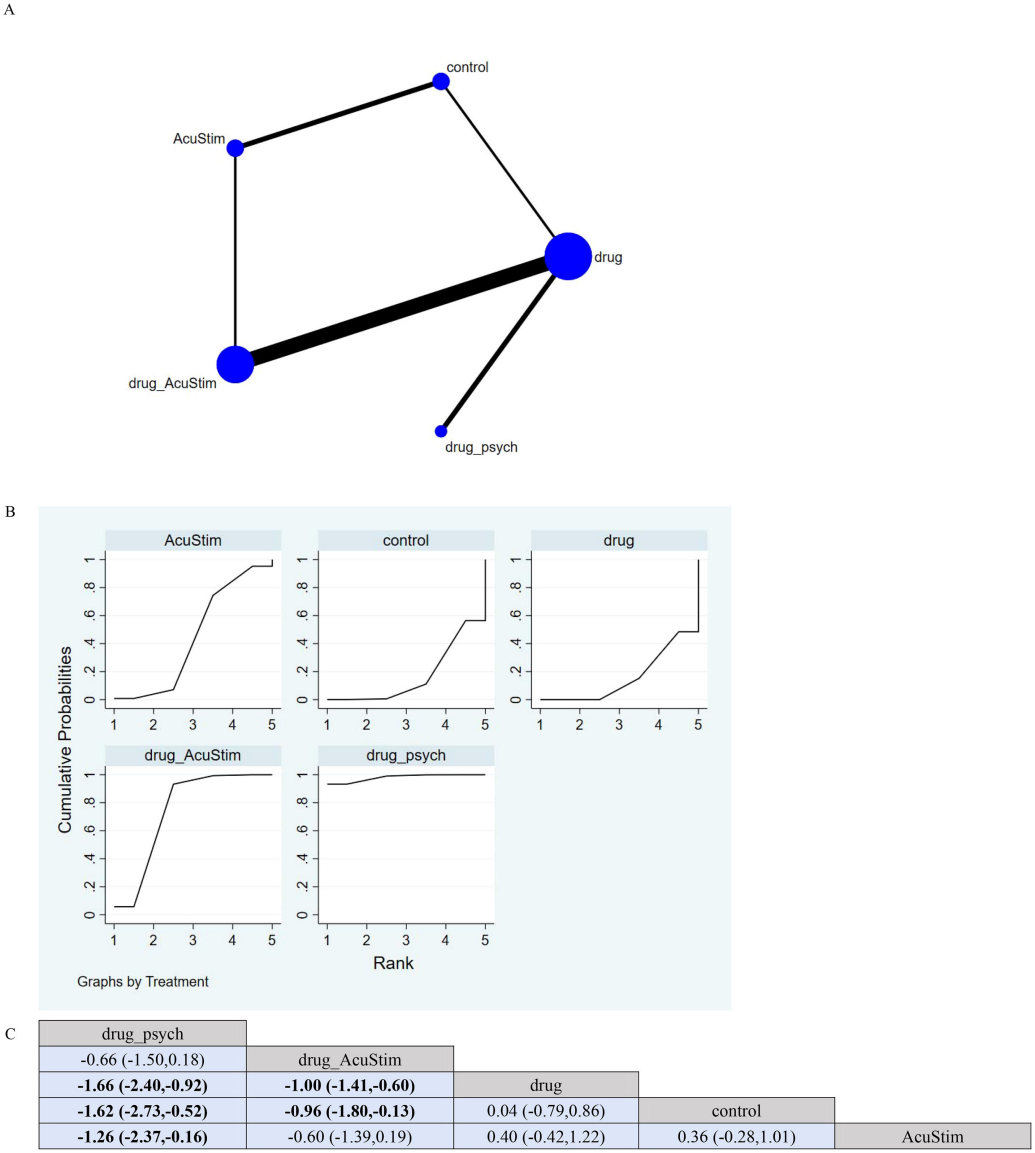
Network meta-analysis results for the PSQI outcome across the three major intervention categories (drug, Acupoint Stimulation, and psychotherapy). **(A)** Network plot of comparisons. **(B)** SUCRA ranking plot of all interventions. **(C)** League table of pairwise comparisons with SMDs and 95% CrIs.

Considering PSQI, 17 studies were included. A subset of 12 studies assessed the three major intervention categories, with arm numbers of 9 for drug, 7 for drug_AcuStim, 3 for AcuStim, 3 for control, and 2 for drug_psych. The network featured drug as the most studied node, followed by drug_AcuStim. The most frequent pairwise comparisons were between drug and drug_AcuStim. A total of 5 pairs of direct evidence links were established in the network. Among them, a closed loop comprising drug, AcuStim, control and drug_AcuStim enabled indirect comparisons. The intervention network is mapped out in **Figure 14A**.

Based on the SUCRA values, drug_psych (SUCRA = 98.1%) was the most effective in reducing PSQI, with drug_AcuStim (SUCRA = 74.6%) and AcuStim (SUCRA = 44.4%) ranking second and third. The specific ranking plot is displayed in **Figure 14B**.

From the league table, drug_psych ranked as the most effective intervention, significantly outperforming drug (SMD = -1.66, 95% CrI: -2.40 to -0.92), control (SMD = -1.62, 95% CrI: -2.73 to -0.52), and AcuStim (SMD = -1.26, 95% CrI: -2.37 to -0.16). In the second tier, drug_AcuStim also demonstrated significantly better efficacy than drug (SMD = -1.00, 95% CrI: -1.41 to -0.60) and control (SMD = -0.96, 95% CrI: -1.80 to -0.13). A detailed presentation of the results is available in **Figure 14C**.

Global inconsistency was found to be non-significant (P = 0.738), with an I² of 0% suggesting minimal heterogeneity. One closed loop was present in the network: AcuStim–control–drug–drug_AcuStim. The 95% CrI included 0, pointing to a lack of significant loop inconsistency. The global inconsistency forest plot and the PSQI forest plot are presented in **Supplementary Materials 6**, **7**, respectively (**Supplementary Figures 1.12, S2.12**).

#### 3.4.9 follicle-stimulating hormone

**Figure 15:**
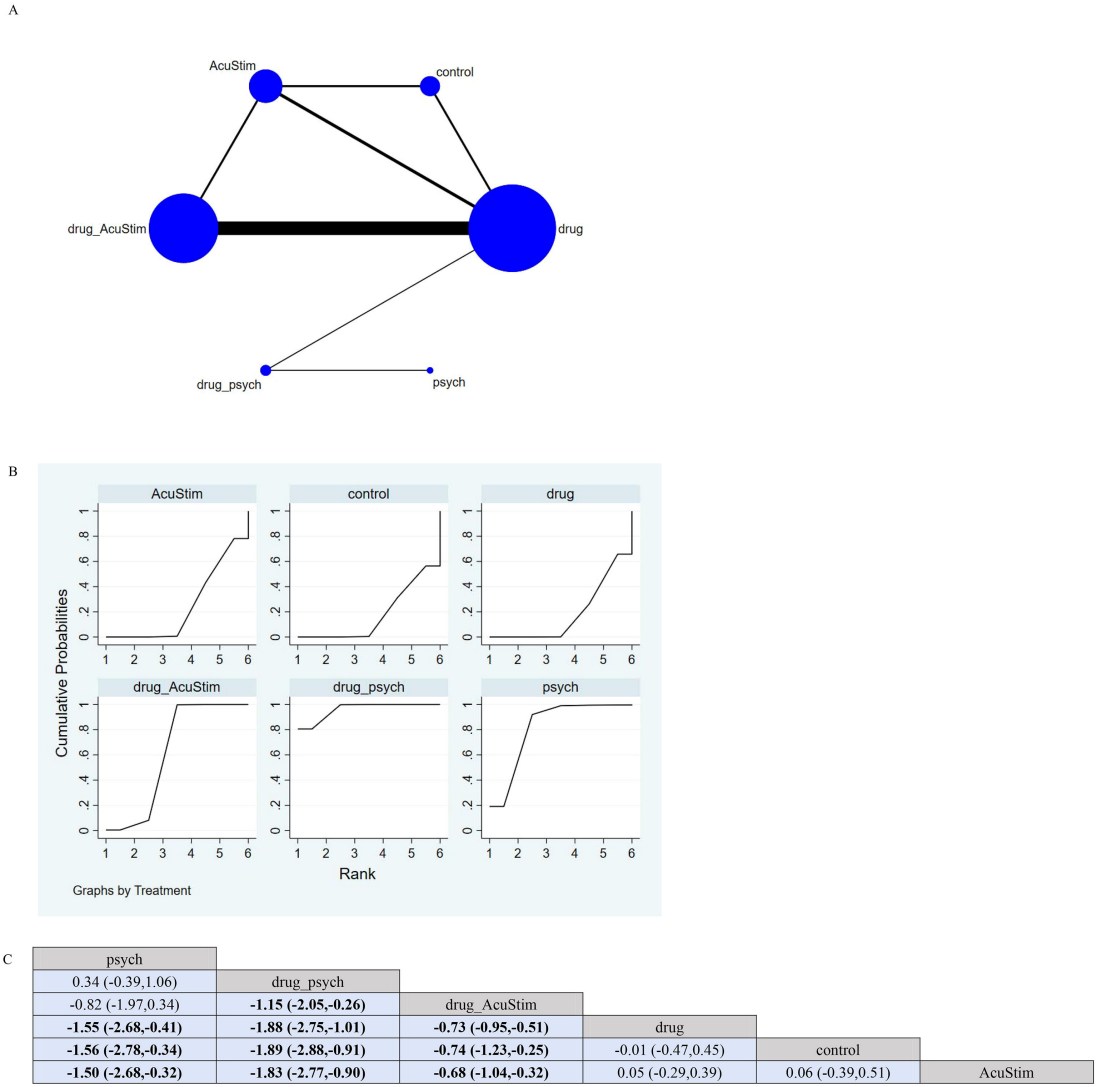
Network meta-analysis results for the FSH outcome across the three major intervention categories (drug, Acupoint Stimulation, and psychotherapy). **(A)** Network plot of comparisons. **(B)** SUCRA ranking plot of all interventions. **(C)** League table of pairwise comparisons with SMDs and 95% CrIs.

FSH data were available from 33 studies. Among them, all 24 evaluated the three major intervention categories, and the intervention arms were 19 for drug, 15 for drug_AcuStim, 7 for AcuStim, 4 for control, 2 for drug_psych, and 1 for psych. The most investigated nodes were drug, with drug_AcuStim and AcuStim following closely. The most common direct evidence links were drug versus drug_AcuStim. Within the evidence network, 7 direct comparisons were observed. The closed loop consisting of drug, control, AcuStim, and drug_AcuStim suggested a well-connected evidence base. **Figure 15A** depicts the relationships among the interventions.

The SUCRA ranking demonstrated that drug_psych (SUCRA = 96.1%) had the highest probability of being the most effective intervention for lowering FSH levels. It was followed by psych (SUCRA = 81.9%) and drug_AcuStim (SUCRA = 61.6%). **Figure 15B** presents the overall ranking diagram.

From the league table, the three top-ranked interventions—drug_psych, psych, and drug_AcuStim—consistently outperformed the remaining interventions in reducing FSH levels. drug_psych was significantly more effective than drug_AcuStim (SMD = -1.15, 95% CrI: -2.05 to -0.26), drug (SMD = -1.88, 95% CrI: -2.75 to -1.01), control (SMD = -1.89, 95% CrI: -2.88 to -0.91), and AcuStim (SMD = -1.83, 95% CrI: -2.77 to -0.90), while psych showed significant superiority over drug (SMD = -1.55, 95% CrI: -2.68 to -0.41), control (SMD = -1.56, 95% CrI: -2.78 to -0.34), and AcuStim (SMD = -1.50, 95% CrI: -2.68 to -0.32). drug_AcuStim also demonstrated significantly better efficacy than drug (SMD = -0.73, 95% CrI: -0.95 to -0.51), control (SMD = -0.74, 95% CrI: -1.23 to -0.25), and AcuStim (SMD = -0.68, 95% CrI: -1.04 to -0.32). Detailed findings are provided in **Figure 15C**.

The global inconsistency analysis produced a p-value of 0.696, indicating no significant inconsistency, and the I² of 0% suggested minimal heterogeneity. The network featured 2 closed loops: AcuStim–control–drug and AcuStim–drug–drug_AcuStim. All 95% CrIs crossed 0, indicating that loop inconsistency was not statistically significant. For detailed results, see **Supplementary Material 6** (**Supplementary Figure 1.13**) for global inconsistency, and **Supplementary Material 7** (**Supplementary Figure 2.13**) for the FSH forest plot.

#### 3.4.10 Luteinizing hormone

**Figure 16:**
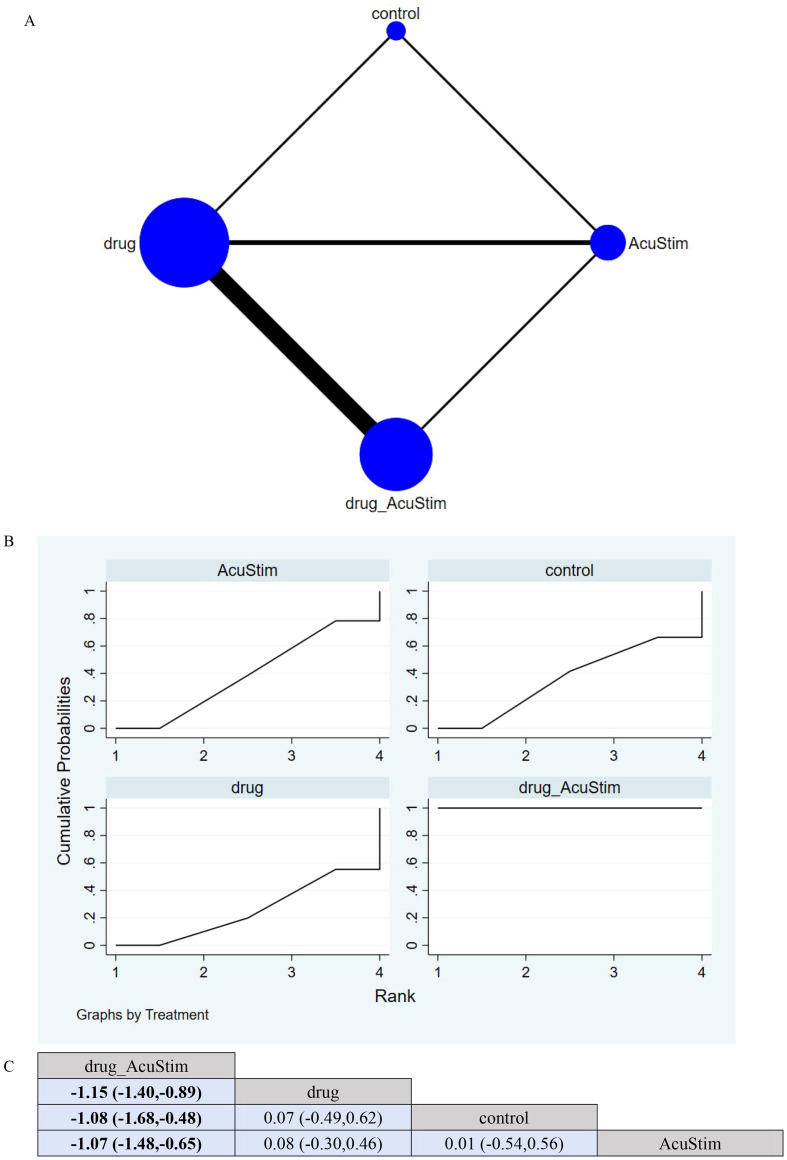
Network meta-analysis results for the LH outcome across the three major intervention categories (drug, Acupoint Stimulation, and psychotherapy). **(A)** Network plot of comparisons. **(B)** SUCRA ranking plot of all interventions. **(C)** League table of pairwise comparisons with SMDs and 95% CrIs.

Of the 30 studies that measured LH, all 25 investigated the three major intervention categories. The breakdown of arms was 21 for drug, 17 for drug_AcuStim, 8 for AcuStim, and 4 for control. Drug emerged as the most frequently studied intervention, followed by drug_AcuStim and AcuStim. The most prevalent comparisons were drug versus drug_AcuStim, followed by drug versus AcuStim. In total, 5 direct comparisons were identified across the interventions. The indirect evidence was captured by the closed loop formed by drug, AcuStim, control, and drug_AcuStim. A comprehensive network plot is given in **Figure 16A**.

Regarding SUCRA values, drug_AcuStim (SUCRA = 100%) showed the greatest benefit for lowering LH levels, followed by AcuStim (SUCRA = 39%) and control (SUCRA = 36%). The ranking plot is provided in **Figure 16B**.

In the league table, drug_AcuStim demonstrated significantly superior efficacy in lowering LH levels compared with drug (SMD = -1.15, 95% CrI: -1.40 to -0.89), control (SMD = -1.08, 95% CrI: -1.68 to -0.48), and AcuStim (SMD = -1.07, 95% CrI: -1.48 to -0.65). **Figure 16C** offers a detailed summary of the results.

No significant global inconsistency was detected (P = 0.339), and the I² value of 13% pointed to a low degree of heterogeneity. A total of 2 closed loops were detected: AcuStim–drug–drug_AcuStim and AcuStim–control–drug. The 95% CrIs for all loops contained 0, suggesting that the direct and indirect evidence were consistent. The corresponding figures are: global inconsistency forest plot (**Supplementary Material 6**, **Supplementary Figure 1.14**), and LH forest plot (**Supplementary Material 7**, **Supplementary Figure 2.14**).

#### 3.4.11 Estradiol

**Figure 17:**
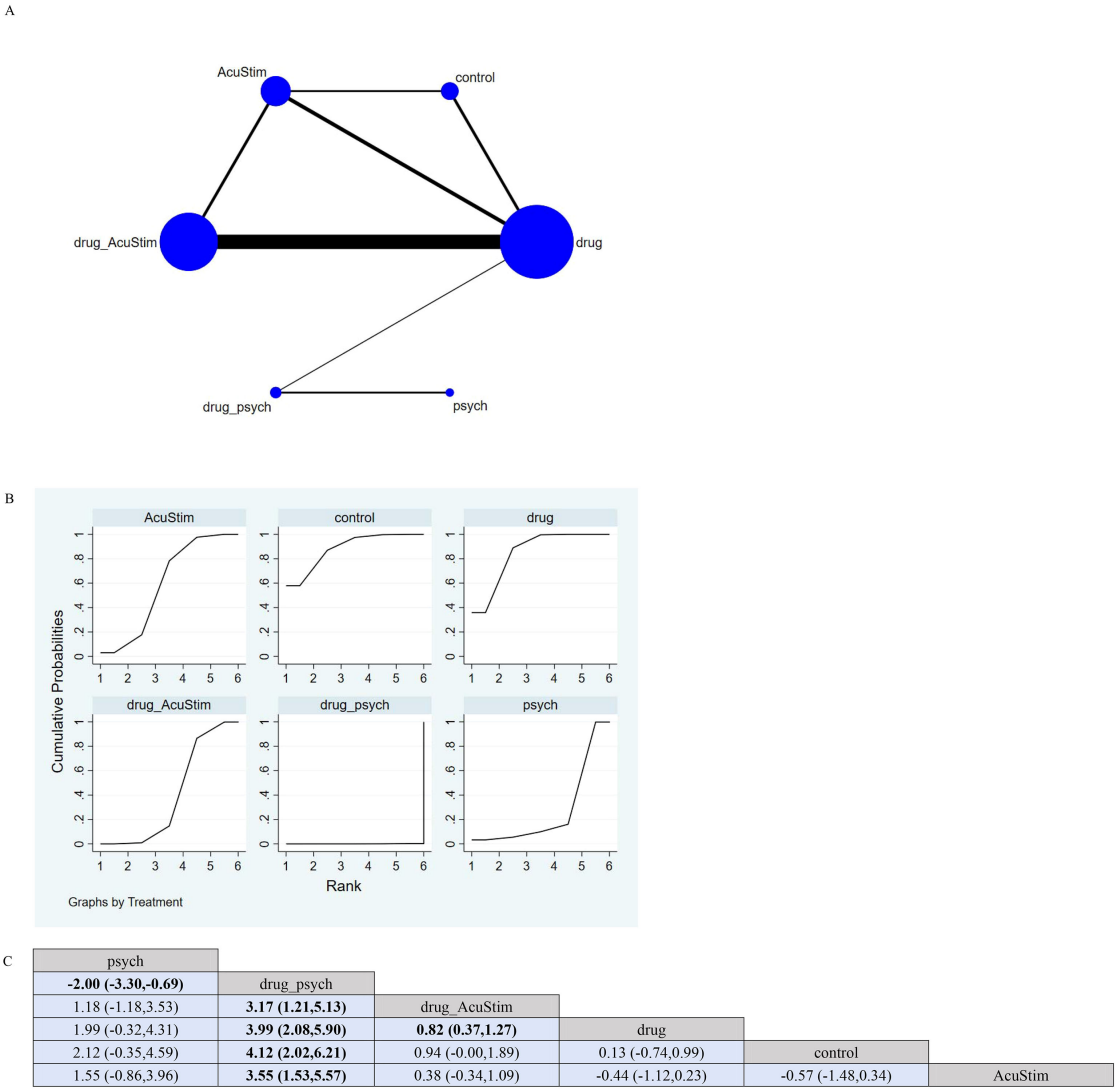
Network meta-analysis results for the E2 outcome across the three major intervention categories (drug, Acupoint Stimulation, and psychotherapy). **(A)** Network plot of comparisons. **(B)** SUCRA ranking plot of all interventions. **(C)** League table of pairwise comparisons with SMDs and 95% CrIs.

A total of 37 studies reported E2, and of these, all 28 compared the three major intervention categories. The arm frequencies were 22 for drug, 17 for drug_AcuStim, 8 for AcuStim, 5 for control, 3 for drug_psych, and 2 for psych. The nodes that appeared most frequently were drug, then drug_AcuStim, and then AcuStim. The most frequent direct comparisons were drug versus drug_AcuStim, drug versus control, and drug versus AcuStim. A total of 7 direct relationships were detected between the intervention nodes. Additionally, drug, AcuStim, control, and drug_AcuStim formed a closed loop, enabling indirect evidence synthesis. **Figure 17A** shows the network of treatment comparisons.

The SUCRA results indicated that drug_psych (SUCRA = 0.1%) was the preferred intervention for elevating E2 levels among the 6 treatments. psych (SUCRA = 26.9%) and drug_AcuStim (SUCRA = 40.4%) followed in rank. The specific intervention hierarchy is illustrated in **Figure 17B**.

In the league table, drug_psych demonstrated significantly superior efficacy in elevating E2 levels compared with psych (SMD = -2.00, 95% CrI: -3.30 to -0.69), drug_AcuStim (SMD = 3.17, 95% CrI: 1.21 to 5.13), drug (SMD = 3.99, 95% CrI: 2.08 to 5.90), control (SMD = 4.12, 95% CrI: 2.02 to 6.21), and AcuStim (SMD = 3.55, 95% CrI: 1.53 to 5.57). drug_AcuStim also showed significantly greater efficacy than drug (SMD = 0.82, 95% CrI: 0.37 to 1.27). The results are detailed in **Figure 17C**.

The p-value of 0.673 demonstrated that global inconsistency was not significant, and the I² of 4% was consistent with low heterogeneity. Two closed loops were identified: AcuStim–control–drug and AcuStim–drug–drug_AcuStim. The 95% CrI for the second loop contained 0, suggesting no significant loop inconsistency. **Supplementary Figure 1.15** in **Supplementary Material 6** shows the global inconsistency forest plot, and **Supplementary Figure 2.15** in **Supplementary Material 7** presents the E2 forest plot.

### 3.5 Comparison-adjusted funnel plot

**Figure 18:**
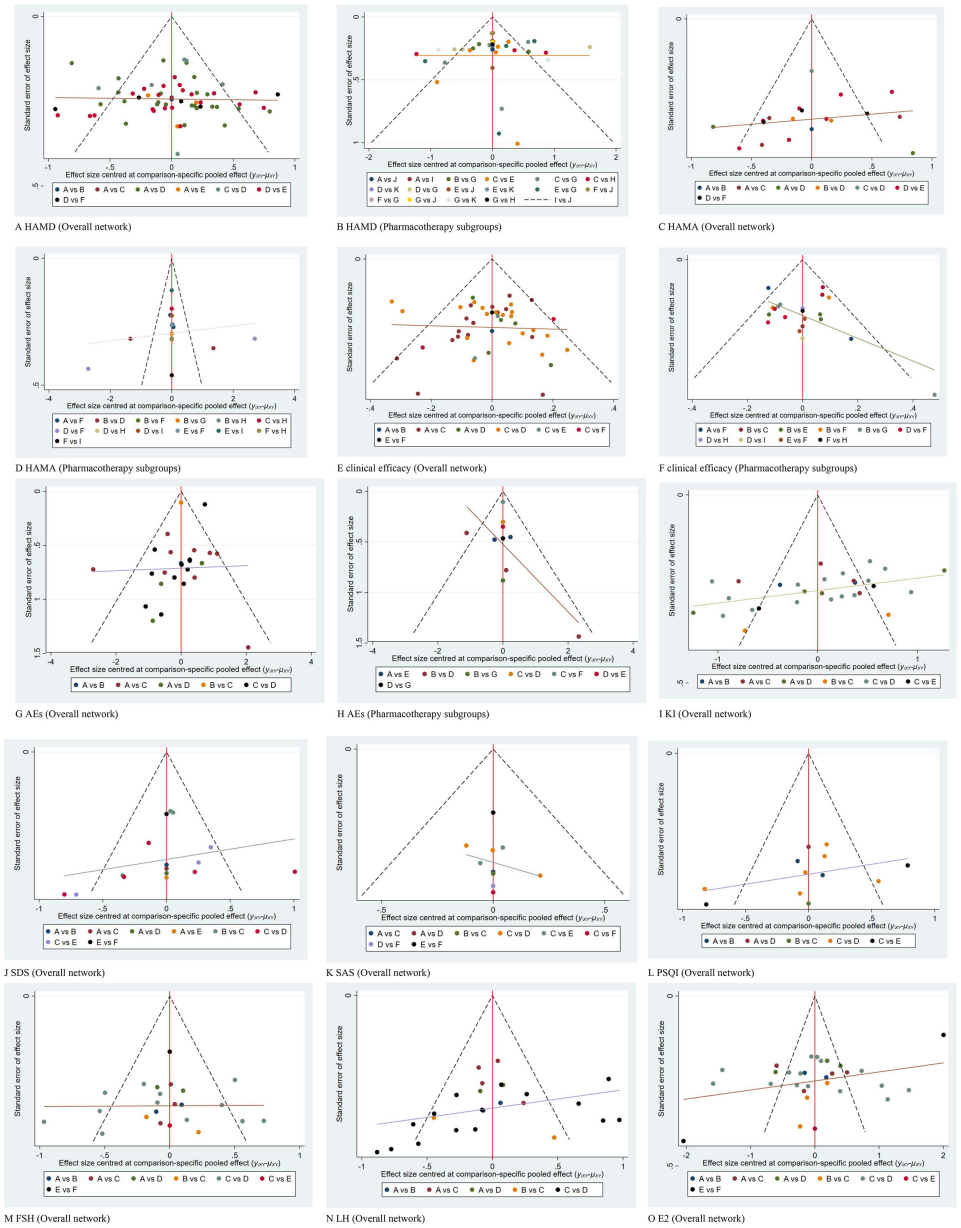
Adjusted funnel plots. **(A)** HAMD across the three major intervention categories. **(B)** HAMD within pharmacotherapy subgroups. **(C)** HAMA across the three major intervention categories. **(D)** HAMA within pharmacotherapy subgroups. **(E)** Clinical efficacy across the three major intervention categories. **(F)** Clinical efficacy within pharmacotherapy subgroups. **(G)** AEs across the three major intervention categories. **(H)** AEs within pharmacotherapy subgroups. **(I)** KI across the three major intervention categories. **(J)** SDS across the three major intervention categories. **(K)** SAS across the three major intervention categories. **(L)** PSQI across the three major intervention categories. **(M)** FSH across the three major intervention categories. **(N)** LH across the three major intervention categories. **(O)** E2 across the three major intervention categories.

We employed adjusted funnel plot (**Figure 18**) to evaluate the risk of bias in the included studies. The funnel plots for all studies appeared approximately symmetric, suggesting no significant risk of bias.

## 4 Discussion

This NMA synthesized data from 131 RCTs. In the overall network, combination therapies consistently showed the highest SUCRA probabilities across most outcomes. Specifically, drug_psych ranked first for reducing HAMD (SUCRA = 92.4%), KI (97.9%), SDS (94.5%), PSQI (98.1%), and FSH (96.1%), as well as for elevating E2 (SUCRA = 0.1%); AcuStim_psych ranked highest for HAMA reduction (SUCRA = 93.7%); psych for SAS reduction (SUCRA = 98.9%); drug_AcuStim for clinical efficacy (SUCRA = 9.0%) and LH reduction (SUCRA = 100%); and control ranked highest for safety (SUCRA = 65.5%). In pharmacotherapy subgroup analyses, ADs_TCM ranked highest for HAMD reduction (SUCRA = 87.2%) and safety (SUCRA = 82%), ADs_AP for HAMA reduction (SUCRA = 97.5%), and ADs_HRT for clinical efficacy (SUCRA = 10.2%). Although combination therapies generally ranked highly across multiple outcomes, the relative rankings should be interpreted in conjunction with the overall evidence and pairwise comparisons.

The distribution of evidence across intervention nodes was notably uneven in this NMA, despite a comprehensive literature search across eight Chinese and English databases. For instance, the “drug” node was supported by over 20 studies for most outcomes, whereas the “drug_psych” node—despite ranking highest in SUCRA for KI, PSQI, and FSH—rested on only 2 to 5 studies. In sparse networks, SUCRA estimates are highly sensitive to minor fluctuations in the underlying evidence base. Therefore, the consistently high SUCRA rankings of “drug_psych” should be interpreted with caution; they suggest a potential advantage for combination therapy, but the evidence remains preliminary and must be weighed against the corresponding league table estimates and their 95% CrIs. Overall, the network structure was adequately supported for most outcomes, with the principal concern being node–level evidence imbalance rather than global network sparsity.

To address clinical and methodological heterogeneity, we stratified interventions along two dimensions. In the overall network analysis, interventions were grouped into pharmacotherapy, acupoint stimulation, psychotherapy, and their various combinations. In subgroup analyses, we further stratified by specific drug classes (e.g., SSRIs, SNRIs, HRT, TCM), acupoint stimulation modalities (e.g., acupuncture, electroacupuncture, thread embedding), and psychotherapeutic approaches (e.g., CBT, mindfulness). However, the number of studies within each acupoint stimulation and psychotherapy subgroup was insufficient to support a reliable network meta-analysis. Global inconsistency tests indicated no significant inconsistency in most networks (overall I² < 50%), with two exceptions: the HAMA pharmacotherapy subgroup (P < 0.001) and the SAS overall network (P < 0.001). Further subgroup analyses revealed that, in the HAMA pharmacotherapy network (SSRI as reference), between–subgroup differences were not significant (p = 0.158), suggesting that the inconsistency was driven by outlier studies rather than drug–class differences. In the SAS overall network (drug as reference), between–subgroup differences were significant (p = 0.030), and both drug_AcuStim and drug_psych outperformed drug with minimal within–subgroup heterogeneity (I² = 0%), indicating that the inconsistency likely reflects the differential effects of combination versus monotherapy. Thus, the consistency assumption was supported in most networks, reinforcing the robustness of our main findings. For the two inconsistent networks, results should be interpreted with caution; the inconsistency is more plausibly attributed to the genuine superiority of combination strategies than to methodological bias.

This NMA identified drug_AcuStim as the most reliable strategy for improving clinical efficacy. It ranked first by SUCRA (9.0%) and significantly outperformed drug monotherapy (RR = 1.17, 95% CrI: 1.11 to 1.24), with 24 studies and minimal heterogeneity (I² = 0%)—representing the strongest efficacy evidence in this NMA. Acupuncture may enhance ovarian function by modulating the hypothalamic-pituitary-ovarian (HPO) axis, reproductive–related neuropeptides (e.g., kisspeptin, neuropeptide Y, and gonadotropin-releasing hormone), and neurotransmitters (e.g., γ-aminobutyric acid, serotonin, glutamate, dopamine), while also influencing peripheral–to–central sensory pathways and autonomic activity (150). A U.S. study reported that among women treated with acupuncture for menopausal depression, 1.0% achieved complete remission and 84.6% showed improvement (151). When combined with medication, acupoint stimulation may address somatic and stress–related symptoms not fully covered by pharmacotherapy alone, thereby enhancing overall efficacy. Pharmacotherapy subgroup analyses also identified ADs_HRT and ADs_TCM as superior options. Thus, for patients with moderate–to–severe symptoms, combinations of medication with acupoint stimulation, antidepressants with HRT, or antidepressants with TCM may serve as preferred first–line strategies.

drug_AcuStim also demonstrated a pronounced effect on endocrine regulation, ranking first in reducing LH levels (SUCRA = 100%) and significantly outperforming drug, control, and AcuStim, with a sufficient number of studies and low heterogeneity (I² = 13%). Mechanistically, animal studies suggest that acupuncture may exert antidepressant effects in part by downregulating menopause–related elevations in FSH and LH, thereby modulating central neurotransmitter activity (152). These findings support drug_AcuStim as an effective therapeutic option for perimenopausal patients requiring endocrine marker modulation.

All interventions demonstrated generally acceptable safety profiles. Although antipsychotics and certain antidepressants are associated with well–documented metabolic and neurological side effects—including weight gain, type 2 diabetes, dyslipidemia, tachycardia, and parkinsonian symptoms (153, 154)—the broad–category AE analysis revealed no significant differences among interventions (all 95% CrIs included 1), and combination therapy was not associated with an increased AE risk. Subgroup analyses further identified ADs_TCM as having the highest safety ranking (SUCRA = 82%). Collectively, these findings suggest that clinicians need not be deterred from considering combination regimens on safety grounds.

Although drug_psych and AcuStim_psych demonstrated advantages in improving depressive and anxiety symptoms, the supporting evidence base remains limited. Specifically, drug_psych ranked highest in SUCRA for HAMD reduction (92.4%), supported by a non–significant global inconsistency test (P = 0.053), minimal heterogeneity (I² = 0.3%), and favorable league table estimates. However, this finding rests on only 5 studies, which limits the robustness of the conclusion. Similarly, AcuStim_psych ranked highest in SUCRA for HAMA reduction (93.7%), with no significant global inconsistency (P = 0.343, I² = 4%), yet it is supported by only 3 studies, similarly constraining the strength of the evidence. Despite these limitations, both combination regimens showed a consistent trend toward superior efficacy. Accordingly, for patients with moderate–to–severe depression, combined medication and psychotherapy may be considered when available; for anxiety–predominant patients who prefer non–drug options, acupuncture plus psychotherapy is an alternative, though patients should be advised that the evidence is preliminary.

It is noteworthy that drug_psych ranked first in SUCRA across all five outcomes (KI, SDS, PSQI, FSH, and E2). However, each outcome was supported by only 2–5 studies, and several CrIs were relatively wide, limiting the precision of the estimates. This consistent cross–outcome pattern suggests that pharmacotherapy–psychotherapy combination may exert broad modulatory effects across mood, sleep, endocrine function, and menopausal symptoms. Several lines of evidence support this mechanistic plausibility. Perimenopausal hormonal fluctuations and neurosteroid changes are thought to disrupt GABAergic balance, increasing susceptibility to depression (155, 156). Sleep quality declines with aging but appears to be further compromised during the menopausal transition—nearly 40% of women in the SWAN study reported sleep disturbances associated with menopausal status rather than age (157). HRT is well–established for managing vasomotor symptoms and osteoporosis, and may confer additional benefits for sleep and mood in selected patients (158). Antidepressants enhance serotonergic transmission and promote neuroplastic changes that facilitate emotional relearning (159), while CBT addresses maladaptive cognitive patterns to reduce emotional distress (15). Thus, combined therapy may produce synergistic effects—antidepressants addressing biological dysregulation and psychotherapy modifying cognitive patterns—consistent with NMA evidence that combination therapy outperforms monotherapy in major depression. Nonetheless, given the limited evidence base and the imprecision of effect estimates, these findings should be considered hypothesis–generating rather than confirmatory. Current evidence does not support strong clinical recommendations; future large–scale, head–to–head RCTs are needed to validate the efficacy of drug_psych across diverse outcomes.

Drug subgroup analyses revealed that ADs_TCM exhibited a distinctive profile, ranking first by SUCRA for HAMD reduction (87.2%) and safety (82%), and second for clinical efficacy (13.4%), with favorable global consistency and low heterogeneity. This regimen significantly outperformed TCA, HRT, and SSRI monotherapies, suggesting that combining antidepressants with TCM may be a preferred strategy balancing efficacy and safety. Mechanistically, bioactive TCM constituents—flavonoids, phenolics, glycosides, phenylpropanoids, and alkaloids (160)—may act via multi–target pathways, modulating neurotransmitters, reducing neuroinflammation, and regulating the HPO axis (152). A meta–analysis of 40 studies (n = 3549) confirmed that Chinese herbal medicine monotherapy was superior to placebo and comparable to conventional antidepressants, while combination therapy yielded better clinical outcomes and a lower incidence of side effects (161). Our finding that ADs_TCM ranked highest in safety (SUCRA = 82%) aligns with this evidence. In contrast, the HAMA drug subgroup network showed significant global inconsistency (P < 0.001). As subgroup analyses did not identify its source (p = 0.158), results from this network should be interpreted cautiously.

This NMA overcomes the limitations inherent in pairwise comparisons of single interventions and, for the first time, simultaneously evaluates the relative efficacy of pharmacotherapy, acupoint stimulation, psychotherapy, and their combinations in perimenopausal mood disorders, providing a more comprehensive evidence base for clinical decision–making than previously available. Our results align with earlier pairwise meta–analyses that support the efficacy of SSRIs, acupuncture, and CBT in this population; However, those studies were unable to compare across treatment categories or assess combination strategies, a gap that the present NMA directly addresses.

Although this study provides valuable evidence-based insights, several limitations should be acknowledged. Firstly, drug_psych ranked highly across multiple outcomes (broad coverage), yet each outcome was supported by only 2–5 studies; drug_AcuStim had the strongest evidence base for overall efficacy. Secondly, the source of inconsistency in the HAMA drug subgroup remains unclear—within–subgroup heterogeneity for ADs_AP and SSRI was extremely low (I² = 0%), but the between–subgroup difference was not significant (p = 0.158). Thirdly, 95%CrIs for several outcomes (KI, SDS, PSQI) were wide, limiting the precision of effect estimates. Fourthly, although panic disorder was included in the search strategy, no eligible RCTs on perimenopausal panic disorder were identified, representing a notable gap in the literature. Fifthly, most included Chinese studies were of low methodological quality, with many rated as “unclear” or “high risk” for randomization, deviations from intended interventions, and outcome measurement—potentially affecting evidence credibility and partly explaining HAMA subgroup inconsistency. Sixthly, in the primary analysis, due to limited study numbers within each subgroup, we retained broad categorizations for acupoint stimulation and psychotherapy, and performed pharmacotherapy subgroup analyses only for HAMA, HAMD, clinical efficacy, and AEs. Fine–grained subgroup analyses for the remaining outcomes were not feasible. This broad categorization may mask differential effects within specific intervention modalities. To address these gaps, future research should prioritize:①head–to–head comparisons between drug_psych and drug_AcuStim to determine which combination strategy is superior; ②head-to-head RCTs comparing ADs_TCM with SSRIs to assess whether TCM-augmented antidepressant therapy outperforms standard SSRI monotherapy; ③validation of the top ranking of ADs_AP in the HAMA drug subgroup, given its significant global inconsistency and unclear source; ④large–sample RCTs of drug_psych for KI, SDS, and PSQI; ⑤RCTs specifically targeting perimenopausal panic disorder to fill the current research gap;⑥improved methodological quality (randomization, allocation concealment, blinding) to reduce bias and strengthen evidence reliability; ⑦more fine-grained studies comparing specific acupuncture modalities (e.g., electroacupuncture vs. manual acupuncture) and psychotherapeutic approaches (e.g., CBT vs. mindfulness).

## 5 Conclusion

Our NMA demonstrated efficacy advantages of pharmacotherapy, acupuncture, psychotherapy, and their combinations in perimenopausal women with anxiety and depressive disorders. Specifically, drug_AcuStim emerged as the most reliably effective strategy for improving overall clinical efficacy and reducing LH levels. drug_psych ranked first on the SDS, KI, PSQI, FSH, and E2—covering depressive symptoms, menopausal symptoms, sleep quality, and endocrine regulation—though its evidence base remains limited. AcuStim_psych showed promise for anxiety reduction (HAMA), while drug_psych also ranked highest for depression (HAMD), though both were constrained by small study numbers. In pharmacotherapy subgroup analyses, ADs_TCM exhibited a distinctive profile, combining top–ranked efficacy for depression (HAMD) and clinical efficacy with the highest safety ranking (AEs). Notably, none of the interventions were associated with an increased risk of AEs.

## Data Availability

The original contributions presented in the study are included in the article/**Supplementary Material**. Further inquiries can be directed to the corresponding author.

## References

[1] Duralde ER Sobel TH Manson JE . Management of perimenopausal and menopausal symptoms. Bmj. (2023) 382:e072612. doi: 10.1136/bmj-2022-072612 37553173

[2] Schmidt PJ . Depression, the perimenopause, and estrogen therapy. Ann N Y Acad Sci. (2005) 1052:27–40. doi: 10.1196/annals.1347.003 16024748

[3] Lin S Wang H Qiu J Li M Gao E Wu X . Altered gut microbiota profile in patients with perimenopausal panic disorder. Front Psychiatry. (2023) 14:1139992. doi: 10.3389/fpsyt.2023.1139992 37304433 PMC10249373

[4] Guan X Cao P . Brain mechanisms underlying panic attack and panic disorder. Neurosci Bull. (2024) 40:795–814. doi: 10.1007/s12264-023-01088-9 37477800 PMC11178723

[5] Kulkarni J Mu E Li Q Malicka M Gavrilidis E de Castella A . Bazedoxifene plus conjugated estrogen to treat menopausal depression—a pilot study. J Pharmacol Exp Ther. (2025) 392(4):103527. doi: 10.1016/j.jpet.2025.103527 40139074

[6] Kornstein SG Clayton AH Bao WH Guico-Pabia CJ . A pooled analysis of the efficacy of desvenlafaxine for the treatment of major depressive disorder in perimenopausal and postmenopausal women. J Womens Health. (2015) 24:281–90. doi: 10.1089/jwh.2014.4900 25860107

[7] Kim J Munster PN . Estrogens and breast cancer. Ann Oncol. (2025) 36:134–48. doi: 10.1016/j.annonc.2024.10.824 39522613 PMC12168202

[8] Xia W Khalil RA . Hormone replacement therapy and cardiovascular health in postmenopausal women. Int J Mol Sci. (2025) 26(11):5078. doi: 10.3390/ijms26115078 40507889 PMC12154064

[9] Carvalho AF Sharma MS Brunoni AR Vieta E Fava GA . The safety, tolerability and risks associated with the use of newer generation antidepressant drugs: a critical review of the literature. Psychother Psychosom. (2016) 85:270–88. doi: 10.1159/000447034 27508501

[10] Lin JG Kotha P Chen YH . Understandings of acupuncture application and mechanisms. Am J Transl Res. (2022) 14:1469–81. doi: 10.1016/j.jpain.2004.02.322 35422904 PMC8991130

[11] Cui L Li S Wang S Wu X Liu Y Yu W . Major depressive disorder: hypothesis, mechanism, prevention and treatment. Signal Transduct Target Ther. (2024) 9:30. doi: 10.1038/s41392-024-01738-y 38331979 PMC10853571

[12] Kim TH Lee MS Lee H . Sham acupuncture is not just a placebo. J Acupunct Meridian Stud. (2022) 15:333–5. doi: 10.51507/j.jams.2022.15.6.333 36537115

[13] Kim JH Yu HJ . The effectiveness of cognitive behavioral therapy on depression and sleep problems for climacteric women: a systematic review and meta-analysis. J Clin Med. (2024) 13(2):412. doi: 10.3390/jcm13020412 38256545 PMC10816049

[14] Ali AM Al-Dossary SA Laranjeira C Amer F Hallit S Alkhamees AA . Effects of hormonal replacement therapy and mindfulness-based stress reduction on climacteric symptoms following risk-reducing salpingo-oophorectomy. Healthcare (Basel). (2024) 12(16):1612. doi: 10.3390/healthcare12161612 39201170 PMC11353799

[15] Harlow SD Gass M Hall JE Lobo R Maki P Rebar RW . Executive summary of the stages of reproductive aging workshop + 10: addressing the unfinished agenda of staging reproductive aging. J Clin Endocrinol Metab. (2012) 97:1159–68. doi: 10.1210/jc.2011-3362 22344196 PMC3319184

[16] World Health Organization . ICD-10: International Statistical Classification of Diseases and Related Health Problems: Tenth Revision. Geneva, Switzerland: World Health Organization; (2004).

[17] American Academy of Family Physicians . Diagnostic and Statistical Manual of Mental Disorders, Fourth Edition: Primary Care Version. Washington, DC: American Psychiatric Association; (1995).

[18] American Psychiatric Association . Diagnostic and Statistical Manual of Mental Disorders, Fifth Edition, Text Revision (DSM-5-TR). Washington, DC: American Psychiatric Association Publishing; (2022).

[19] Association PBOTCM . Chinese Classification and Diagnostic Criteria for Mental Disorders. Jinan: Shandong Science and Technology Press (2001). p. 344.

[20] Sterne JAC Savović J Page MJ Elbers RG Blencowe NS Boutron I . RoB 2: a revised tool for assessing risk of bias in randomised trials. Bmj. (2019) 366:l4898. doi: 10.1136/bmj.l4898 31462531

[21] Bai Y Yang F . Clinical effect of acupuncture at siguan with moxibustion baihui on the treatment of 20 perimenopausal depression patients. Yunnan J Traditional Chin Med Mater Med. (2016) 37:45–6. doi: 10.16254/j.cnki.53-1120/r.2016.04.024

[22] Bi F Yan Y Ma J . Clinical efficacy of acupuncture treatment for menopausal syndrome with deficiency of kidney yin with anxiety disorder based on brain-gut axis theory. Tianjin J Traditional Chin Med. (2024) 41(6):754–8.

[23] Cai Y Zhang X Yang W Wang G Li J Luan X . Effects of ningshen mixture combined with "yishen tiaoqi" acupuncture on neuroendocrine,immune response and miRNA expression in peripheral blood of climacteric anxiety patients. J Liaoning Univ Traditional Chin Med. (2022) 24:191–5. doi: 10.13194/j.issn.1673-842x.2022.08.039

[24] Che J Xu L . The effects of the kidney-tonifying, depression-relieving, and heart-clearing decoction combined with acupuncture on estrogen levels and sleep quality in patients with menopausal depression. Med Equip. (2020) 33:84–6. doi: 10.3969/j.issn.1002-2376.2020.12.052

[25] Shen F Jiang Y . A study on the efficacy of the "liver-soothing, spleen-strengthening, and depression-relieving decoction" combined with acupuncture in the treatment of perimenopausal depression. Inner Mongolia J Traditional Chin Med. (2025) 44:42–3. doi: 10.16040/j.cnki.cn15-1101.2025.02.018

[26] Shen H Zhang J Zhang W Yang N . Clinical observation on treating female menopausal anxiety disorder with acupuncture combined with group psychotherapy. China J Traditional Chin Med. (2016) 31:3829–31.

[27] Shen J Dong L Huang H . Effect of bushen-jieyu-qingxin decoction combined with acupuncture on ACT-INH-FS pathway of women with perimenopausal depression. Modern J Integr Traditional Chin Western Med. (2017) 26:2512–4+24. doi: 10.3969/j.issn.1008-8849.2017.23.002

[28] Chen H Yao Z Lu J Zhao L Zhu L . Clinical study on influence of Chinese and Western medicine on monoamine neurotransmitter of depression patients during peri-menopausal period. Chin Arch Traditional Chin. (2012) 30(10):2267–9. doi: 10.13193/j.archtcm.2012.10.125.chenh.069

[29] Chen X Xie S He W Wu S . Clinical observation of prozac with nilestriol in the treatment of peri-menopausal women with depression. J Shantou Univ Med Coll. (2007) 20:103–4. doi: 10.3969/j.issn.1007-4716.2007.02.014

[30] Chen Y . A study on the clinical efficacy of Chai-Shao-Yu-Huan decoction as an adjuvant to acupuncture in the treatment of menopausal depression. Chin Sci Technol J Database (Full-Text Edition): Med Health. (2023)(12):74–6.

[31] Chen Z Zhou S . Treatment of 30 cases of female menopausal depression with acupuncture. J Shandong Univ Traditional Chin Med. (2010) 34:44–5. doi: 10.16294/j.cnki.1007-659x.2010.01.018

[32] Chen X Yu J Xi M Huang Y . An observation on the efficacy of citalopram hydrobromide in treating perimenopausal depression. J Front Med. (2021) 11:27–8.

[33] Chi H Zou W . Yishen tiaoan acupuncture therapy in the treatment of perimenopausal depression: an observation of 30 cases. J Clin Acupuncture Moxibustion. (2011) 27:4–7. doi: 10.3969/j.issn.1005-0779.2011.07.002

[34] Dai W Liao M Dai L . Clinical effect of electroacupuncture combined with escitalopram oxalate for perimenopausal depression. Hebei J Traditional Chin Med. (2022) 44:1177–80. doi: 10.3969/j.issn.1002-2619.2022.07.027

[35] Ding L Liu B . Clinical observation on perimenopausal depression by the acupuncture treatment of nourishing——kidney regulating-liver strengthening-spleen and tranquilizing-heart. Chin Arch Traditional Chin Med. (2007) 25:1066–7. doi: 10.13193/j.archtcm.2007.05.203.dingl.095

[36] Ding X . The effectiveness of group psychotherapy as an adjunctive treatment for perimenopausal anxiety in women. Women's Health Res. (2023), 66–7,87.

[37] Dong C Xu Y Jin Y Yu K Deng Y . Clinical study on brain-gut co-treatment acupuncture combined with zuogui decoction plus xiaoyao powder in treating perimenopausal depression of kidney deficiency and liver stagnation type. New Chin Med. (2025) 57:88–93. doi: 10.13457/j.cnki.jncm.2025.20.016

[38] Dong C Gao Y Li Y Wu L . Clinical study on sertraline combined with acupuncture and moxibustion for perimenopausal depression. New Chin Med. (2021) 53:129–33. doi: 10.13457/j.cnki.jncm.2021.14.034

[39] Dong Y . Clinical observation of acupuncture of beishu point in treatment of climacteric depression. Jilin J Chin Med. (2015) 35:306–8. doi: 10.13463/j.cnki.jlzyy.2015.03.028

[40] Fan Y . Clinical efficacy of liver-soothing and depression-relieving capsules as an adjuvant treatment for menopausal hypertension with anxiety in women. Women's Health Res. (2024), 47–9.

[41] Fu J Wen L . An observation on the efficacy of cognitive therapy combined with fluvoxamine maleate in treating perimenopausal depression and anxiety. J Qiqihar Med Univ. (2011) 32:3853–4. doi: 10.3969/j.issn.1002-1256.2011.23.049

[42] Fu R Wang H Yu L . A clinical study on the combined use of jiawei xiaoyao san and dihydroxyethyldihydrochloride for the treatment of menopausal anxiety. Asia-Pacific Traditional Med. (2018) 14:170–1.

[43] Gao J Wang X Zhang W Gu J Weng X Yu G . The application effect of ear acupuncture combined with baduanj in on depression in perimenopausal women. J Clin Res. (2023) 40:816–9. doi: 10.3969/j.issn.1671-7171.2023.06.005

[44] Gao Y Zhou C Yan H . The effectiveness of psychological intervention combined with health education in improving menopausal syndrome and anxiety in women. Chin J Modern Nurs. (2011) 17:4323–5. doi: 10.3760/cma.j.issn.1674-2907.2011.35.029

[45] Gu T Wang R Wu T Ke Z Yang H Wang D . Therapeutic effect on mild perimenopausal depression treated with acupuncture at the "thirteen ghost points" and kaixin powder. Chin Acupuncture Moxibustion. (2020) 40:267–71. doi: 10.13703/j.0255-2930.20190308-0005 32270639

[46] Guo Y Liu C . An observation on the efficacy of electroacupuncture combined with low-dose amitriptyline in the treatment of menopausal depression. Guangming J Chin Med. (2005) 20:32–3. doi: 10.3969/j.issn.1003-8914.2005.02.026

[47] Huang H Shen J Dong Y Dong L . Effect of acupuncture plus medication on HAMD, SDS and H-P-O axis in perimenopausal depression patients. Shanghai J Acupuncture Moxibustion. (2017) 36:705–10. doi: 10.13460/j.issn.1005-0957.2017.06.0705

[48] Huo J Yu H Kong D Fu H . A comparative study of traditional Chinese medicine and Western medicine treatments for menopausal anxiety in women. J Med Forum. (2008) 29:61–2. doi: 10.3969/j.issn.1672-3422.2008.12.036

[49] Ji L . The effects of escitalopram oxalate and cognitive behavioral therapy on the psychological status of women with menopausal depression in high-altitude regions. J High Altitude Med. (2021) 31:23–7. doi: 10.3969/j.issn.1007-3809.2021.04.004

[50] Jiang X Ren K Zhao X . Comparison of escitalopram and paroxetine for patient with first-episode climacteric depression. China Med. (2009) 4:282–3. doi: 10.3760/cma.j.issn.1673-4777.2009.04.019 30704229

[51] Xie Y Hong C Zhao W . Clinical observation of 30 cases of menopausal depression treated with acupuncture combined with traditional Chinese medicine. Chin J Traditional Med Sci. (2013) 20:293–4. doi: 10.3969/j.issn.1005-7072.2013.03.061

[52] Jin Y Xiang H Zheng L . Influence of catgut implantation at back-shu points on HAMD score and sex hormones in patients with perimenopausal depression and anxiety. Chin Arch Traditional Chin Med. (2013) 31:1322–4. doi: 10.13193/j.archtcm.2013.06.108.jinyb.021

[53] Lai A Zhao Y Qi H Zhang J Zhang L Weng Y . Comparison of different antidepression therapy in perimenopausal and postmenopausal women with depression. Chin J Obstet Gynecology. (2007) 42:169–72. doi: 10.3760/j.issn:0529-567x.2007.03.007 17537301

[54] Li J Liu D Chu X Gong Y Xia X . Treatment of menopausal depression with Chaishao Yuhuan decoction combined with Kaiyu acupuncture. J Pract Med. (2022) 38(15):1960–4.

[55] Li L Yang Y Yin G . Clinical observation on the treatment of menopausal anxiety using a combination of auricular pressure, acupuncture, and Dailixin. New Chin Med. (2013) 45:124–6. doi: 10.13457/j.cnki.jncm.2013.05.031

[56] Li N . Clinical research of Zishen-Shugan-Ningxin prescription to treat female menopausal depression. Chin J Exp Traditional Med Formulae. (2012) 18:293–6. doi: 10.13422/j.cnki.syfjx.2012.20.020

[57] Li P Dai W . Clinical observation of electro-acupuncture in the treatment of menopause depression. Clin J Traditional Chin Med. (2020) 32:555–8. doi: 10.16448/j.cjtcm.2020.0344

[58] Li Q Zhou L Dong Z Yin R . Effect of hormone replacement therapy (HRT) on depression in perimenopausal women. Sichuan Med J. (2017) 38:338–40. doi: 10.16252/j.cnki.issn1004-0501-2017.03.026

[59] Li Y Gu L Wang C Liang Y . Observation on the efficacy of liver-soothing and mind-regulating electroacupuncture combined with western medicine in treating menopausal depression in women. J Guangzhou Univ Traditional Chin Med. (2026) 43:674–80. doi: 10.13359/j.cnki.gzxbtcm.2026.03.018

[60] Li Z Huang L Chen Y . Effect of hormone replacement therapy on depression patients during perimenopausal. J Int Psychiatry. (2021) 48:105–107+14. doi: 10.13479/j.cnki.jip.2021.01.031

[61] Ziqing Chai C Fu W Luo D . Clinical observation on the Shugan Tiaoshen acupuncture in the treatment of mild-to-moderate perimenopausal depressive disorder. J Guangzhou Univ Traditional Chin Med. (2024) 41:1226–32. doi: 10.13359/j.cnki.gzxbtcm.2024.05.020

[62] Liu H Chen G . The effects of acupuncture combined with sertraline hydrochloride on hormone levels and neurotransmitters in women with menopausal depression: a report on 40 clinical cases. Jiangsu J Traditional Chin Med. (2019) 51:70–2. doi: 10.3969/j.issn.1672-397X.2019.02.027

[63] Liu J Pan A Zhu C Tang W Shi S . The effectiveness of integrated traditional Chinese and western medicine therapy in treating depression comorbid with type 2 diabetes in perimenopausal women and its impact on ovarian function. Maternal Child Health Care China. (2023) 38:1521–4. doi: 10.19829/j.zgfybj.issn.1001-4411.2023.08.042

[64] Liu R . A controlled study on the treatment of menopausal depression using a combination of traditional Chinese and western medicine. Beijing J Traditional Chin Med. (2007) 26:512–3. doi: 10.3969/j.issn.1674-1307.2007.08.020

[65] Liu X Wang S Song Q Chen L . Effect of Huanglian Wendan decoction combined with Jiejie acupuncture on perimenopausal depression syndrome of phlegm-heat internal disturbance. World J Complex Med. (2023) 9:27–30.

[66] Liu Y . A comparison of the efficacy of hormone replacement therapy and antidepressants in alleviating menopausal symptoms and mood disorders. Chin J Primary Med Pharm. (2013) 20:277–9. doi: 10.3760/cma.j.issn.1008-6706.2013.02.056 30704229

[67] Lou L . The application of cognitive behavioral therapy in women with menopausal depression. J Qilu Nurs. (2012) 18:107–8. doi: 10.3969/j.issn.1006-7256.2012.10.069

[68] Lu X Zou F Peng H Wu H Qiu J . Efficacy of estrogen progesterone replacement therapy combined with alprazolam tablets in the treatment of perimenopausal syndrome with anxiety disorder. Med Innovation China. (2022) 19:134–8. doi: 10.3969/j.issn.1674-4985.2022.36.032

[69] Lv M Chen Q . The efficacy of combining acupuncture and medication in treating perimenopausal depression. Med J Chin People's Health. (2019) 31:115–6. doi: 10.3969/j.issn.1672-0369.2019.11.051

[70] Lv Y . A study on the efficacy of fluoxetine combined with estradiol valerate in the treatment of perimenopausal depression. Shaanxi Med J. (2009) 38:1667–9. doi: 10.3969/j.issn.1000-7377.2009.12.041

[71] Luo Y . A study on the efficacy of simplified cognitive behavioral therapy for inpatients with perimenopausal anxiety disorder. Electron J Pract Gynecologic Endocrinol. (2020) 7:37–8. doi: 10.16484/j.cnki.issn2095-8803.2020.21.026

[72] Ma J Liu Z . A clinical study of the acupuncture treatment in the treatment of perimenopausal depression. J Psychiatry. (2009) 22:276–8. doi: 10.3969/j.issn.1009-7201.2009.04.013

[73] Ma Y Guo Y Li X . Intelligent cupping therapy with paroxetine for female menopausal depression. Med J Chin People's Health. (2011) 23(11):1426–8.

[74] Men S . Effects of electroacupuncture combined with drugs on sleep quality and neuroendocrine in patients with perimenopausal depression. Med J Chin People's Health. (2022) 34:90–2. doi: 10.3969/j.issn.1672-0369.2022.07.028

[75] Ning Y . A clinical study on acupuncture treatment for menopausal depression in women. J Sichuan Traditional Chin Med. (2015) 33:154–5.

[76] Niu X Wang P . Clinical study on acupuncture Wangshi Wuzangshu combined Geshu in the treatment of liver qi stagnation type of female climacteric depression. Int J Traditional Chin Med. (2017) 39:999–1002. doi: 10.3760/cma.j.issn.1673-4246.2017.11.010 30704229

[77] Pan Z . A comparative study of fluvoxamine, melitracen, and paroxetine in the treatment of perimenopausal depression. China Health Care Nutr. (2019) 29:69. doi: 10.1002/9781118541203.xen391 41531421

[78] Peng B Zhang W Zhang R Tang W Cheng W . Comparison of antidepressive drugs and hormone replacement therapy in perimenopausal and postmenopausal women with depression. Chin J Psychiatry. (2012) 45:141–4. doi: 10.3760/cma.j.issn.1006-7884.2012.03.004 30704229

[79] Qian J Zhang J Pei Y Chen J . Clinical observation on treatment of involutional depression with the therapy of Wang's Wu-zang-shu and Ge-shu. Beijing J Traditional Chin Med. (2007) 26:491–2. doi: 10.16025/j.1674-1307.2007.08.011

[80] Qian J Zhang B Liu Rui J Lin Z Jiang M . An analysis of the efficacy of different treatment methods for depression in perimenopausal and postmenopausal women. World Latest Med Inf. (2015) 15:63. doi: 10.3969/j.issn.1671-3141.2015.81.046

[81] Qiang B . Acupuncture treatment for menopausal depression in 30 women. Shaanxi J Traditional Chin Med. (2008) 29:871–2. doi: 10.3969/j.issn.1000-7369.2008.07.090

[82] Qin E Guo Y Li Y . A clinical study on acupoint thread implantation for the treatment of mild to moderate depression during the perimenopausal period. Liaoning J Traditional Chin Med. (2019) 46:1721–4. doi: 10.13192/j.issn.1000-1719.2019.08.046

[83] Qin L Liu W . Clinical observation of Longsha opening-closing six climate factors needling therapy combined with modified Huanglian Wendan decoction in the treatment of perimenopausal depression with internal disturbance of phlegm-heat syndrome type. J Guangzhou Univ Traditional Chin Med. (2022) 39:1306–13. doi: 10.13359/j.cnki.gzxbtcm.2022.06.015

[84] Qiu L Jiang H . Influence of acupuncture and psychological counseling on emotional depression of the patients during perimenopausal period. Western J Traditional Chin Med. (2014) 27:131–3. doi: 10.3969/j.issn.1004-6852.2014.11.045

[85] Shi Huang D Feng Q Tang N . Clinical observation on electroacupuncture treatment for mild to moderate depression in peri-menopausal period. China J Modern Med. (2018) 28:52–6. doi: 10.3969/j.issn.1005-8982.2018.20.009

[86] Shi X Yang S Wang Y Zhang G He J . Clinical analysis on the curative effects of electroacupuncture, herbal medicine and electroacupuncture plus herbal medicine in treatment of perimenopausal depression. Maternal Child Health Care China. (2011) 26:5364–6.

[87] Song G Wang H Yang J Jiang B He Y Tian Y . The efficacy of serotonin reuptake inhibitors combined with electroacupuncture for perimenopausal anxiety and depression disorders. J Ningxia Med Univ. (2022) 44:1240–4. doi: 10.16050/j.cnki.issn1674-6309.2022.12.011

[88] Sun Y Qin Z Jiang C Su S . Clinical observations on electroacupuncture treatment for mild to moderate depressive disorders in perimenopausal women. J Guangxi Univ Chin Med. (2015) 18:13–5.

[89] Sun Z Jin Y Xiang H Liu F . Clinical observation on mild perimenopausal depression of kidney deficiency and liver stagnation syndrome treated with acupoint catgut implantation. Chin Acupuncture Moxibustion. (2015) 35:443–6. doi: 10.13703/j.0255-2930.2015.05.009 26255514

[90] Tan J Yang L . Treatment of perimenopausal depression in 62 cases using a combination of tortoise shell and curcuma decoction and acupuncture. Shaanxi J Traditional Chin Med. (2010) 31:812–3. doi: 10.3969/j.issn.1000-7369.2010.07.031

[91] Tang N Shi J Huang D . Influences of electroacupuncture combined with xiaoyaosan on effect and serum acth, cort in perimenopausal depression patients. J Guangxi University(Nat Sci Ed). (2019) 44:587–92. doi: 10.13624/j.cnki.issn.1001-7445.2019.0587

[92] Wang F Chen Z . A clinical observation on the treatment of first-episode depression during menopause with the combination of citalopram and quetiapine. J Qiqihar Med Univ. (2011) 32:1056–7. doi: 10.3969/j.issn.1002-1256.2011.07.020

[93] Wang J Zhang Y Tang J . Clinical study of agomelatine to treat insomnia in perimenopausal patients with anxiety and depression. J Xuzhou Med Univ. (2020) 40:344–6. doi: 10.3969/j.issn.2096-3882.2020.05.007

[94] Wang K Xie X Shen Y Hu Q Xie G Zou Q . A study on the efficacy and mechanism of action of Jiuwei Zhenxin granules in the treatment of menopausal anxiety. J Med Inf. (2016) 29:250. doi: 10.3969/j.issn.1006-1959.2016.20.192

[95] Wang S . Integrated traditional Chinese and western medicine for the treatment of perimenopausal depression in women. Women's Health Res. (2015), 51.

[96] Wang W Yue L Du M Gao Y Wang Q Xi S . Comparison of Jiawei Xiaoyao pills in treating women with emotional disorder during perimenopause. China J Traditional Chin Med Pharm. (2014) 29(3):836–9.

[97] Wang X Li X Deng A Bo Z . Comparative study on abdominal acupuncture and western medicine for treatment of menopause depressive disorder. Chin Acupuncture Moxibustion. (2010) 30:913–7. doi: 10.13703/j.0255-2930.2010.11.026 21246847

[98] Wu J Deng Y Liao J . Influence of timing five-tone therapy combined with acupoint application based on midnight-noon ebb-flow theory on climacterium depression. Med Innovation China. (2023) 20:112–6. doi: 10.3969/j.issn.1674-4985.2023.18.026

[99] Wu Y Jin C Chen F Sang B Zhao G Wang J . Clinical observation of electro-acupuncture combined with Shugan Jieyu capsule in treatment of perimenopausal depression (liver depression type). Inf Traditional Chin Med. (2022) 39:51–4. doi: 10.19656/j.cnki.1002-2406.20220110

[100] Xia S Yu H . An observation on the efficacy of duloxetine in treating depression in perimenopausal and postmenopausal women an observation on the efficacy of duloxetine. Prog Obstet Gynecology. (2014) 23:503–4. doi: 10.13283/j.cnki.xdfckjz.2014.06.026

[101] Xie S Chen X Wu S . An observation on the efficacy of estrogen combined with antidepressants in treating menopausal (syndrome) depression in women. J Chin Physician. (2005), 332–3.

[102] Xing K . Clinical observation of 120 cases of menopausal depression in women treated with the “mind-awaken and depression-relieve” acupuncture method. Maternal Child Health Care China. (2011) 26:5373–5.

[103] Xin X Guo Q Fu S Han M Deng Y . Clinical observations on the effects of Toupi Hua on sleep disorders in perimenopausal patients with anxiety. Health Wellness Mag. (2021) 22(19):199–200.

[104] Yang X Xiong Y . A clinical observation on the treatment of first-episode depression during menopause with citalopram combined with quetiapine. Guide China Med. (2012) 10:195–7. doi: 10.3969/j.issn.1671-8194.2012.01.157

[105] Yu X Gao C Ren Y Li X Sheng Q Li F . Effect of estrogen on depressive disorder of perimenopausal and postmenopausal women. Chin J Woman Child Health. (2007) 18:371–3. doi: 10.3969/j.issn.1673-5293.2007.05.007

[106] Yu Y Liu K Li Y Wang D Men J Cong H . Chaihu shugan powder combined with shu-mu points compatibility in treatment of perimenopausal depression. Inf Traditional Chin Med. (2023) 40:57–62. doi: 10.19656/j.cnki.1002-2406.20230409

[107] Yuan Z . A study on the clinical efficacy of integrated traditional chinese and western medicine in the treatment of menopausal depression. Int Med Health Guid. (2015) 21:2590–1. doi: 10.3760/cma.j.issn.1007-1245.2015.17.030 30704229

[108] Zhang C Xu S . Integrated traditional Chinese and Western medicine treatment for 40 cases of perimenopausal depression in women. Henan Traditional Chin Med. (2013) 33(9):1534–5.

[109] Zhang C Yao W . Clinical efficacy evaluation of paroxetine combined with wuling capsules in the treatment of perimenopausal depression in women. Seek Med And Ask Med. (2011) 9:545–6.

[110] Zhang D Zhang R Yang Y . The efficacy of modified chaihu jia longgu muli decoction combined with acupoint implantation at the five zang organ shu points in the treatment of menopausal depression of the liver qi stagnation type. Doctor. (2025) 10:53–7. doi: 10.3969/j.issn.2096-2665.2025.24.014

[111] Zhang D . Clinical observation of bushen jieyu qingxin decoction combined with bushen tiaogan jianpi ningxin acupuncture on patients with perimenopausal depression. Capital Food Med. (2021) 28:159–60. doi: 10.3969/j.issn.1005-8257.2021.18.077

[112] Zhang X Su S Qin M Cai H Huang M Dai Q . Therapeutic effect of acupuncture combined with wheat-grain moxibustion on perimenopausal depression with kidney deficiency and liver depression. Chin Acupuncture Moxibustion. (2021) 41:377–80. doi: 10.13703/j.0255-2930.20200401-k0002 33909356

[113] Zhang Y Xu S Li J . Observation on the regulation effect of acupuncture therapy combined with western medical treatment on clinical symptoms and sex hormone and neuroendocrine in patients with menopausal anxiety disorder. J Med Pharm Chin Minorities. (2025) 31:38–41. doi: 10.16041/j.cnki.cn15-1175.2025.12.015

[114] Zhao B Zhao R Peng X Zhang G Li X . Treatment of perimenopausal depression in 43 cases using a proprietary decoction for soothing the liver, strengthening the spleen, and relieving depression combined with psychological intervention. China Pharm. (2015) 24:232–3.

[115] Zheng C . Combined treatment for menopausal depression in women. Chin Community Doctors. (2013) 15:18. doi: 10.3969/j.issn.1007-614x.2013.08.012

[116] Zheng G Huang R Guo S . Clinical observation on the treatment of perimenopausal depression with thermosensitive moxibustion. J Pract Traditional Chin Med. (2016) 32:1213–4. doi: 10.3969/j.issn.1004-2814.2016.12.055

[117] Zheng K Chen W . The rehabilitation therapy of with depressive symptom of women in life of menopause. Maternal Child Health Care China. (2007) 22(27):3856–8. doi: 10.3969/j.issn.1001-4411.2007.27.045

[118] Zheng L Liang X Tian Y Lu S Cheng A . The effects of acupuncture on reproductive hormone levels in patients with perimenopausal depression. J Yunnan Univ Chin. (2018) 41:76–8. doi: 10.19288/j.cnki.issn.1000-2723.2018.04.019

[119] Zheng S Wu Y Liao J Xu M Hu Z Chen L . A clinical study on the treatment of perimenopausal depression using the “four divine needles” long-retention acupuncture method. Liaoning J Traditional Chin Med. (2010) 37:726–8. doi: 10.13192/j.ljtcm.2010.04.155.zhengshh.040

[120] Zhong Y Luo J Kuang X Shen Y . Chinese medicine acupoint application combined with thought field therapy in the treatment of perimenopausal depression based on the theory of mutual supplementation between the kidney and the brain. Chin Med Modern Distance Educ China. (2025) 23(12):118–21.

[121] Zhou Q Wang J Cui X . Clinical observation on acupuncture for perimeno pausal depression penmenopausal depression. J Acupuncture Tuina Sci. (2009) 7:200–2.

[122] Zhou Y . Clinical observation on shugan jianpi jieyu decoction combined with acupuncture in the treatment of perimenopausal depression. Chin Med Modern Distance Educ China. (2022) 20:58–60. doi: 10.3969/j.issn.1672-2779.2022.16.022

[123] Zhu X Chen X Chai B Chang L . Paroxetine combined with low dose of olanzapine in the treatment of peri-menopausal depression. Aviat Med Air Force. (2020) 36:433–6. doi: 10.3969/j.issn.2095-3402.2020.05.019

[124] Zou Y . The effects of psychological intervention on the psychological and behavioral patterns of perimenopausal women. Health Way. (2013) 12:25–6. doi: 10.3969/j.issn.1671-8801.2013.10.022

[125] Berlanga C Mendieta D Alva G Lara MD . Failure of tibolone to potentiate the pharmacological effect of fluoxetine in postmenopausal major depression. J Womens Health Gender-Based Med. (2003) 12:33–9. doi: 10.1089/154099903321154121 12639367

[126] Blumenthal JA Babyak MA Moore KA Craighead WE Herman S Khatri P . Effects of exercise training on older patients with major depression. Arch Intern Med. (1999) 159:2349–56. doi: 10.1001/archinte.159.19.2349 10547175

[127] Cao XJ Huang XC Wang XY . Effectiveness of chinese herbal medicine granules and traditional chinese medicine-based psychotherapy for perimenopausal depression in chinese women: a randomized controlled trial. Menopause-the J Menopause Soc. (2019) 26:1193–203. doi: 10.1097/gme.0000000000001380 31513088

[128] De Novaes Soares C Almeida OP Joffe H Cohen LS . Efficacy of estradiol for the treatment of depressive disorders in perimenopausal women: a double-blind, randomized, placebo-controlled trial. Arch Gen Psychiatry. (2001) 58:529–34. doi: 10.1001/archpsyc.58.6.529 11386980

[129] Joffe H Petrillo LF Koukopoulos A Viguera AC Hirschberg A Nonacs R . Increased estradiol and improved sleep, but not hot flashes, predict enhanced mood during the menopausal transition. J Clin Endocrinol Metab. (2011) 96:E1044–54. doi: 10.1210/jc.2010-2503 21525161 PMC3135198

[130] Kao CL Chen CH Lin WY Chiao YC Hsieh CL . Effect of auricular acupressure on peri- and early postmenopausal women with anxiety: a double-blinded, randomized, and controlled pilot study. Evid Based Complement Alternat Med. (2012) 2012:567639. doi: 10.1155/2012/567639 22649475 PMC3358095

[131] Khoshbooii R Hassan SA Deylami N Muhamad R Kamarudin EME Alareqe NA . Effects of group and individual culturally adapted cognitive behavioral therapy on depression and sexual satisfaction among perimenopausal women. Int J Environ Res Public Health. (2021) 18. doi: 10.3390/ijerph18147711 34300161 PMC8303550

[132] Koçak DY Varişoğlu Y . The effect of music therapy on menopausal symptoms and depression: a randomized-controlled study. Menopause. (2022) 29:545–52. doi: 10.1097/gme.0000000000001941 35486946

[133] Kornstein SG Jiang Q Reddy S Musgnung JJ Guico-Pabia CJ . Short-term efficacy and safety of desvenlafaxine in a randomized, placebo-controlled study of perimenopausal and postmenopausal women with major depressive disorder. J Clin Psychiatry. (2010) 71:1088–96. doi: 10.4088/JCP.10m06018blu 20797382

[134] Kulkarni J Gavrilidis E Thomas N Hudaib AR Worsley R Thew C . Tibolone improves depression in women through the menopause transition: a double-blind randomized controlled trial of adjunctive tibolone. J Affect Disord. (2018) 236:88–92. doi: 10.1016/j.jad.2018.04.103 29723767

[135] Li L Xu X Tao Y Zhang X . A clinical study on Chinese five-element music therapy combined with auricular-plaster therapy in treating perimenopausal insomnia and anxiety. Altern Ther Health Med. (2024):AT10182. 38870496

[136] Li S Li ZF Wu Q Guo XC Xu ZH Li XB . A multicenter, randomized, controlled trial of electroacupuncture for perimenopause women with mild-moderate depression. BioMed Res Int. (2018) 2018:5351210. doi: 10.1155/2018/5351210 30003102 PMC5996410

[137] Linde JA Simon GE Ludman EJ Ichikawa LE Operskalski BH Arterburn D . A randomized controlled trial of behavioral weight loss treatment versus combined weight loss/depression treatment among women with comorbid obesity and depression. Ann Behav Med. (2011) 41:119–30. doi: 10.1007/s12160-010-9232-2 20878292 PMC3033656

[138] Liu X Li M Xie X Li Y Li K Fan J . Efficacy of manual acupuncture vs. placebo acupuncture for generalized anxiety disorder (GAD) in perimenopausal women: a randomized, single-blinded controlled trial. Front Psychiatry. (2023) 14:1240489. doi: 10.3389/fpsyt.2023.1240489 37854443 PMC10579903

[139] Mehdipour A Abedi P Ansari S Dastoorpoor M . The effectiveness of emotional freedom techniques (EFT) on depression of postmenopausal women: a randomized controlled trial. J Complement Integr Med. (2022) 19:737–42. doi: 10.1515/jcim-2020-0245 34013673

[140] Rasgon NL Altshuler LL Fairbanks LA Dunkin JJ Davtyan C Elman S . Estrogen replacement therapy in the treatment of major depressive disorder in perimenopausal women. J Clin Psychiatry. (2002), 45–8. 11995778

[141] Schmidt PJ Ben Dor R Martinez PE Guerrieri GM Harsh VL Thompson K . Effects of estradiol withdrawal on mood in women with past perimenopausal depression: a randomized clinical trial. JAMA Psychiatry. (2015) 72:714–26. doi: 10.1001/jamapsychiatry.2015.0111 26018333 PMC6391160

[142] Schmidt PJ Wei SM Martinez PE Dor RRB Guerrieri GM Palladino PP . The short-term effects of estradiol, raloxifene, and a phytoestrogen in women with perimenopausal depression. Menopause. (2021) 28:369–83. doi: 10.1097/gme.0000000000001724 33470755 PMC9022873

[143] Tam LW Parry BL . Does estrogen enhance the antidepressant effects of fluoxetine? J Affect Disord. (2003) 77:87–92. doi: 10.1016/S0165-0327(02)00357-9 14550939

[144] Ushiroyama T Ikeda A Sakuma K Ueki M . Changes in serum tumor necrosis factor (TNF-α) with kami-shoyo-san administration in depressed climacteric patients. Am J Chin Med. (2004) 32:621–9. doi: 10.1142/S0192415X04002259 15481651

[145] Wang J Liao Y You Y Liang W Wan L Yang H . Acupuncture and chinese herbal medicine for menopausal mood disorder: a randomized controlled trial. Climacteric. (2023) 26:392–400. doi: 10.1080/13697137.2023.2187284 36921619

[146] Yang C Zhang X Bie J Kang W Sun G Zhao Q . Gut microbiota drives dietary lignans to improve perimenopausal depression via activating hippocampal ERβ/GluN2A/PSD95 pathway. Pharmacol Res. (2026) 227:108161. doi: 10.1016/j.phrs.2026.108161 41833765

[147] Zeng Y Huang X Chen C Nie G Cao X Wang J . A randomized, controlled clinical trial of combining therapy with traditional Chinese medicine-based psychotherapy and Chinese herbal medicine for menopausal women with moderate to serious mood disorder. Evidence-Based Complementary Altern Med. (2019) 2019:9581087. doi: 10.1155/2019/9581087 30723517 PMC6339727

[148] Zhao FY Zheng Z Fu QQ Conduit R Xu H Wang HR . Acupuncture for comorbid depression and insomnia in perimenopause: a feasibility patient-assessor-blinded, randomized, and sham-controlled clinical trial. Front Public Health. (2023) 11:1120567. doi: 10.3389/fpubh.2023.1120567 36815166 PMC9939459

[149] Zhou JH Zhang DL Ning BL Xue XJ Zhao L Wu Q . The role of acupuncture in hormonal shock-induced cognitive-related symptoms in perimenopausal depression: a randomized clinical controlled trial. Front Psychiatry. (2022) 12. doi: 10.3389/fpsyt.2021.772523 35095593 PMC8793332

[150] Bu Y Yan J Zhang Z Xue S Chi F Zheng Y . Acupuncture and the HPO axis: A review of neuroendocrine mechanisms with implications for ovarian function. J Integr Neurosci. (2025) 24:39451. doi: 10.31083/jin39451 41200977

[151] Williams RE Kalilani L DiBenedetti DB Zhou X Fehnel SE Clark RV . Healthcare seeking and treatment for menopausal symptoms in the United States. Maturitas. (2007) 58:348–58. doi: 10.1016/j.maturitas.2007.09.006 17964093

[152] Wang L Liu Y Qi X Luo H . Perimenopausal depression: Etiology, clinical characteristics, and the role of traditional Chinese medicine. Int J Psychiatry Clin Pract. (2025) 29:110–6. doi: 10.1080/13651501.2025.2501250 40515694

[153] De R Amin Alfatwa Y Kanagasundaram P Saragosa JE Chan J Remington G . The effect of semaglutide on antipsychotic-induced weight gain and other metabolic parameters, among a cohort of inpatients. Schizophr Bull. (2026) 52(3):sbaf236. doi: 10.1093/schbul/sbaf236 42297449 PMC13267902

[154] Khawam EA Laurencic G Malone DA Jr . Side effects of antidepressants: An overview. Cleve Clin J Med. (2006) 73:351–353, 6-61. doi: 10.3949/ccjm.73.4.351 16610395

[155] Gordon JL Girdler SS Meltzer-Brody SE Stika CS Thurston RC Clark CT . Ovarian hormone fluctuation, neurosteroids, and HPA axis dysregulation in perimenopausal depression: A novel heuristic model. Am J Psychiatry. (2015) 172:227–36. doi: 10.1176/appi.ajp.2014.14070918 25585035 PMC4513660

[156] Santoro N Roeca C Peters BA Neal-Perry G . The menopause transition: Signs, symptoms, and management options. J Clin Endocrinol Metab. (2021) 106:1–15. doi: 10.1210/clinem/dgaa764 33095879

[157] Kravitz HM Ganz PA Bromberger J Powell LH Sutton-Tyrrell K Meyer PM . Sleep difficulty in women at midlife: A community survey of sleep and the menopausal transition. Menopause. (2003) 10:19–28. doi: 10.1097/00042192-200310010-00005 12544673

[158] Polo-Kantola P Erkkola R Irjala K Pullinen S Virtanen I Polo O . Effect of short-term transdermal estrogen replacement therapy on sleep: A randomized, double-blind crossover trial in postmenopausal women. Fertil Steril. (1999) 71:873–80. doi: 10.1016/s0015-0282(99)00062-x 10231049

[159] Sharp T Collins H . Mechanisms of SSRI therapy and discontinuation. Curr Top Behav Neurosci. (2024) 66:21–47. doi: 10.1007/7854_2023_452 37955823

[160] Qu F Cai X Gu Y Zhou J Zhang R Burrows E . Chinese medicinal herbs in relieving perimenopausal depression: A randomized, controlled trial. J Altern Complement Med. (2009) 15:93–100. doi: 10.1089/acm.2008.0267 19769482

[161] Wang Y Shi YH Xu Z Fu H Zeng H Zheng GQ . Efficacy and safety of Chinese herbal medicine for depression: A systematic review and meta-analysis of randomized controlled trials. J Psychiatr Res. (2019) 117:74–91. doi: 10.1016/j.jpsychires.2019.07.003 31326751

