# Supplementary material for: Pharmacotherapy, acupoint stimulation, and psychotherapy for perimenopausal women with anxiety, depression, and panic disorder: a systematic review and network meta-analysis of randomized controlled trials
Source: Front Psychiatry. 2026 Jul 17;17:1845876. doi: 10.3389/fpsyt.2026.1845876 (PMC13423873; doi:10.3389/fpsyt.2026.1845876)
Supplement: Supplementary file 1 [file Supplementaryfile1.zip › Manuscript_Supplementary_Figure_Table/Supplementary Material 2-search stategy.docx]

# English-language databases

## Population-related terms

'**perimenopause**' OR '**climacterium**'

'climacteric' OR 'menopausal transition' OR 'perimenopausal female' OR 'perimenopausal woman' OR 'perimenopause' OR 'climacterium' OR 'perimenopausal' OR 'peri-menopausal'

'**anxiety disorder**' OR '**anxiety**'

'anxiety disorders' OR 'anxiety disorder' OR 'anxiety neuroses' OR 'neurotic anxiety state' OR 'neurotic anxiety states' OR 'generalized anxiety disorder' OR 'angst' OR 'nervousness' OR 'hypervigilance' OR 'social Anxiety' OR 'social Anxieties' OR 'anxiousness' OR 'anxiety'

'**depressive disorder**' **OR** '**perimenopausal depression**' **OR** '**depression**'

'depressive disorders' OR 'depressive neuroses' OR 'depressive neurosis' OR 'endogenous depression' OR 'endogenous depressions' OR 'melancholia' OR 'melancholias' OR 'unipolar depression' OR 'unipolar depressions' OR 'depressive syndrome' OR 'depressive syndromes' OR 'neurotic depression' OR 'neurotic depressions' OR 'climacteric depression' OR 'climacteric melancholy' OR 'climacterium depression' OR 'depression during menopausal transition' OR 'depression during menopause' OR 'menopausal depression' OR 'menopausal depressive disorder' OR 'menopausal depressive symptoms' OR 'menopausal depressive syndrome' OR 'menopausal onset depression' OR 'menopause-associated depression' OR 'menopause-induced depression' OR 'menopause-related depression' OR 'perimenopausal depressive disorder' OR 'perimenopausal depressive symptoms' OR 'perimenopause depressive symptoms' OR 'perimenopausal depression' OR 'central depression' OR 'clinical depression' OR 'depressive disease' OR 'depressive episode' OR 'depressive illness' OR 'depressive personality disorder' OR 'depressive state' OR 'depressive symptom' OR 'depressive syndrome' OR 'depressivity' OR 'mental depression' OR 'parental depression' OR 'depressive symptoms' OR 'depressive symptom' OR 'emotional depression' OR 'depressive disorder' OR 'depression'

'**panic disorder'**

'panic disorders' OR 'panic attacks' OR 'panic attack' OR 'panic' OR 'panics' OR 'panic disorder'

## Intervention-related terms

**'psychotropic drugs' OR 'psychotropic agent' OR 'antidepressant agent' OR 'Drug Therapy' OR 'Benzodiazepines' OR 'alprazolam' OR 'benzodiazepine derivative' OR 'Lorazepam' OR 'Serotonin 5-HT1 Receptor Agonists' OR 'buspirone' OR 'Pregabalin'**

'psychoactive agent' OR 'psychoactive drug' OR 'psychopharmaceutical' OR 'psychoactive agents' OR 'psychoactive drugs' OR 'psychotropic drug' OR 'psychopharmaceuticals' OR 'psychodynamic agent' OR 'psychopharmaceutic agent' OR 'psychopharmacon' OR 'psychotropic' OR 'psychotropic treatment' OR 'psychotropics' OR 'anti depressant agent' OR 'antidepressant' OR 'antidepressant drug' OR 'antidepressants' OR 'antidepressants, miscellaneous' OR 'antidepression drug' OR 'antidepressive agent' OR 'antidepressive agents' OR 'antidepressive agents, second generation' OR 'antidepressive agents, second-generation' OR 'antidepressive drug' OR 'neurothymoleptic agent' OR 'psychoenergizer' OR 'thymoleptic' OR 'thymoleptic agent' OR 'thymoleptic drug' OR 'thymolytic agent' OR 'antidepressant agent' OR 'antidepressant medication' OR 'antidepressant drugs' OR 'thymoanaleptics' OR 'thymoanaleptic' OR 'thymoleptics' OR 'Chemotherapy' OR 'Chemotherapies' OR 'Pharmacotherapy' OR 'Pharmacotherapies' OR 'Therapy, Drug' OR 'Drug Therapies' OR 'Therapies, Drug' OR 'drug treatment' OR 'medicament therapy' OR 'medicament treatment' OR 'medication' OR 'medicinal intervention' OR 'medicinal therapy' OR 'medicinal treatment' OR 'pharmaceutic intervention' OR 'pharmaceutical intervention' OR 'pharmaceutical therapy' OR 'pharmaceutical treatment' OR 'pharmaco-therapy' OR 'pharmaco-treatment' OR 'pharmacologic intervention' OR 'pharmacological intervention' OR 'pharmacological therapy' OR 'pharmacological treatment' OR 'pharmacotherapy' OR 'pharmacotreatment' OR 'therapeutic uses' OR 'therapy, drug' OR 'therapy, pharmacological' OR 'treatment, drug' OR 'treatment, pharmacological' OR 'drug therapy' OR 'Benzodiazepine Compounds' OR 'Benzodiazepine' OR ' Alprazolan' OR 'Xanax' OR 'Alprox' OR 'Apo-Alpraz' OR 'Apo Alpraz' OR 'Cassadan' OR 'Kalma' OR 'Novo-Alprazol' OR 'Novo Alprazol' OR 'Nu-Alpraz' OR 'Nu Alpraz' OR 'Ralozam' OR 'Esparon' OR 'Tafil' OR 'Trankimazin' OR '1, 4 benzodiazepin derivative' OR 'benzodiazepin derivative' OR 'benzodiazepines' OR 'benzodiazepinones' OR 'benzodiazepine derivative' OR '5-HT1A Agonist' OR '5 HT1A Agonist' OR 'Agonist, 5-HT1A' OR 'Buspar' OR 'Bespar' OR 'Anxut' OR 'gabica' OR 'psychotropic drugs' OR 'psychotropic agent' OR 'alprazolam' OR 'Lorazepam' OR 'Serotonin 5-HT1 Receptor Agonists' OR 'buspirone' OR 'Pregabalin'

'**acupuncture therapy**' **OR** '**acupuncture**' **OR 'Moxibustion'**

'acupuncture treatment' OR 'acupuncture treatments' OR 'pharmacoacupuncture treatment' OR 'pharmacoacupuncture therapy' OR 'acupotomy' OR 'acupotomies' OR 'shonishin' OR 'electroacupuncture' OR 'electro-acupuncture' OR 'auricular acupuncture' OR 'pharmacoacupuncture' OR 'acupressure' OR 'zhen jiu' OR 'traditional chinese medicine acupuncture' OR 'manual acupuncture' OR 'moxibustion' OR 'point embedding' OR 'transcutaneous electrical acupoint stimulation' OR 'auricular point' OR 'thumb-tack acupuncture' OR 'wrist-ankle acupuncture' OR 'warm acupuncture' OR 'Moxabustion' OR 'Moxibustion' OR 'acupuncture therapy' OR 'acupuncture'

'**psychotherapy' OR 'Cognitive Behavioral Therapy' OR 'mindfulness-based stress reduction'**

'holistic psychotherapy' OR 'multiple psychotherapy' OR 'psychotherapeutic processes' OR 'psychotherapeutic training' OR 'socioenvironmental therapy' OR 'psychotherapies' OR 'cognitive behavioral therapy' OR 'mindfulness' OR 'acceptance and commitment therapy' OR 'mindfulness-based stress reduction' OR 'interpersonal psychotherapy' OR 'psychological intervention' OR 'psychological therapy' OR 'counseling' OR 'MBSR Therapy' OR 'psychotherapy'

## Study design–related terms

'randomized controlled trial' OR 'random' OR 'placebo' OR 'randomised controlled study' OR 'RCT'

# Chinese-language databases

## 人群

主题词：围绝经期

更年期 + 绝经期 + 绝经期综合征 + 绝经综合征 + 围绝经期综合征 + 更年期综合征 + 更年期综合症 + 绝经过渡期 + 围绝经期

主题词：焦虑 + 焦虑症

神经质 + 过度警觉 + 情志障碍 + 焦虑障碍 + 焦虑 + 焦虑症

主题词：抑郁 + 抑郁症

情绪抑郁 + 抑郁症状 + 抑郁综合征 + 抑郁症 + 神经官能性 + 神经官能性抑郁症 + 忧郁症 + 单相抑郁症 + 抑郁性神经症 + 抑郁症, 内因性 + 内源性抑郁症 + 抑郁障碍 + 抑郁

主题词：惊恐障碍 + 惊恐 + 惊恐病

急性发作性焦虑 + 急性焦虑障碍 + 惊恐病 + 严重焦虑 + 恐慌 + 惊恐发作 + 惊恐症 + 惊恐障碍 + 惊恐 + 惊恐病

神经质 + 过度警觉 + 情志障碍 + 焦虑障碍 + 焦虑 + 焦虑症 + 情绪抑郁 + 抑郁症状 + 抑郁综合征 + 抑郁症 + 神经官能性 + 神经官能性抑郁症 + 忧郁症 + 单相抑郁症 + 抑郁性神经症 + 抑郁症, 内因性 + 内源性抑郁症 + 抑郁障碍 + 抑郁 + 急性发作性焦虑 + 急性焦虑障碍 + 惊恐病 + 严重焦虑 + 恐慌 + 惊恐发作 + 惊恐症 + 惊恐障碍 + 惊恐 + 惊恐病

## 干预

主题词：针灸疗法

针 + 针灸疗法 + 针刺 + 电针 + 穴位埋线 + 温针灸 + 温和灸 + 温针疗法 + 艾灸 + 穴位贴敷 + 耳针 + 针法 + 艾灸疗法 + 艾灸 + 大灸疗法 + 针灸 + 针灸治疗 + 温针 + 火针 + 毫针 + 体针 + 手针 + 灸法 + 隔物灸 + 雷火灸 + 麦粒灸 + 针刀 + 浮针 + 头针 + 电针 + 热敏灸 + 热敏灸法 + 悬灸 + 悬起灸 + 雀啄灸

主题词：药物疗法 + 抗抑郁药 + 抗焦虑药 + 精神药物 + 安定药 + 阿普唑仑 + 劳拉西泮 + 丁螺环酮

化学疗法 + 药物疗法 + 药物治疗 + 抗抑郁药物 + 抗抑郁制剂 + 抗抑郁剂 + 胸腺兴奋剂 + 抗焦虑制剂 + 抗焦虑剂 + 弱安定药 + 精神病药物 + 精神药物 + 作用于精神的药物 + 精神治疗药物 + 苯二氮䓬类 + 镇定药 + 阿普唑兰 + 阿普唑仑 + 安宁神 + 抗抑郁药 + 抗焦虑药 + 精神药物 + 安定药 + 劳拉西泮 + 丁螺环酮

主题词：心理疗法 + 心理治疗过程 + 人际心理治疗 + 认知行为疗法 + 正念

意义治疗 + 精神疗法 + 精神病治疗过程 + 谈话疗法 + 精神治疗 + 认知疗法 + 认知行为疗法 + 认知性心理疗法 + 心理疗法, 认知性 + 治疗, 认知 + 支持性心理治疗 + 心理疗法 + 心理治疗过程 + 人际心理治疗 + 认知行为疗法 + 正念

## 研究类型

主题词：随机对照试验

随机对照实验 + 随机对照研究
