# Supplementary material for: Pharmacotherapy, acupoint stimulation, and psychotherapy for perimenopausal women with anxiety, depression, and panic disorder: a systematic review and network meta-analysis of randomized controlled trials
Source: Front Psychiatry. 2026 Jul 17;17:1845876. doi: 10.3389/fpsyt.2026.1845876 (PMC13423873; doi:10.3389/fpsyt.2026.1845876)
Supplement: Supplementary file 1 [file Supplementaryfile1.zip › Manuscript_Supplementary_Figure_Table/Supplementary Material 3-baseline characteristics.docx]

| Study | Country | Patients | | | treatment | | | duration | disagonsis | outcome |
| --- | --- | --- | --- | --- | --- | --- | --- | --- | --- | --- |
|  |  | **Name** | **n** | **Age** | **Intervention** | **n** | **Age** |  |  |  |
| J Wang 2023 | china | Menopausal mood disorder | 95 |  | TCM_acupuncture; acupuncture；  TCM | 31；  32；  32 | 49.71(4.29)  50.45(4.70)  51.16(3.99) | 3months | the Clinical Practice Guideline of Chinese Medicine Climacteric Syndrome of the China Association of Chinese Medicine in 2012 | GCS/SDS/SAS/AE |
| Fei-Yi Zhao 2023 | china | comorbid perimenopausal depression and insomnia | 70 | 45-55 | acupuncture；  control | 35；  35 | 48.94(2.25)  48.80(2.07) | 8months | STRAW;  ICD-10;  ICSD-3 | HAMD/PSQI/Meno-D/ISI/KI/MenQoL/HAMA/SHBC/SCSQ/SRRS/FSH/E2/LH/AES/MS-TSQ/AE |
| Jayashri Kulkarni 2025 | Australia | menopausal depression | 53 | 40-65 | bazedoxifene_estrogen;  control | 20  17 | 53.90(5.88)  53.18(3.66) | 3months | DSM-IV | MADRS/Meno-D/MenQoL |
| Takahisa Ushiroyama 2004 | Japan | Depressed  Climacteric Patients | 113 |  | TCM;  antidepressants;  control | 58;  55;  34 | 52.8(6.3);  51.9(5.7);  52.0(6.0) | 3months | DSM-IV | HAMD/TNF-α |
| Linyan Li 2024 | china | Perimenopausal Insomnia  and Anxiety | 90 | 45-55 | MusicTherapy_APS;  alprazolam | 43;  42 | 49.0(2.60);  48.9(2.80) | 1month | (1)Eleventh Five-Year Plan” textbook “Obstetrics and Gynecology”;  (2) DSM-V | clinical efficacy/PSQI/HAMA/AE |
| Leslie Westlund Tam 2003 | USA | perimenopausal women diagnosed with major depression | 10 | 51-57 | fluoxetine_estrogen;  fluoxetine;  estrogen | 4  4  2 | 55;  53.75;  55.5 | 2months | DSM-IV | HAMD/BDI |
| Ching-Ling Kao 2012 | china | Peri- and Early  Postmenopausal Women with Anxiety | 50 | 40-60 | auricular acupressur；  control | 25  19 | 53.56(4.63)  54.42(6.42) | 1month |  | HAMA/MRS/AGI/SF-36/dose of Alprazolam/dose of Zolpidem |
| Asieh Mehdipou 2021 | Iran | perimenopausal  depression | 88 | 45-60 | emotional freedom  techniques；  control | 44  44 | 51.77(3.50);  52.40(2.85) | 2months |  | BDI |
| Derya Yu¨ksel Koc¸ak 2022 | Republic of Türkiye | menopausal  women with depression | 48 | 40-65 | music therapy  control | 21  27 | 59.1(4.2);  56.5(6.5) | 6weeks |  | BDI/MRS |
| Sheng Li 2018 | china | perimenopause women with mildmoderate depressive symptom | 242 |  | electroacupuncture;  escitalopram | 116  105 | 49.83(3.10)  49.93(3.10) | 3months | DSM-V and ICD-10 for the mild-moderate depression; the total score of HAMD is <23 and ≥8 | HAMD/MENQOL/E2/FSH/LH |
| Xiao-jing Cao 2019 | china | perimenopausal depression | 320 | 41-60 | TCM_psychotherapy;  psychotherapy | 151  156 | 50.5(4.8);  50.8(5.1) | 5months | SDS and SAS scores of 50 or higher | GCS/SAS/SDS/E2/TG/TC/HDL-C/LDL-C/FSH/AE |
| Peter J. Schmidt 2015 | USA | Women With Perimenopausal Depression | 30 | 45-65 | estrogen;  control | 12  14 | 54.8(5.2);  56.1(3.8) | 6weeks | DSM-IV | CESD-10/HAMD/BDI |
| Robab Khoshbooii 2021 | Iran | depressed Iranian perimenopausal women | 64 | 40-55 | Cognitive Behavioral Therapy;  control | 42  22 | 47.23(4.69)  48.63(4.63) | 8months | BDI-II score of 21–46 | BDI-II/ENRICH |
| Cla´udio de Novaes Soares 2001 | Australia | Depressive  Disorders in Perimenopausal Women | 75 | 40-55 | estrogen;  control | 25  25 | 49.3(3.8)  50.3(3.4) | 3months | DSM-IV | MADRS/KI |
| Xin Liu 2023 | china | generalized anxiety disorder in perimenopausal women | 112 | 45-55 | acupuncture;  control | 44  44 | 49.00(3.70)  50.00(2.96) | 1month | DSM-V | HAMA/GAD7/PSQI/ACTH/CORT |
| Peter J. Schmidt 2022 | USA | women with perimenopausal depression | 66 | 40-60 | estrogen;  control | 44  18 | 49.97(2.86) 51.3(3.1) | 2months | DSM-IV | CESD/HAMD/BDI/VAS/E2/FSH/LH/Cognitive performance scores |
| Jennifer A. Linde 2011 | USA | Women with Comorbid Obesity and Depression | 203 | 52 | behavioral weight loss；  behavioral weight loss_ CBT | 102  101 | 52.1(6.5)；  52.3(6.2) | 12 months | The Structured Clinical Interview for DSM-IV or SCID mood module | weight/SCL-20/PAQ/food frequency questionnaire |
| James A. Blumenthal 1999 | USA | Older Patients With Major Depression | 156 | ≥50 | sertraline;  control | 48  53 | 57(7.0)  57(5.8) | 4months | DSM-IV | HAMD/BDI/STAI/Rosenberg Self-Esteem Scale/Life Satisfaction Index/Dysfunctional Attitudes Scale |
| Carlos Berlanga 2003 | Mexico | perimenopausal outpatients with a major depressive disorder | 31 | 45-65 | fluoxetine_tibolone;  fluoxetine | 16  15 | 53.0(2.6);  54.4(5.6) | 2months | DSM-IV | HAMD/GCS/FSH/E2/LH |
| Susan G. Kornstein 2015 | USA | Major Depressive Disorder in Perimenopausal Women | 252 | 47.2(3.9) | desvenlafaxine;  control | 147  105 |  | 2months | DSM-IV | HAMD/MADRS/CGI-S/SDS/MRS/AE |
| Yuyan Zeng 2019 | china | menopausal women with moderate to serious mood disorder | 85 | 41-60 | TCM_psychotherapy;  TCM | 43  42 | 51.91(3.83)  50.44(4.70) | 3months | DSM-IV | GCS/SAS/SDS/DA/NA |
| Jun-He Zhou 2022 | china | mild to moderate  perimenopausal depression | 242 | 45-55 | electroacupuncture; escitalopram | 108  108 |  | 12months | (1)STRAW-10 in 2012  (2)DSM-5 criteria | HAMD/MENQOL/FSH/E2/AE |
| Susan G. Kornstein 2010 | USA | Perimenopausal and  Postmenopausal Women With Major Depressive Disorder | 387 | 40-70 | desvenlafaxine;  control | 247  125 | 52(6)  53(7) | 2months | DSM-IV | HAMD/CGI-I/MADRS/HAMA/CGI-S/EQ-5D/MRS/SDS/QIDS-SR/VAS-PI |
| Jayashri Kulkarni 2018 | Australia | Women with perimenopausal major depressive disorder | 44 | 45-65 | tibolone;  control | 22  22 | 52.8(5.75)  51.0(4.69) | 3months |  | MADRS/AE |
| Hadine Joffe 2011 | USA | peri/postmenopausal women with depression disorders | 72 | 40-60 | estrogen;  zolpidem;  control | 27  31  14 | 50.0(4.8)  51.3(5.1)  52.6(4.9) | 2 months | DSM-IV；have a MADRS score of 15–31 | E2/PSQI/DDHF |
| Chao Yang 2025 | china | perimenopausal  depression | 64 | 45-55 | estrogen;  control | 32  32 | 51.81(2.53)  50.63(2.14) | 1 month |  | KI/SAS/SDS/E2/FSH/P/LH |
| Natalie L. Rasgon 2002 | USA | Major Depressive Disorder in Perimenopausal Women | 16 | 46.7(3.3) | estrogen  fluoxetine_estrogen | 10  6 |  | 2 months | DSM-IV | HAMD |
| Zhou Qing 2009 | china | perimenopausal  depression | 90 | 41-60 | acupuncture  Premarin_Medroxyprogesterone | 60  30 | 48.40(4.50)  48.98(4.66) | 3 months | CCMD222R | HAMD/clinical efficacy |
| Zhao Bing 2015 | china | perimenopausal  depression | 86 | 42-57 | fluoxetine_estrogen_TCM_psychotherapy;  fluoxetine_estrogen | 43  43 | 51.87(4.44)  50.67(5.43) | 3 weeks | CCMD-3; HAMD＞17； HAMA＞14; KI≥11 | HAMD/HAMA/KI/EPQ |
| Li Na 2012 | china | menopausal depression | 96 | 41-60 | TCM  acupuncture  TCM_acupuncture | 32  32  32 | 50.98(4.85)  50.14(4.47)  51.06(4.31) | 2 months | HAMD；KI＞15 | HAMD/clinical efficacy |
| Zhang Chunhong 2013 | china | perimenopausal  depression | 80 | 43-56 | fluoxetine_estrogen  fluoxetine_estrogen_TCM | 40  40 | 45.4(3.2)  46.8(4.1) | 3 weeks | CCMD-3; HAMD＞18； | HAMD/clinical efficacy |
| Wang Shufang 2015 | china | perimenopausal  depression | 80 | 42-50 | fluoxetine_estrogen  fluoxetine_estrogen_TCM | 40  40 | 45.82(5.45)  46.45(6.15) | 3 weeks | CCMD-3; HAMD≥18； | HAMD |
| Yuan Zheng 2015 | china | perimenopausal  depression | 60 | 47.8(3.6) | citalopram  citalopram_TCM | 30  30 |  | 2 months | HAMD≥18； | HAMD |
| Liu Ru 2007 | china | menopausal depression | 60 |  | citalopram_TCM  citalopram | 30  30 | 54.32(3.29)  54.0(4.62) | 2 months | CCMD-3; HAMD≥18； | HAMD/clinical efficacy |
| Liu Jiahong 2023 | china | comorbid depression in perimenopausal type 2 diabetes | 68 |  | paroxetine  paroxetine_TCM | 34  34 | 46.00(3.10)  46.50(3.00) | 6 weeks | HAMD7~24 | clinical efficacy/E2/FSH/LH/AE |
| Chen Hui 2012 | china | perimenopausal  depression | 119 | 40-60 | fluoxetine; fluoxetine_TCM | 51; 68 | 50.78(4.08); 50.20(3.94) | 2 months | HAMD＞21; CCMD3 | clinical efficacy/HAMD/5-HT/NE/AE/endometrial thickness |
| Ma Yanbing 2011 | china | perimenopausal depression | 105 |  | paroxetine; paroxetine_electroacupuncture | 50; 55 | 51.49(6.03); 52.95(5.86) | 6 weeks | CCMD-3; HAMD≥20； | clinical efficacy/HAMD |
| Shi Xiaolan 2011 | china | perimenopausal depression | 120 | 40-60 | electroacupuncture; TCM; TCM_electroacupuncture | 40; 40; 40 | 51.15(1.52); 52.3(1.67); 52.2(1.67) | 1 month | CCMD-3; HAMD＞20 | KI/HAMD/E2 |
| Lu Meijuan 2019 | china | perimenopausal depression | 100 | 40-60 | TCM; TCM_acupuncture | 50; 50 | 49.6(1.4); 47.5(1.3) | 3 months | CCMD-3 | clinical efficacy/HAMD/KI/endometrial thickness |
| Huang Hongli 2017 | china | perimenopausal depression | 60 | 40-60 | TCM; TCM_acupuncture | 30; 30 | 44-56; 41-58 | 3 months | CCMD-3;DSM-V;KI＞15;HAMD≥21 | clinical efficacy/HAMD/E2/FSH/LH/T/PRL/P/endometrial thickness |
| Zheng Lin 2018 | china | perimenopausal depression | 80 | 45-55 | acupuncture;  fluoxetine | 40; 40 | 50.78(0.54); 50.82(0.58) | 6 weeks | CCMD-3; HAMD＞20 | HAMD/clinical efficacy/E2/FSH/LH |
| Shen Hui 2016 | china | perimenopausal anxiety disorder | 60 | 40-55 | acupuncture; acupuncture_group psychotherapy | 30; 30 | 46.97(5.02); 48.73(5.15) | 6 weeks | CCMD-3; HAMA≥18，＜30；HAMD≤7；KI≥5 | HAMA/KI/SAS |
| Ma Jing 2009 | china | perimenopausal depression | 60 | 45-60 | acupuncture;  fluoxetine | 30; 30 | 53.45(4.82); 52.74(5.17) | 2 months | CCMD-3; HAMD≥18 | HAMD/clinical efficacy/AE |
| Ning Ying 2015 | china | menopausal depression | 90 | 40-60 | estrogen; estrogen_acupuncture | 45; 45 | 46-55; 45-57 | 3 months | HAMD＞7 | HAMD/clinical efficacy/TESS |
| Qiang baoquan 2008 | china | menopausal depression | 60 |  | acupuncture;  fluoxetine | 30; 30 | 54.32(3.29); 54.0(3.6) | 6 weeks | CCMD-3; HAMD≥18 | HAMD/clinical efficacy |
| Chen Zhong 2010 | china | menopausal depression | 60 |  | acupuncture;  fluoxetine | 30; 30 | 51.8(4.2); 48.9(3.8) | 6 weeks | CCMD-3; HAMD＞17; KI＞15 | HAMD/HAMA |
| Niu Xuesong 2017 | china | Menopausal depression | 82 | 45-60 | acupuncture;  fluoxetine | 41; 41 | 54.1(2.0); 54.2(2.1) | 6 weeks | CCMD-3; HAMD＞17; KI＞15 | HAMD/clinical efficacy/AE/TCM syndrome score |
| Bai Yanfu 2016 | china | Depression in perimenopausal women | 40 | 40-60 | acupuncture;  fluoxetine | 20; 20 | 47.22(7.62); 48.02(7.85) | 2 months | CCMD-3; HAMD20-35 | HAMD/clinical efficacy |
| Gu Ting 2020 | china | perimenopausal mild depression | 60 | ≤55 | TCM_acupuncture; TCM | 30; 30 | 49(3); 50(3) | 3 months | DSM-IV | HAMD/SDS/KI/clinical efficacy |
| Xie Yuqing 2013 | china | menopausal depression | 60 | 40-60 | TCM_acupuncture; fluoxetine | 30; 30 | 53; 51.5 | 1 month | DSM-IV;HAMD＞19;SDS＞55 | HAMD/clinical efficacy/SDS |
| Zhang Yan 2025 | china | Menopausal anxiety | 106 | 45-55 | paroxetine; paroxetine_acupuncture | 53; 53 | 45-55; 45-54 | 2 months | HAMA 4～29 | HAMA/SAS/PSQI/KI/FSH/LH/E2/P/NE/5-HT/ACTH/AE |
| Liu Heping 2018 | china | menopausal depression | 80 | 40-60 | sertraline; sertraline_acupuncture | 40; 40 | 52.1(3.3); 51.5(3.4) | 3 months | HAMD≥20 | HAMD/FSH/E2/5-HT/GABA/clinical efficacy |
| Zhang Xi 2021 | china | perimenopausal depression | 60 | 45-55 | acupuncture_Wheat grain moxibustion; fluoxetine | 30; 30 | 50(3); 49(2) | 2 months | CCMD-3; HAMD≥18 | HAMD/KI/clinical efficacy |
| Dong Yan 2015 | china | menopausal depression | 60 | 46-58 | acupuncture; fluoxetine_estrogen | 30; 30 | 55; 53 | 1 month | CCMD-3; HAMD≥18 | HAMD/clinical efficacy |
| Chi Hui 2011 | china | perimenopausal depression | 60 | 45-55 | acupuncture;  fluoxetine | 30; 30 | 51.63(1.72); 51.43(1.62) | 1 month | CCMD-3; HAMD18~35 | HAMD/clinical efficacy/AE |
| Qin Erqi 2019 | china | Mild to moderate depression during perimenopause | 130 | 44-55 | ACE;  fluoxetine | 65; 65 | 51.23(1.32); 50.12(1.57) | 2 months | STRAW -10;ICD-10 | HAMD/MRS/SERS |
| Sun Zhanling 2015 | china | Perimenopausal patients with mild depression | 46 | 40-60 | ACE;  acupuncture | 23; 22 | 45(4); 47(4) | 2 months | CCMD-3; HAMD7-17 | KI/HAMD |
| Xing Kai 2011 | china | menopausal depression | 240 | 45-60 | acupuncture;  fluoxetine | 120120 | 51.2(5.4); 49.5(6.8) | 6 weeks | CCMD-3; HAMD≥20 | clinical efficacy/HAMD |
| Gao Yonglian 2011 | china | Menopausal syndrome and anxiety disorders in women | 66 | 45-60 | estrogen_anti-anxiety medications; psychotherapy | 33; 33 | ; | 2 months | CCMD-3 | clinical efficacy/SAS |
| Zou Yuhong 2013 | china | menopausal depression | 103 |  | pharmacotherapy; pharmacotherapy_psychotherapy | 49; 54 | 49.5(7.4); 47.9(6.6) | 1 month | CCMD-3 | SAS/SDS |
| Yang Xiaoqu 2012 | china | First episode of menopausal depression | 60 | 46 | citalopram_quetiapine; citalopram | 30; 30 | 43; 45 | 2 months | CCMD-3;HAMD≥18 ;HAMA≥14 | HAMD/HAMA/clinical efficacy |
| Wang Fei 2011 | china | First episode of menopausal depression | 64 | 40-65 | citalopram; citalopram_quetiapine | 32; 32 | 53.8(13.4); 53.4(12.6) | 2 months | CCMD-3;HAMD≥18 ;HAMA≥14 | HAMD/HAMA |
| Qian Jie 2007 | china | menopausal depression | 66 | 45-60 | acupuncture;  fluoxetine | 33; 33 | 54; 55 | 6 weeks | CCMD-3;HAMD≥17,≤30 | HAMD;AE |
| Ding Xinhua 2023 | china | perimenopausal anxiety disorder | 90 | 42-55 | duloxetine_group psychotherapy; duloxetine | 45; 45 | 49.20(3.62); 48.15(3.81) | 1 month |  | HAMA/PSQI |
| Xing Xiaojuan 2021 | china | Sleep disorders in perimenopausal patients with anxiety | 80 | 42-56 | scalp acupuncture; acupuncture | 40; 40 | 49.2(4.6); 48.2(3.6) | 1 week | HAMA＞15 | HAMA/clinical efficacy |
| Qiu Lianli 2014 | china | Perimenopausal depression | 68 | 40-50 | acupuncture_psychotherapy;  acupuncture | 34; 34 | 44.71(3.50); 44.43(2.88) | 2 months | SDS≥40;ICD-10;Greene≥10 | clinical efficacy/GCS/HAMD |
| Liu Xiaoqing 2023 | china | Perimenopausal depression | 80 | 42-58 | delexin_TCM_acupuncture;  delexin | 40; 40 | 49.18(2.21); 48.89(2.14) | 2 months |  | HAMD/clinical efficacy/KI/E2/FSH/LH |
| Zheng Shenghui 2010 | china | Perimenopausal depression | 120 | 40-60 | fluoxetine_progesterone;  acupuncture | 60; 60 | 52.27(3.45); 51.98(3.14) | 3 months | KI≥17; HAMD≥16 | clinical efficacy/HAMD/KI/E2/FSH/LH/AE |
| Fan Yumei 2024 | china | Menopausal hypertension with anxiety | 70 | 45-55 | delexin;  delexin_TCM | 35; 35 | 51.46(3.24); 51.35(3.30) | 6 weeks | CCMD-3 | clinical efficacy/HAMD/HAMA/PSQI/AE |
| Shen Feier 2025 | china | Perimenopausal depression | 100 | 49-56 | TCM_acupuncture; acupuncture | 50; 50 | 52.19(1.27); 52.12(1.22) | 2 months |  | KI |
| Zhou Yanping 2022 | china | Perimenopausal depression | 100 | 48-55 | acupuncture; TCM_acupuncture | 50; 50 | 53.4(1.5); 53.6(1.3) | 2 months |  | KI |
| Liang Ziqing 2024 | china | Mild to moderate perimenopausal depressive disorder | 66 | 40-60 | sertraline; sertraline_acupuncture | 33; 33 | 51.14(2.32); 51.35(2.18) | 2 months | ICD-10;STRAW+10;HAMD8-23 | HAMD/MENQOL/LH/FSH/E2/clinical efficacy |
| Li Yuling 2026 | china | menopausal depression | 90 | 45-55 | paroxetine_estrogen_electroacupuncture; paroxetine_estrogen | 45; 45 | 49.93(4.87); 50.32(5.32) | 3 months | HAMD≥ 7;ICD-11 | HAMD/HAMA/KI/E2/FSH/LH/P/NE/5-HT/DA/clinical efficacy/AE |
| Dong Chunxiu 2021 | china | perimenopausal depression | 60 | 45-60 | sertraline_acupuncture; sertraline | 30; 30 | 51.2(6.2); 49.4(10.1) | 1 month | HAMD8-35 | HAMD/MENQOL/AE |
| Lou Lifang 2012 | china | Menopausal depression in women | 62 |  | pharmacotherapy; pharmacotherapy_CBT | 31; 31 | 48.5(7.6); 47.6(8.0) | 3 weeks | HAMD＞14 | SDS |
| Fu Jianguo 2011 | china | perimenopausal depression and anxiety | 60 | 45-55 | fluvoxamine maleate; fluvoxamine maleate_ CBT | 30; 30 |  | 3 months | HAMD≥ 18 | SDS/HAMA |
| Zheng Guangxian 2016 | china | perimenopausal depression | 60 | 45-55 | heat sensitive moxibustion;  delexin | 30; 30 | 50.36(12.35); 51.21(13.13) | 1 month | CCMD-3;HAMA≥14; HAMD≥7 | clinical efficacy/HAMD |
| Cheng Xuejun 2021 | china | perimenopausal depression | 60 |  | clomipramine; citalopram | 30; 30 | 36.35(8.45); 38.31(8.12) | 6 months |  | HAMD/MMSE/AE |
| Zhang Cuihua 2011 | china | perimenopausal depression | 60 | 45-55 | paroxetine; paroxetine_TCM | 30; 30 | 42.25(11.38); 43.50(12.43) | 6 weeks | CCMD-3;HAMD≥18 | clinical efficacy/HAMD/CGI/AE |
| Zhu Xinyang 2020 | china | perimenopausal depression | 86 | 43-62 | paroxetine; paroxetine_olanzapinee | 43; 43 | 45.8(6.1); 47.9(6.2) | 1 month | ICD-10; HAMD≥18 | clinical efficacy/HAMD/HAMA/SCL-90/AE |
| Zheng Chunming 2013 | china | menopausal depression | 50 | 45-65 | amitriptyline; sertraline_progesterone | 24; 26 | 48.53(3.62); 54.42(4.14) | 2 months | CES-D ≥20; HAMD17 ≥17 | clinical efficacy/HAMD/HAMA |
| Huo Jun 2008 | china | menopausal anxiety | 111 | 45-55 | TCM;  alprazolam | 56; 55 | 50.36(2.85); 51.03(2.38) | 2 weeks | CCMD-3 | clinical efficacy/HAMA/AE |
| Cai Yan 2022 | china | menopausal anxiety | 108 | 45-55 | acupuncture; TCM_acupuncture | 54; 54 | 49.31(1.26); 49.12(1.15) | 1 month | "Expert Consensus on Diagnosis and Treatment of Anxiety, Depression and Somatization Symptoms in General Hospitals" | clinical efficacy/E2/FSH/LH/5-HT/COR/ACTH/GnRH/IL-2/INF-γ/IL-4/IL-10/KI/BAI |
| Dong Caijuan 2025 | china | perimenopausal depression | 120 | 45-60 | acupuncture; TCM_acupuncture | 60; 60 | 50.28(3.22); 50.37(3.24) | 1 month | CCMD-3;HAMD≥21；KI＞15 | clinical efficacy/KI/PSQI/TCM syndrome score/E2/FSH/LH/5-HT/NE/AE |
| Qin Lulu 2022 | china | perimenopausal depression | 60 | 45-55 | delexin; delexin_TCM_acupuncture | 30; 30 | 50.36(3.52); 50.53(2.68) | 2 months | CCMD-3;HAMD＞8 | clinical efficacy/HAMD/SDS/KI/FSH/LH/E2/5-HT/NE/DA/GABA/Gly |
| Peng Baoan 2012 | china | perimenopausal depression | 120 | 44-60 | fluoxetine_tibolone; fluoxetine;  tibolone | 40; 40; 40 | 49(8); 50(9); 48(9) | 2 months | CCMD-3 | clinical efficacy/HAMD |
| Wang Kuiyuan 2016 | china | menopausal anxiety disorder | 74 | 47-60 | psychotherapy; TCM_psychotherapy | 37; 37 |  | 2 months | CCMD-3 | clinical efficacy/AE/PRL/E2 |
| Luo Ying 2020 | china | perimenopausal anxiety disorder | 60 |  | duloxetine;  CBT | 30; 30 | 45.32(2.44); 45.18(2.30) | 1 month |  | SAS/HAMA/PSQI/clinical efficacy |
| Wang Wenhui 2014 | china | menopausal women with depression or anxiety | 80 | 45-55 | paroxetine;  tibolone; paroxetine_tibolone; TCM | 20; 20; 20; 20 |  | 2 months | SDS＞53;SAS＞50 | HAMA/HAMD/GCS |
| Fu Ru 2018 | china | menopausal anxiety | 60 | 43-58 | buspirone_TCM; oryzanol | 30; 30 | 18.0(14.1); 51.1(5.54) | 6 weeks | HAMA＞7; | HAMA/clinical efficacy |
| Liu Yujie 2013 | china | women with menopausal symptoms and affective disorders | 120 | 45-57 | estrogen;  sertraline;  control | 40; 40; 40 |  | 6 weeks | HAMD≥20 | HAMD/HAMA |
| Li Ziyan 2021 | china | perimenopausal depression | 104 | 44-59 | amitriptyline; amitriptyline_SEPT | 52; 52 | 53.5(4.6); 53.4(4.2) | 3 months | CCMD-3 | clinical efficacy/HAMD/AE |
| Li Qiao 2017 | china | Depression in perimenopausal women | 100 | 43-61 | amitriptyline; amitriptyline_SEPT | 50; 50 | 52.6(3.8); 52.3(3.7) | 3 months | CCMD-3 | clinical efficacy/HAMD/AE |
| Wu Jing 2023 | china | menopausal depression | 86 | 45-55 | citalopram; citalopram_Musictherapy_AP | 43; 43 | 51.22(2.12); 51.24(2.13) | 2 months | CCMD-3 | HAMD/PSQI/FSH/LH/E2 |
| Zhong Yan 2025 | china | perimenopausal depression | 82 |  | pharmacotherapy; pharmacotherapy_psychotherapy | 41; 41 | 52.53(4.27); 52.68(4.53) | 2 months |  | clinical efficacy/HAMD/KI/FSH/LH/E2/PSQI |
| Bi Fuxi 2024 | china | Menopausal syndrome with anxiety disorder | 60 | 40-60 | livmin; livmin_acupuncture | 30; 30 | 49.21(8.44); 48.84(8.23) | 2 months | 14≤ HAMA< 20 ;KI＞14; | clinical efficacy/KI/HAMA/E2/FSH/LH/VIP/SS/NPY/CGRP/5-HT |
| Zheng Kai 2007 | china | menopausal depression | 62 | 48-52 | estrogen; fluoxetine_estrogen_psychotherapy | 29; 33 |  | 6 weeks | Zung＞40；HAMD>17 | Zung score/HAMD |
| Wang Xiaoyun 2010 | china | menopausal depression | 60 | 41-60 | acupuncture;  delexin | 30; 30 | 49.6(4.3); 48.3(4.7) | 1 month | HAMD＞7 | HAMD/AE/clinical efficacy |
| Lu Yanfang 2009 | china | menopausal depression | 120 | ＞45 | estrogen; fluoxetine_estrogen | 59; 61 | 50.31(3.69); 49.93(4.53) | 2 months | CCMD-3; HAMD≥18; | HAMD/KI |
| Pan Zhenwei 2019 | china | menopausal depression | 60 | 45-55 | delexin;  paroxetine | 30; 30 | 50.31(3.07); 49.44(2.76) | 2 months | ICD-10;HAMD≥17 | HAMD/AE/clinical efficacy |
| Gao Jing 2023 | china | perimenopausal depression | 80 | 40-60 | ear acupuncture;  control | 40; 40 | 53.45(5.67); 53.10(5.24) | 3 months | KI≥15 ;SDS 50-73 | SDS/KI/E2/FSH/LH/clinical efficacy |
| Li Longfen 2013 | china | Menopause Anxiety | 30 | ＜55 | delexin; delexin_acupuncture | 15; 15 | 45-53; 43-52 | 2 months | HAMA＞21 | clinical efficacy/HAMA/AE |
| Xia Shiyan 2014 | china | Depression in perimenopausal women | 58 | 45-58 | duloxetine;  citalopram | 29; 29 | 48(7); 48(7) | 2 months | CCMD-3 | HAMD/HAMA/AE |
| Wu Yi 2022 | china | Perimenopausal depression (liver depression type) | 60 | 45-55 | TCM; TCM_electroacupuncture | 30; 30 | 49.51(3.23); 49.60(3.12) | 2 months | CCMD-2 | KI/HAMD/clinical efficacy |
| Shi Jia 2018 | china | Mild to moderate perimenopausal depressive disorder | 60 | 46-55 | citalopram; electroacupuncture | 30; 30 | 49.93(1.87); 48.70(1.99) | 3 months | DSM-V; HAMD 8-23 | HAMD/clinical efficacy |
| Sun Yanjie 2015 | china | Mild to moderate perimenopausal depressive disorder | 42 | 44-55 | citalopram; electroacupuncture | 21; 21 | 49.86(3.83); 50.29(2.59) | 3 months | STRAW-10;DSM-V;HAMD 7-23 | HAMD/clinical efficacy |
| Li Ping 2020 | china | menopausal depression | 60 | 40-60 | fluoxetine; electroacupuncture | 30; 30 | 45-57; 43-55 | 6 weeks | HAMD≥18 | HAMD/HAMA/clinical efficacy |
| Guo Yaming 2005 | china | menopausal depression | 105 | 41-65 | amitriptyline; amitriptyline_electroacupuncture | 50; 55 | 51.49(6.03); 52.95(5.86) | 6 weeks | CCMD-2;HAMD≥18 | clinical efficacy/HAMD |
| Men Shaojie 2022 | china | perimenopausal depression | 64 | ≥ 40 | citalopram_tibolone; citalopram_tibolone_electroacupuncture | 32; 32 | 51.29(5.26); 51.23(5.17) | 3 months | CCMD-3 | SDS/PSQI/FSH/LH/E2/5-HT/DA/NE/ACTH |
| Tang Nanlin 2019 | china | Mild to moderate perimenopausal depression | 60 | 40-60 | electroacupuncture; TCM; TCM_electroacupuncture | 20; 20; 20 | 49.52(7.58); 48.93(7.30); 49.14(7.25) | 1 month | HAMD≥7 | clinical efficacy/HAMD/ACTH/CORT/AE |
| Dai Wei 2022 | china | perimenopausal depression | 80 | 45-53 | citalopram; citalopram_electroacupuncture | 40; 40 | 48.51(3.32); 48.68(2.28) | 1 month | 24≥ HAMD ≥17 | HAMD/HAMA/KI/PSQI/AE |
| Tan Jie 2010 | china | perimenopausal depression | 62 |  | TCM_acupuncture; acupuncture | 31; 31 | 50.45(4.81); 50.17(5.18) | 2 weeks | HAMD≥18;CCMD-3 | clinical efficacy |
| Lu Xianglian 2022 | china | Perimenopausal syndrome with anxiety disorders | 70 |  | SEPT; SEPT_alprazolam | 35; 35 | 51.62(4.13); 51.37(4.27) | 3 months | Diagnostic and Statistical Manual of Mental Disorders | clinical efficacy/E2/FSH/LH/HAMA/MENQOL/5-HT/NE/β-EP |
| Xie Shuwen 2005 | china | Menopausa depression in women | 60 | 46-55 | fluoxetine_estrogen; estrogen | 30; 30 |  | 3 months | ICD-10 | HAMD/KI |
| Yu Xuewen 2007 | china | Perimenopause and menopausal depressive disorders | 61 | 45-55 | paroxetine_tibolone; tibolone | 30; 31 |  | 2 months | HAMD≥17;ICD-10;CCMD-3 | HAMD/KI |
| Li Jin 2022 | china | menopausal depression | 150 | 45-55 | delexin; delexin_acupuncture; TCM_acupuncture | 50; 50; 50 | 49.8(2.10); 49.20(2.02); 50.01(2.31) | 2 months | HAMD≥ 17，HAMA≥ 14 | HAMA/KI/PSQI/HAMD/clinical efficacy/LH/FSH/E2/AE |
| Chen Yahong 2023 | china | menopausal depression | 60 |  | delexin; delexin_TCM_acupuncture | 30; 30 | 49.53(2.03); 49.12(2.11) | 2 months |  | clinical efficacy/LH/FSH/E2/HAMA/HAMD/PSQI/KI |
| Yu Yang 2023 | china | perimenopausal depression | 60 | 40-60 | venlafaxine; TCM_acupuncture | 30; 30 | 49.17(3.43); 48.50(2.80) | 6 weeks | DSM-V;CCMD-3 | HAMD/KI/TCM syndrome score/clinical efficacy |
| Zhang Daifen 2025 | china | menopausal depression | 120 | 46-55 | delexin; TCM_acupuncture | 60; 60 |  | 2 months |  | KI/SDS/SAS/clinical efficacy/E2/FSH/LH/NE/DA/5-HT/AE |
| Ji Li 2021 | china | menopausal depression | 72 | 54-74 | escitalopram; escitalopram_CBT | 36; 36 | 64.15(2.19); 64.24(2.21) | 2 months | CCMD-3 | HAMA/HAMD/CGI-S |
| Qian Jun 2015 | china | Depression in perimenopausal and postmenopausal women | 106 | 46-61 | SEPT; fluoxetine_psychotherapy | 38; 38 | 56.32(2.47); 57.02(2.86) | 3 months |  | clinical efficacy/HAMD/HAMA |
| Lai Ailuan 2007 | china | Depression in perimenopausal and postmenopausal women | 86 | 44-59 | delexin;  fluoxetine;  tibolone | 29; 14; 43 |  | 3 months | HAMD＞20;CCMD-2 | HAMD/SDS |
| Che Jinxiang 2020 | china | menopausal depression | 86 | 41-61 | TCM; TCM_acupuncture | 43; 43 | 48.37(3.62); 49.32(3.54) | 2 months | CCMD-3 | FSH/LH/E2 |
| Zhang Dequn 2021 | china | perimenopausal depression | 82 | 45-55 | SEPT; TCM_acupuncture | 41; 41 | 49.54(1.58); 50.22(1.63) | 1 month | HAMD＞17 | HAMD/FSH/E2/LH/PSQI |
| Shen Jiewen 2017 | china | perimenopausal depression | 60 | 41-60 | TCM; TCM_acupuncture | 30; 30 | 49.93(3.69); 49.00(4.24) | 3 months | CCMD-3; HAMD≥21;KI＞17 | HAM/KI/E2/FSH/LH/AE |
| Ding Li 2007 | china | perimenopausal depression | 78 |  | fluoxetine;  acupuncture | 39; 39 | 49.50(3.51); 49.68(3.90) | 1 month | KI≥17；HAMD＞16 | HAMD/KI |
| Jin Yabei 2013 | china | perimenopausal depression and anxiety | 92 | 40-66 | ACE;  acupuncture | 46; 46 | 47.85(4.32); 48.52(4.20) | 2 months | HAMD＞18 | HAMD/FSH/E2/LH/clinical efficacy |
| Chen Xianguang 2007 | china | perimenopausal depression | 170 | 46-55 | fluoxetine_estrogen; estrogen;  fluoxetine | 60; 60; 50 |  | 3 months | ICD-10 | clinical efficacy/HAMD/KI/E2 |
| Jiang Xiaoyan 2009 | china | First episode of menopausal depression | 70 | 45-55 | citalopram;  paroxetine | 35; 35 | 48.7(3.6); 48.5(4.2) | 2 months | CCMD-3;HAMD≥18 | HAMD/HAMA/clinical efficacy |
| Wang Junming 2020 | china | Perimenopausal insomnia accompanied by anxiety and depression | 80 | 40-60 | estazolam;  agomelatine | 40; 40 | 44.3(4.1); 42.8(3.2) | 6 weeks | PSQOI＞7; SAS＞50; SDS＞50 | PSQI/SAS/SDS/SERS |
| Song Guixia 2022 | china | perimenopausal anxiety and depression disorder | 134 | 48-61 | paroxetine; paroxetine_electroacupuncture | 67; 67 | 54.12(4.73); 54.28(4.64) | 6 weeks | Diagnostic and Statistical Manual of Mental Disorders | HAMA/HAMD/E2/LH/P/FSH |

**Intervention abbreviations,**

ACE, Acupoint Catgut Embedding

APS, auricular point seed burying

CBT, cognitive behavioral therapy

EFT, emotional freedom techniques

SEPT, Sequential Estrogen-Progestogen Therapy

TCM, Traditional Chinese Medicine

**disagonsis abbreviations,**

CCMD-2R/3, Chinese Classification and Diagnostic Criteria of Mental Disorders, Second Revised/Third Edition

DSM-5 / DSM-IV, Diagnostic and Statistical Manual of Mental Disorders, 5th / 4th Edition

ICD-10 / ICD-11, International Classification of Diseases, 10th / 11th Revision

ICSD-3, International Classification of Sleep Disorders, 3rd Edition

SCID, Structured Clinical Interview for DSM

STRAW +10, Stages of Reproductive Aging Workshop +10 criteria

**Outcome abbreviations:**

5-HT, 5-Hydroxytryptamine (Serotonin)

ACTH, Adrenocorticotropic Hormone

AES, Acupuncture Expectancy Scale

BAI, Beck Anxiety Inventory

BDI / BDI-II, Beck Depression Inventory / Beck Depression Inventory-II

β-EP, Beta-Endorphin

CESD / CESD-10, Center for Epidemiologic Studies Depression Scale / CESD-10

CGRP, Calcitonin Gene-Related Peptide

CGI-I / CGI-S, Clinical Global Impression-Improvement/Severity

CORT, Cortisol

DA, Dopamine

DDHF, Daily Diary of Hot Flashes

E2, Estradiol

EQ-5D, EuroQol 5-Dimension Health State

FSFI, Female Sexual Function Index

FSH, Follicle-Stimulating Hormone

GABA, Gamma-Aminobutyric Acid

GAD-7, Generalized Anxiety Disorder-7

GCS, Greene Climacteric Scale

Gly, Glycine

GnRH, Gonadotropin-Releasing Hormone

HAMA, Hamilton Anxiety Rating Scale

HAMD-17 / HAMD-24, Hamilton Depression Rating Scale (17-item / 24-item version)

HDL-C, High-Density Lipoprotein Cholesterol

IL-2 / IL-4 / IL-10, Interleukin-2 / -4 / -10

INF-γ, Interferon-Gamma

ISI, Insomnia Severity Index

KI, Kupperman Index

LDL-C, Low-Density Lipoprotein Cholesterol

LH, Luteinizing Hormone

MADRS, Montgomery-Åsberg Depression Rating Scale

Meno-D, Menopause Depression Rating Scale

MenQoL, Menopause-Specific Quality of Life Questionnaire

MRS, Menopause Rating Scale

MS-TSQ, Menopause Symptoms Treatment Satisfaction Questionnaire

NA / NE, Norepinephrine (Noradrenaline)

NPY, Neuropeptide Y

P, Progesterone

PAQ, Paffenbarger Activity Questionnaire

PRL, Prolactin

PSQI, Pittsburgh Sleep Quality Index

QIDS-SR, Quick Inventory of Depressive Symptomatology-Self-Report

SAS, Self-Rating Anxiety Scale

SCL-20 / SCL-90, Symptom Checklist-20 / -90

SCS, Self-Compassion Scale

SCSQ, Simplified Coping Style Questionnaire

SDS, Self-Rating Depression Scale

SERS, Rating Scale for Side Effects

SF-36, 36-Item Short Form Health Survey

SHBC, Sleep Hygiene Behavior Checklist

SRRS, Social Readjustment Rating Scale

SS, Somatostatin

STAI, State-Trait Anxiety Inventory

T, Testosterone

TC, Total Cholesterol

TG, Triglycerides

TNF-α, Tumor Necrosis Factor Alpha

VAS-PI, Visual Analog Scale – Pain Intensity

VIP, Vasoactive Intestinal Peptide

WHIIRS, Women's Health Initiative Insomnia Rating Scale
