# Supplementary material for: Pharmacotherapy, acupoint stimulation, and psychotherapy for perimenopausal women with anxiety, depression, and panic disorder: a systematic review and network meta-analysis of randomized controlled trials
Source: Front Psychiatry. 2026 Jul 17;17:1845876. doi: 10.3389/fpsyt.2026.1845876 (PMC13423873; doi:10.3389/fpsyt.2026.1845876)
Supplement: Supplementary file 1 [file Supplementaryfile1.zip › Manuscript_Supplementary_Figure_Table/Supplementary Material 4-global inconsistency results.docx]

# HAMD-ALL

## consistency

Results on the Mean Difference scale

Iterations = 20001:70000

Thinning interval = 1

Number of chains = 4

Sample size per chain = 50000

1. Empirical mean and standard deviation for each variable,

plus standard error of the mean:

Mean SD Naive SE Time-series SE

d.AcuStim.AcuStim_psych -5.417 4.2628 0.0095318 0.039891

d.AcuStim.control 4.617 0.7591 0.0016974 0.003279

d.AcuStim.drug 1.410 0.3646 0.0008152 0.001904

d.AcuStim.drug_AcuStim -1.577 0.5023 0.0011231 0.002504

d.drug.drug_psych -3.936 0.8023 0.0017940 0.002731

sd.d 1.500 0.2087 0.0004667 0.001167

2. Quantiles for each variable:

2.5% 25% 50% 75% 97.5%

d.AcuStim.AcuStim_psych -13.7970 -8.302 -5.422 -2.530 2.9379

d.AcuStim.control 3.1365 4.108 4.614 5.120 6.1189

d.AcuStim.drug 0.6988 1.167 1.407 1.652 2.1324

d.AcuStim.drug_AcuStim -2.5693 -1.911 -1.576 -1.242 -0.5943

d.drug.drug_psych -5.5220 -4.469 -3.931 -3.399 -2.3664

sd.d 1.1340 1.353 1.485 1.630 1.9502

-- Model fit (residual deviance):

Dbar pD DIC

112.34925 96.83119 209.18044

113 data points, ratio 0.9942, I^2 = 0.3%

## ume

Results on the Mean Difference scale

Iterations = 20001:70000

Thinning interval = 1

Number of chains = 4

Sample size per chain = 50000

1. Empirical mean and standard deviation for each variable,

plus standard error of the mean:

Mean SD Naive SE Time-series SE

d.AcuStim.AcuStim_psych -5.3923 4.2065 0.0094061 0.041856

d.AcuStim.control 0.3862 1.6232 0.0036296 0.005565

d.AcuStim.drug 1.5796 0.3549 0.0007935 0.001398

d.AcuStim.drug_AcuStim -1.2594 0.8520 0.0019051 0.003003

d.control.drug -4.0328 0.7179 0.0016053 0.002946

d.drug.drug_AcuStim -3.0156 0.3912 0.0008748 0.001432

d.drug.drug_psych -3.9225 0.7498 0.0016767 0.002640

sd.d 1.3715 0.2044 0.0004570 0.001227

2. Quantiles for each variable:

2.5% 25% 50% 75% 97.5%

d.AcuStim.AcuStim_psych -13.6130 -8.2313 -5.3976 -2.5586 2.8931

d.AcuStim.control -2.8221 -0.6876 0.3869 1.4663 3.5745

d.AcuStim.drug 0.8902 1.3427 1.5769 1.8149 2.2850

d.AcuStim.drug_AcuStim -2.9571 -1.8226 -1.2526 -0.6907 0.3974

d.control.drug -5.4451 -4.5107 -4.0340 -3.5547 -2.6134

d.drug.drug_AcuStim -3.7928 -3.2745 -3.0134 -2.7564 -2.2460

d.drug.drug_psych -5.4031 -4.4233 -3.9201 -3.4215 -2.4494

sd.d 1.0128 1.2281 1.3570 1.4991 1.8105

-- Model fit (residual deviance):

Dbar pD DIC

112.30043 95.55304 207.85347

113 data points, ratio 0.9938, I^2 = 0.3%

# HAMD-drug

## consistency

Results on the Mean Difference scale

Iterations = 20001:70000

Thinning interval = 1

Number of chains = 4

Sample size per chain = 50000

1. Empirical mean and standard deviation for each variable,

plus standard error of the mean:

Mean SD

d.control.ADs -7.06378 3.4706

d.control.HRT 0.04224 2.3556

d.control.SNRI -4.22549 2.0347

d.control.SSRI -3.75644 2.1890

d.control.TCM -3.66700 3.4652

d.SSRI.ADs_AP -2.41406 1.9654

d.SSRI.ADs_HRT -1.34621 1.4111

d.SSRI.ADs_TCM -3.79644 1.5597

d.SSRI.delexin 0.60644 1.9555

d.SSRI.TCA 2.68736 2.0229

sd.d 3.11649 0.5877

Naive SE

d.control.ADs 0.007761

d.control.HRT 0.005267

d.control.SNRI 0.004550

d.control.SSRI 0.004895

d.control.TCM 0.007748

d.SSRI.ADs_AP 0.004395

d.SSRI.ADs_HRT 0.003155

d.SSRI.ADs_TCM 0.003488

d.SSRI.delexin 0.004373

d.SSRI.TCA 0.004523

sd.d 0.001314

Time-series SE

d.control.ADs 0.012133

d.control.HRT 0.016819

d.control.SNRI 0.007436

d.control.SSRI 0.015332

d.control.TCM 0.011583

d.SSRI.ADs_AP 0.005623

d.SSRI.ADs_HRT 0.006348

d.SSRI.ADs_TCM 0.004461

d.SSRI.delexin 0.006057

d.SSRI.TCA 0.007790

sd.d 0.003159

2. Quantiles for each variable:

2.5% 25%

d.control.ADs -13.903 -9.3435

d.control.HRT -4.666 -1.4922

d.control.SNRI -8.269 -5.5478

d.control.SSRI -8.135 -5.1793

d.control.TCM -10.510 -5.9349

d.SSRI.ADs_AP -6.315 -3.6951

d.SSRI.ADs_HRT -4.133 -2.2718

d.SSRI.ADs_TCM -6.872 -4.8126

d.SSRI.delexin -3.235 -0.6686

d.SSRI.TCA -1.334 1.3703

sd.d 2.161 2.7031

50% 75%

d.control.ADs -7.06989 -4.7915

d.control.HRT 0.06165 1.5942

d.control.SNRI -4.22128 -2.9021

d.control.SSRI -3.73668 -2.3198

d.control.TCM -3.67936 -1.3957

d.SSRI.ADs_AP -2.41290 -1.1262

d.SSRI.ADs_HRT -1.35132 -0.4285

d.SSRI.ADs_TCM -3.80160 -2.7828

d.SSRI.delexin 0.59993 1.8760

d.SSRI.TCA 2.68153 4.0084

sd.d 3.05017 3.4554

97.5%

d.control.ADs -0.1879

d.control.HRT 4.6448

d.control.SNRI -0.1954

d.control.SSRI 0.5236

d.control.TCM 3.2116

d.SSRI.ADs_AP 1.4664

d.SSRI.ADs_HRT 1.4469

d.SSRI.ADs_TCM -0.6907

d.SSRI.delexin 4.4991

d.SSRI.TCA 6.6713

sd.d 4.4542

-- Model fit (residual deviance):

Dbar pD DIC

61.35200 57.56115 118.91315

61 data points, ratio 1.006, I^2 = 2%

## ume

Results on the Mean Difference scale

Iterations = 20001:70000

Thinning interval = 1

Number of chains = 4

Sample size per chain = 50000

1. Empirical mean and standard deviation for each variable,

plus standard error of the mean:

Mean SD Naive SE Time-series SE

d.ADs.control 7.0936 3.6322 0.008122 0.012482

d.ADs.TCM 3.4191 3.5075 0.007843 0.011712

d.ADs_AP.SSRI 2.4139 2.0613 0.004609 0.005797

d.ADs_HRT.HRT 4.8316 1.3566 0.003034 0.004290

d.ADs_HRT.SSRI 1.4692 1.7142 0.003833 0.005451

d.ADs_HRT.TCA 4.4416 1.9777 0.004422 0.004884

d.ADs_TCM.delexin 4.3140 3.4613 0.007740 0.008640

d.ADs_TCM.SSRI 3.8012 1.7733 0.003965 0.004790

d.control.HRT -3.6181 3.9883 0.008918 0.013014

d.control.SNRI -4.0543 2.4362 0.005448 0.006347

d.control.SSRI -1.4219 3.4978 0.007821 0.009057

d.delexin.HRT 6.5818 3.3380 0.007464 0.010396

d.delexin.SSRI -2.1957 2.5919 0.005796 0.008441

d.SNRI.SSRI 0.8351 3.4648 0.007748 0.008668

d.SSRI.TCA 1.0764 3.8236 0.008550 0.011839

sd.d 3.2681 0.6902 0.001543 0.004140

2. Quantiles for each variable:

2.5% 25% 50% 75% 97.5%

d.ADs.control -0.11601 4.7476 7.0903 9.4453 14.3102

d.ADs.TCM -3.54295 1.1516 3.4215 5.6652 10.3726

d.ADs_AP.SSRI -1.65621 1.0791 2.4085 3.7427 6.5049

d.ADs_HRT.HRT 2.12080 3.9565 4.8397 5.7152 7.4884

d.ADs_HRT.SSRI -1.94241 0.3601 1.4752 2.5898 4.8319

d.ADs_HRT.TCA 0.49100 3.1736 4.4406 5.7174 8.3745

d.ADs_TCM.delexin -2.54484 2.0907 4.3133 6.5321 11.1920

d.ADs_TCM.SSRI 0.25533 2.6577 3.8112 4.9511 7.2859

d.control.HRT -11.50005 -6.2428 -3.6263 -0.9933 4.2274

d.control.SNRI -8.88453 -5.6240 -4.0583 -2.4852 0.7814

d.control.SSRI -8.33983 -3.6782 -1.4333 0.8231 5.5356

d.delexin.HRT -0.01732 4.4227 6.5790 8.7328 13.2096

d.delexin.SSRI -7.32889 -3.8779 -2.1953 -0.5098 2.9228

d.SNRI.SSRI -6.05792 -1.4021 0.8402 3.0774 7.6996

d.SSRI.TCA -6.48399 -1.4241 1.0825 3.5738 8.6437

sd.d 2.18457 2.7838 3.1783 3.6536 4.8686

-- Model fit (residual deviance):

Dbar pD DIC

60.97276 58.63997 119.61274

61 data points, ratio 0.9996, I^2 = 2%

# HAMA-ALL

## consistency

Results on the Mean Difference scale

Iterations = 20001:70000

Thinning interval = 1

Number of chains = 4

Sample size per chain = 50000

1. Empirical mean and standard deviation for each variable,

plus standard error of the mean:

Mean SD Naive SE Time-series SE

d.drug.AcuStim -2.7496 1.423 0.003183 0.005694

d.drug.AcuStim_psych -3.8763 1.533 0.003428 0.004332

d.drug.control 0.9433 1.691 0.003781 0.006675

d.drug.drug_AcuStim -3.9201 0.955 0.002135 0.002427

d.drug.drug_psych -2.3471 1.487 0.003326 0.003942

sd.d 2.3726 0.587 0.001313 0.003054

2. Quantiles for each variable:

2.5% 25% 50% 75% 97.5%

d.drug.AcuStim -5.555 -3.6639 -2.7500 -1.838 0.0830

d.drug.AcuStim_psych -6.970 -4.8396 -3.8630 -2.899 -0.8508

d.drug.control -2.420 -0.1339 0.9443 2.028 4.2923

d.drug.drug_AcuStim -5.828 -4.5218 -3.9181 -3.314 -2.0265

d.drug.drug_psych -5.333 -3.2875 -2.3374 -1.395 0.5814

sd.d 1.483 1.9611 2.2861 2.685 3.7671

-- Model fit (residual deviance):

Dbar pD DIC

38.58724 36.60032 75.18756

38 data points, ratio 1.015, I^2 = 4%

## ume

Results on the Mean Difference scale

Iterations = 20001:70000

Thinning interval = 1

Number of chains = 4

Sample size per chain = 50000

1. Empirical mean and standard deviation for each variable,

plus standard error of the mean:

Mean SD Naive SE Time-series SE

d.AcuStim.AcuStim_psych -3.394 2.5944 0.005801 0.008402

d.AcuStim.control 2.993 1.4916 0.003335 0.004566

d.AcuStim.drug 4.835 1.7722 0.003963 0.004987

d.AcuStim_psych.drug 2.963 1.6448 0.003678 0.003924

d.control.drug -2.737 2.4131 0.005396 0.006825

d.drug.drug_AcuStim -3.914 0.9000 0.002012 0.002335

d.drug.drug_psych -2.323 1.4073 0.003147 0.003845

sd.d 2.205 0.6217 0.001390 0.003747

2. Quantiles for each variable:

2.5% 25% 50% 75% 97.5%

d.AcuStim.AcuStim_psych -8.54736 -5.041 -3.392 -1.751 1.7660

d.AcuStim.control 0.02396 2.045 2.993 3.940 5.9584

d.AcuStim.drug 1.29021 3.716 4.842 5.967 8.3285

d.AcuStim_psych.drug -0.32653 1.941 2.959 3.979 6.2724

d.control.drug -7.57273 -4.247 -2.738 -1.222 2.0717

d.drug.drug_AcuStim -5.71682 -4.474 -3.912 -3.353 -2.1194

d.drug.drug_psych -5.17124 -3.194 -2.304 -1.434 0.4396

sd.d 1.28487 1.773 2.108 2.526 3.6935

-- Model fit (residual deviance):

Dbar pD DIC

38.81369 36.73083 75.54452

38 data points, ratio 1.021, I^2 = 5%

# HAMA-drug

## consistency

Results on the Mean Difference scale

Iterations = 20001:70000

Thinning interval = 1

Number of chains = 4

Sample size per chain = 50000

1. Empirical mean and standard deviation for each variable,

plus standard error of the mean:

Mean SD Naive SE Time-series SE

d.ADs_HRT.TCA 3.9165 4.524 0.010117 0.011832

d.SSRI.ADs_AP -3.5974 2.588 0.005786 0.006438

d.SSRI.ADs_HRT 0.2485 3.671 0.008209 0.014415

d.SSRI.control 6.2152 3.411 0.007628 0.011074

d.SSRI.HRT 5.1968 3.011 0.006732 0.011621

d.SSRI.SNRI 1.4159 3.576 0.007997 0.011024

d.SSRI.TCM -0.8786 4.234 0.009468 0.013964

d.TCM.BZD 1.0695 4.347 0.009721 0.009778

sd.d 4.0503 1.585 0.003545 0.011306

2. Quantiles for each variable:

2.5% 25% 50% 75% 97.5%

d.ADs_HRT.TCA -5.3310 1.2637 3.9004 6.569 13.102

d.SSRI.ADs_AP -8.9281 -5.0881 -3.5834 -2.089 1.644

d.SSRI.ADs_HRT -7.2609 -1.8755 0.2607 2.390 7.698

d.SSRI.control -0.8445 4.2858 6.2572 8.174 13.107

d.SSRI.HRT -1.0985 3.4776 5.2555 6.968 11.214

d.SSRI.SNRI -5.9071 -0.6295 1.4424 3.477 8.682

d.SSRI.TCM -9.4815 -3.3457 -0.8577 1.601 7.679

d.TCM.BZD -7.8291 -1.3857 1.0627 3.527 10.014

sd.d 1.8721 2.8822 3.7097 4.883 8.108

-- Model fit (residual deviance):

Dbar pD DIC

23.08086 22.74552 45.82638

23 data points, ratio 1.004, I^2 = 5%

## ume

Results on the Mean Difference scale

Iterations = 20001:70000

Thinning interval = 1

Number of chains = 4

Sample size per chain = 50000

1. Empirical mean and standard deviation for each variable,

plus standard error of the mean:

Mean SD Naive SE Time-series SE

d.ADs_AP.SSRI 3.5643 2.426 0.005426 0.006193

d.ADs_HRT.HRT 4.3597 2.987 0.006679 0.010006

d.ADs_HRT.SSRI 1.7451 4.044 0.009042 0.013954

d.ADs_HRT.TCA 3.9172 4.258 0.009521 0.011460

d.ADs_HRT.TCM -0.6679 4.004 0.008954 0.013883

d.BZD.TCM -1.0686 4.066 0.009091 0.009160

d.control.HRT -0.6984 4.057 0.009073 0.011753

d.control.SNRI -2.7327 4.128 0.009231 0.010118

d.control.SSRI -9.4907 4.069 0.009099 0.011886

d.SNRI.SSRI 0.6969 4.138 0.009253 0.010016

sd.d 3.5674 1.932 0.004321 0.019772

2. Quantiles for each variable:

2.5% 25% 50% 75% 97.5%

d.ADs_AP.SSRI -1.424 2.2978 3.5156 4.8067 8.7317

d.ADs_HRT.HRT -2.071 2.8376 4.4800 5.9557 10.3763

d.ADs_HRT.SSRI -6.752 -0.4119 1.8041 3.9270 10.0511

d.ADs_HRT.TCA -5.008 1.6860 3.9152 6.1473 12.8217

d.ADs_HRT.TCM -9.101 -2.7753 -0.6110 1.4585 7.6248

d.BZD.TCM -9.678 -3.0565 -1.0715 0.9076 7.5593

d.control.HRT -9.291 -2.6772 -0.7003 1.2858 7.8999

d.control.SNRI -11.462 -4.7961 -2.7389 -0.6639 5.9421

d.control.SSRI -18.136 -11.4849 -9.5012 -7.5097 -0.8157

d.SNRI.SSRI -8.004 -1.3994 0.6964 2.7937 9.4138

sd.d 0.985 2.1271 3.0942 4.6016 8.4192

-- Model fit (residual deviance):

Dbar pD DIC

23.20301 22.83750 46.04052

23 data points, ratio 1.009, I^2 = 5%

# clinical efficacy-all

## ume

Results on the Log Odds Ratio scale

Iterations = 20001:70000

Thinning interval = 1

Number of chains = 4

Sample size per chain = 50000

1. Empirical mean and standard deviation for each variable,

plus standard error of the mean:

Mean SD Naive SE Time-series SE

d.AcuStim.control -0.4646 0.9770 0.0021846 0.004198

d.AcuStim.drug -0.9202 0.2660 0.0005947 0.001590

d.AcuStim.drug_AcuStim 1.3598 0.4882 0.0010916 0.002389

d.drug.drug_AcuStim 1.0535 0.2528 0.0005653 0.001184

d.drug.drug_psych 0.4478 0.6982 0.0015611 0.002820

d.drug.psych 0.1531 0.7705 0.0017229 0.003744

d.drug_psych.psych -0.2857 1.1003 0.0024604 0.005866

sd.d 0.7800 0.1757 0.0003929 0.001721

2. Quantiles for each variable:

2.5% 25% 50% 75% 97.5%

d.AcuStim.control -2.4034 -1.10534 -0.4643 0.1804 1.4620

d.AcuStim.drug -1.4587 -1.09279 -0.9154 -0.7413 -0.4107

d.AcuStim.drug_AcuStim 0.4210 1.03316 1.3511 1.6764 2.3483

d.drug.drug_AcuStim 0.5676 0.88370 1.0496 1.2181 1.5624

d.drug.drug_psych -0.9238 -0.01297 0.4441 0.9033 1.8397

d.drug.psych -1.3375 -0.36133 0.1433 0.6530 1.7039

d.drug_psych.psych -2.4737 -1.00536 -0.2804 0.4443 1.8589

sd.d 0.4620 0.65970 0.7698 0.8904 1.1548

-- Model fit (residual deviance):

Dbar pD DIC

90.42911 73.31599 163.74510

94 data points, ratio 0.962, I^2 = 0%

## consistency

Results on the Log Odds Ratio scale

Iterations = 20001:70000

Thinning interval = 1

Number of chains = 4

Sample size per chain = 50000

1. Empirical mean and standard deviation for each variable,

plus standard error of the mean:

Mean SD Naive SE Time-series SE

d.AcuStim.control -0.4768 1.0044 0.0022458 0.004292

d.AcuStim.drug -0.7202 0.2494 0.0005577 0.001665

d.AcuStim.drug_AcuStim 0.5742 0.3040 0.0006799 0.001871

d.drug.drug_psych 0.4504 0.6331 0.0014156 0.002791

d.drug.psych 0.1675 0.6771 0.0015141 0.003367

sd.d 0.8115 0.1727 0.0003861 0.001598

2. Quantiles for each variable:

2.5% 25% 50% 75% 97.5%

d.AcuStim.control -2.46520 -1.13591 -0.4753 0.1855 1.4992

d.AcuStim.drug -1.22219 -0.88269 -0.7165 -0.5536 -0.2386

d.AcuStim.drug_AcuStim -0.02384 0.37259 0.5729 0.7744 1.1791

d.drug.drug_psych -0.78660 0.03102 0.4471 0.8637 1.7144

d.drug.psych -1.14931 -0.28314 0.1593 0.6099 1.5275

sd.d 0.49999 0.69249 0.8017 0.9197 1.1777

-- Model fit (residual deviance):

Dbar pD DIC

91.05471 73.12942 164.18413

94 data points, ratio 0.9687, I^2 = 0%

# clinical efficacy-drug

## ume

Results on the Log Odds Ratio scale

Iterations = 20001:70000

Thinning interval = 1

Number of chains = 4

Sample size per chain = 50000

1. Empirical mean and standard deviation for each variable,

plus standard error of the mean:

Mean SD Naive SE Time-series SE

d.ADs_AP.SSRI 0.3771 1.0115 0.002262 0.005259

d.ADs_HRT.ADs_HRT_TCM -1.7988 1.3290 0.002972 0.007555

d.ADs_HRT.HRT -2.3759 0.8285 0.001853 0.007723

d.ADs_HRT.SSRI -1.7754 0.9583 0.002143 0.008613

d.ADs_HRT.TCA -0.5345 0.7439 0.001664 0.003394

d.ADs_TCM.delexin -2.4364 1.7421 0.003896 0.020681

d.ADs_TCM.oryzanol -3.2179 1.7660 0.003949 0.023337

d.ADs_TCM.SSRI -1.7577 0.7208 0.001612 0.005501

d.delexin.SSRI -0.9321 1.4886 0.003329 0.009658

sd.d 0.9941 0.4815 0.001077 0.006711

2. Quantiles for each variable:

2.5% 25% 50% 75% 97.5%

d.ADs_AP.SSRI -1.7240 -0.2280 0.4048 1.008934 2.32392

d.ADs_HRT.ADs_HRT_TCM -4.5165 -2.5940 -1.7784 -0.993943 0.84472

d.ADs_HRT.HRT -4.0812 -2.8788 -2.3554 -1.855874 -0.77451

d.ADs_HRT.SSRI -3.7594 -2.3482 -1.7600 -1.184387 0.09663

d.ADs_HRT.TCA -2.0634 -0.9733 -0.5238 -0.090138 0.94745

d.ADs_TCM.delexin -6.1714 -3.4756 -2.3371 -1.290192 0.72770

d.ADs_TCM.oryzanol -7.1261 -4.2301 -3.0914 -2.066262 -0.06579

d.ADs_TCM.SSRI -3.2684 -2.1920 -1.7326 -1.293244 -0.38794

d.delexin.SSRI -3.9698 -1.8467 -0.9155 0.009257 2.00075

sd.d 0.1692 0.6663 0.9383 1.260454 2.12995

-- Model fit (residual deviance):

Dbar pD DIC

36.51816 32.48617 69.00433

36 data points, ratio 1.014, I^2 = 4%

## consistency

Results on the Log Odds Ratio scale

Iterations = 20001:70000

Thinning interval = 1

Number of chains = 4

Sample size per chain = 50000

1. Empirical mean and standard deviation for each variable,

plus standard error of the mean:

Mean SD Naive SE Time-series SE

d.ADs_HRT.ADs_HRT_TCM -1.8008 1.2723 0.002845 0.007458

d.ADs_HRT.TCA -0.5325 0.7077 0.001582 0.003225

d.ADs_TCM.oryzanol -3.1740 1.6628 0.003718 0.020750

d.SSRI.ADs_AP -0.3905 0.9766 0.002184 0.005312

d.SSRI.ADs_HRT 1.7876 0.9298 0.002079 0.008807

d.SSRI.ADs_TCM 1.8868 0.6676 0.001493 0.005340

d.SSRI.delexin 0.3149 1.0650 0.002381 0.007494

d.SSRI.HRT -0.5986 0.8058 0.001802 0.003608

sd.d 0.9378 0.4486 0.001003 0.006537

2. Quantiles for each variable:

2.5% 25% 50% 75% 97.5%

d.ADs_HRT.ADs_HRT_TCM -4.39788 -2.5655 -1.7839 -1.0128 0.7128

d.ADs_HRT.TCA -1.99053 -0.9531 -0.5230 -0.1063 0.8751

d.ADs_TCM.oryzanol -6.77816 -4.1626 -3.0666 -2.0755 -0.1720

d.SSRI.ADs_AP -2.27732 -1.0085 -0.4153 0.2022 1.6313

d.SSRI.ADs_HRT -0.03036 1.2077 1.7680 2.3534 3.6884

d.SSRI.ADs_TCM 0.62235 1.4545 1.8613 2.2927 3.2775

d.SSRI.delexin -1.82120 -0.3497 0.3220 0.9845 2.4330

d.SSRI.HRT -2.23227 -1.0701 -0.5999 -0.1273 1.0331

sd.d 0.17798 0.6327 0.8862 1.1874 1.9909

-- Model fit (residual deviance):

Dbar pD DIC

36.0630 31.4522 67.5152

36 data points, ratio 1.002, I^2 = 3%

# AE-all

## ume

Results on the Log Odds Ratio scale

Iterations = 20001:70000

Thinning interval = 1

Number of chains = 4

Sample size per chain = 50000

1. Empirical mean and standard deviation for each variable,

plus standard error of the mean:

Mean SD Naive SE Time-series SE

d.AcuStim.control -0.88321 1.5545 0.0034759 0.005547

d.AcuStim.drug 0.79187 0.5121 0.0011451 0.002404

d.AcuStim.drug_AcuStim -0.01223 0.8949 0.0020011 0.003893

d.control.drug 1.00338 1.3989 0.0031280 0.003710

d.drug.drug_AcuStim -0.63895 0.5272 0.0011787 0.001973

sd.d 1.29158 0.3371 0.0007538 0.002568

2. Quantiles for each variable:

2.5% 25% 50% 75% 97.5%

d.AcuStim.control -4.0000 -1.8836 -0.876930 0.1239 2.1722

d.AcuStim.drug -0.1962 0.4574 0.782568 1.1165 1.8334

d.AcuStim.drug_AcuStim -1.8112 -0.5890 -0.004549 0.5739 1.7368

d.control.drug -1.7880 0.1252 1.002289 1.8836 3.7890

d.drug.drug_AcuStim -1.6936 -0.9771 -0.634776 -0.2989 0.3996

sd.d 0.7488 1.0517 1.249587 1.4859 2.0734

-- Model fit (residual deviance):

Dbar pD DIC

44.02904 38.31461 82.34365

44 data points, ratio 1.001, I^2 = 2%

## consistency

Results on the Log Odds Ratio scale

Iterations = 20001:70000

Thinning interval = 1

Number of chains = 4

Sample size per chain = 50000

1. Empirical mean and standard deviation for each variable,

plus standard error of the mean:

Mean SD Naive SE Time-series SE

d.AcuStim.control -0.54411 0.9873 0.0022077 0.003451

d.AcuStim.drug 0.72055 0.4431 0.0009908 0.002573

d.AcuStim.drug_AcuStim 0.06616 0.5595 0.0012512 0.003154

sd.d 1.17588 0.3012 0.0006735 0.002227

2. Quantiles for each variable:

2.5% 25% 50% 75% 97.5%

d.AcuStim.control -2.5089 -1.1777 -0.54284 0.08737 1.415

d.AcuStim.drug -0.1397 0.4309 0.71423 1.00341 1.615

d.AcuStim.drug_AcuStim -1.0375 -0.2975 0.06513 0.43113 1.175

sd.d 0.6850 0.9643 1.14199 1.35005 1.864

-- Model fit (residual deviance):

Dbar pD DIC

43.90192 36.98683 80.88875

44 data points, ratio 0.9978, I^2 = 2%

# AE-drug

## ume

Results on the Log Odds Ratio scale

Iterations = 20001:70000

Thinning interval = 1

Number of chains = 4

Sample size per chain = 50000

1. Empirical mean and standard deviation for each variable,

plus standard error of the mean:

Mean SD Naive SE Time-series SE

d.ADs_HRT.TCA 0.1410 2.110 0.004719 0.005236

d.ADs_TCM.delexin 0.5377 3.103 0.006939 0.010201

d.ADs_TCM.SSRI 2.0071 1.907 0.004263 0.022908

d.control.SNRI 1.0144 2.962 0.006624 0.006844

d.delexin.SSRI 1.2670 2.990 0.006687 0.007983

d.SNRI.SSRI -0.1513 2.977 0.006658 0.007229

d.SSRI.TCA 66.0490 45.971 0.102794 2.925035

sd.d 2.6317 1.285 0.002874 0.020875

2. Quantiles for each variable:

2.5% 25% 50% 75% 97.5%

d.ADs_HRT.TCA -4.3505 -0.9518 0.1415 1.237 4.598

d.ADs_TCM.delexin -5.9493 -1.1751 0.5143 2.244 7.057

d.ADs_TCM.SSRI -1.5119 0.8833 1.8102 2.976 6.386

d.control.SNRI -5.2873 -0.5036 1.0081 2.531 7.318

d.delexin.SSRI -5.0720 -0.2978 1.2606 2.840 7.575

d.SNRI.SSRI -6.4718 -1.6987 -0.1511 1.410 6.165

d.SSRI.TCA 7.1158 29.3809 56.2886 93.412 171.680

sd.d 0.6137 1.5929 2.4623 3.588 5.194

-- Model fit (residual deviance):

Dbar pD DIC

19.75519 18.42752 38.18271

20 data points, ratio 0.9878, I^2 = 4%

## consistency

Results on the Log Odds Ratio scale

Iterations = 20001:70000

Thinning interval = 1

Number of chains = 4

Sample size per chain = 50000

1. Empirical mean and standard deviation for each variable,

plus standard error of the mean:

Mean SD Naive SE Time-series SE

d.SNRI.control -1.0099 2.406 0.005380 0.005721

d.SSRI.ADs_TCM -1.8084 1.446 0.003232 0.016238

d.SSRI.delexin -1.2903 1.903 0.004256 0.008163

d.SSRI.SNRI 0.1532 2.432 0.005438 0.006110

d.SSRI.TCA 65.4208 45.573 0.101904 3.724849

d.TCA.ADs_HRT -0.1472 1.719 0.003844 0.004471

sd.d 2.0516 1.183 0.002646 0.020676

2. Quantiles for each variable:

2.5% 25% 50% 75% 97.5%

d.SNRI.control -6.152 -2.1549 -1.0037 0.1445 4.1039

d.SSRI.ADs_TCM -5.206 -2.4950 -1.6498 -0.9682 0.7769

d.SSRI.delexin -5.484 -2.2273 -1.2217 -0.2891 2.5165

d.SSRI.SNRI -5.001 -1.0456 0.1493 1.3438 5.3561

d.SSRI.TCA 7.246 29.3098 56.6943 90.4375 174.1614

d.TCA.ADs_HRT -3.799 -0.9887 -0.1430 0.7052 3.4750

sd.d 0.374 1.1532 1.7922 2.7427 4.8903

-- Model fit (residual deviance):

Dbar pD DIC

20.03640 18.10301 38.13941

20 data points, ratio 1.002, I^2 = 5%

# KI

## consistency

Results on the Mean Difference scale

Iterations = 20001:70000

Thinning interval = 1

Number of chains = 4

Sample size per chain = 50000

1. Empirical mean and standard deviation for each variable,

plus standard error of the mean:

Mean SD Naive SE Time-series SE

d.drug.AcuStim 1.783 1.0861 0.002429 0.003369

d.drug.control 5.268 1.6633 0.003719 0.005554

d.drug.drug_AcuStim -4.148 0.7307 0.001634 0.002106

d.drug.drug_psych -6.903 2.1248 0.004751 0.006047

sd.d 2.808 0.4941 0.001105 0.002203

2. Quantiles for each variable:

2.5% 25% 50% 75% 97.5%

d.drug.AcuStim -0.3686 1.073 1.783 2.490 3.940

d.drug.control 2.0341 4.171 5.246 6.351 8.613

d.drug.drug_AcuStim -5.5944 -4.625 -4.147 -3.670 -2.706

d.drug.drug_psych -11.1108 -8.293 -6.896 -5.516 -2.699

sd.d 2.0104 2.458 2.751 3.093 3.936

-- Model fit (residual deviance):

Dbar pD DIC

56.01632 52.63230 108.64862

55 data points, ratio 1.018, I^2 = 4%

## ume

Results on the Mean Difference scale

Iterations = 20001:70000

Thinning interval = 1

Number of chains = 4

Sample size per chain = 50000

1. Empirical mean and standard deviation for each variable,

plus standard error of the mean:

Mean SD Naive SE Time-series SE

d.AcuStim.control 2.0744 2.1292 0.004761 0.005794

d.AcuStim.drug -0.1886 1.4536 0.003250 0.003823

d.AcuStim.drug_AcuStim -6.7156 1.4209 0.003177 0.003576

d.control.drug -6.8771 2.2870 0.005114 0.007657

d.drug.drug_AcuStim -3.8117 0.7976 0.001783 0.002103

d.drug.drug_psych -6.9015 2.1203 0.004741 0.005987

sd.d 2.7989 0.5045 0.001128 0.002301

2. Quantiles for each variable:

2.5% 25% 50% 75% 97.5%

d.AcuStim.control -2.116 0.6755 2.0674 3.4683 6.301

d.AcuStim.drug -3.050 -1.1399 -0.1891 0.7543 2.694

d.AcuStim.drug_AcuStim -9.541 -7.6351 -6.7099 -5.7945 -3.907

d.control.drug -11.503 -8.3641 -6.8323 -5.3543 -2.487

d.drug.drug_AcuStim -5.387 -4.3346 -3.8113 -3.2907 -2.240

d.drug.drug_psych -11.121 -8.2826 -6.8887 -5.5184 -2.712

sd.d 1.988 2.4403 2.7401 3.0889 3.954

-- Model fit (residual deviance):

Dbar pD DIC

55.1583 52.8177 107.9760

55 data points, ratio 1.003, I^2 = 2%

# SDS

## consistency

Results on the Mean Difference scale

Iterations = 20001:70000

Thinning interval = 1

Number of chains = 4

Sample size per chain = 50000

1. Empirical mean and standard deviation for each variable,

plus standard error of the mean:

Mean SD Naive SE Time-series SE

d.AcuStim.control 7.013 2.885 0.006451 0.017179

d.AcuStim.drug 1.411 2.680 0.005993 0.019589

d.AcuStim.drug_AcuStim -5.281 3.010 0.006730 0.020541

d.AcuStim.drug_psych -7.225 2.960 0.006619 0.017760

d.drug_psych.psych 15.602 4.019 0.008987 0.010100

sd.d 3.700 1.199 0.002681 0.007729

2. Quantiles for each variable:

2.5% 25% 50% 75% 97.5%

d.AcuStim.control 1.303 5.2219 7.000 8.800 12.7962

d.AcuStim.drug -3.914 -0.2601 1.424 3.090 6.7304

d.AcuStim.drug_AcuStim -11.327 -7.1529 -5.250 -3.379 0.6385

d.AcuStim.drug_psych -13.126 -9.0683 -7.212 -5.363 -1.3760

d.drug_psych.psych 7.557 13.1572 15.591 18.050 23.6860

sd.d 1.965 2.8760 3.505 4.296 6.5920

-- Model fit (residual deviance):

Dbar pD DIC

29.03437 27.28311 56.31748

29 data points, ratio 1.001, I^2 = 4%

## ume

Results on the Mean Difference scale

Iterations = 20001:70000

Thinning interval = 1

Number of chains = 4

Sample size per chain = 50000

1. Empirical mean and standard deviation for each variable,

plus standard error of the mean:

Mean SD Naive SE Time-series SE

d.AcuStim.control 7.570 4.539 0.010149 0.012341

d.AcuStim.drug 3.233 5.050 0.011292 0.025953

d.AcuStim.drug_AcuStim -8.691 4.906 0.010969 0.024905

d.AcuStim.drug_psych -6.695 4.761 0.010647 0.013892

d.control.drug -5.424 2.612 0.005840 0.006737

d.drug.drug_AcuStim -5.321 2.317 0.005180 0.006521

d.drug.drug_psych -8.812 2.683 0.005999 0.007623

d.drug_psych.psych 15.611 4.428 0.009900 0.011085

sd.d 3.997 1.637 0.003660 0.012746

2. Quantiles for each variable:

2.5% 25% 50% 75% 97.5%

d.AcuStim.control -1.604 4.9252 7.575 10.230 16.7088

d.AcuStim.drug -6.789 0.1124 3.241 6.335 13.2889

d.AcuStim.drug_AcuStim -18.438 -11.6826 -8.680 -5.717 1.0583

d.AcuStim.drug_psych -16.176 -9.5515 -6.698 -3.841 2.7889

d.control.drug -10.758 -6.9249 -5.399 -3.904 -0.2322

d.drug.drug_AcuStim -10.033 -6.6756 -5.286 -3.942 -0.7268

d.drug.drug_psych -14.207 -10.3942 -8.814 -7.221 -3.4386

d.drug_psych.psych 6.734 13.0518 15.600 18.154 24.6026

sd.d 1.794 2.8776 3.674 4.740 8.1593

-- Model fit (residual deviance):

Dbar pD DIC

29.33132 28.11626 57.44758

29 data points, ratio 1.011, I^2 = 5%

# SAS

## consistency

Results on the Mean Difference scale

Iterations = 20001:70000

Thinning interval = 1

Number of chains = 4

Sample size per chain = 50000

1. Empirical mean and standard deviation for each variable,

plus standard error of the mean:

Mean SD Naive SE Time-series SE

d.drug.AcuStim -3.08178 6.177 0.013813 0.020937

d.drug.control 5.70731 6.871 0.015364 0.021607

d.drug.drug_AcuStim -6.83575 3.454 0.007724 0.009894

d.drug.drug_psych -8.30347 4.055 0.009066 0.011447

d.drug.psych 0.00959 4.184 0.009356 0.012086

sd.d 5.96675 2.335 0.005220 0.014039

2. Quantiles for each variable:

2.5% 25% 50% 75% 97.5%

d.drug.AcuStim -15.556 -6.716 -3.08311 0.5481 9.39499

d.drug.control -8.181 1.636 5.69248 9.7788 19.56650

d.drug.drug_AcuStim -13.870 -8.834 -6.83961 -4.8539 0.21494

d.drug.drug_psych -16.486 -10.672 -8.33403 -5.9581 -0.01662

d.drug.psych -8.504 -2.408 0.01358 2.3980 8.56799

sd.d 2.852 4.266 5.43340 7.1257 12.07929

-- Model fit (residual deviance):

Dbar pD DIC

18.96208 18.73302 37.69510

19 data points, ratio 0.998, I^2 = 5%

## ume

Results on the Mean Difference scale

Iterations = 20001:70000

Thinning interval = 1

Number of chains = 4

Sample size per chain = 50000

1. Empirical mean and standard deviation for each variable,

plus standard error of the mean:

Mean SD Naive SE Time-series SE

d.AcuStim.drug 3.118 4.835 0.010810 0.028038

d.AcuStim.drug_AcuStim -3.805 4.775 0.010678 0.026198

d.control.drug -5.679 4.930 0.011024 0.025863

d.drug.drug_AcuStim -6.404 3.107 0.006947 0.008617

d.drug.drug_psych -4.898 3.230 0.007223 0.011084

d.drug.psych -6.624 4.387 0.009810 0.012388

d.drug_AcuStim.psych 7.383 4.403 0.009844 0.013427

d.drug_psych.psych 14.387 4.369 0.009770 0.012054

sd.d 3.057 2.946 0.006588 0.050649

2. Quantiles for each variable:

2.5% 25% 50% 75% 97.5%

d.AcuStim.drug -6.7446 0.7742 3.103 5.438 13.1211

d.AcuStim.drug_AcuStim -13.7352 -6.0434 -3.795 -1.594 6.1374

d.control.drug -15.7648 -8.1296 -5.660 -3.228 4.2898

d.drug.drug_AcuStim -13.2233 -7.5162 -6.310 -5.238 0.1077

d.drug.drug_psych -11.8126 -6.2322 -4.852 -3.526 1.8420

d.drug.psych -16.0583 -8.2343 -6.629 -5.006 2.8167

d.drug_AcuStim.psych -2.1451 5.7354 7.400 9.045 16.6498

d.drug_psych.psych 4.9265 12.8306 14.400 15.959 23.8012

sd.d 0.1006 0.9668 2.064 4.146 11.5244

-- Model fit (residual deviance):

Dbar pD DIC

18.72971 18.40965 37.13936

19 data points, ratio 0.9858, I^2 = 4%

# PSQI

## consistency

Results on the Mean Difference scale

Iterations = 20001:70000

Thinning interval = 1

Number of chains = 4

Sample size per chain = 50000

1. Empirical mean and standard deviation for each variable,

plus standard error of the mean:

Mean SD Naive SE Time-series SE

d.control.AcuStim -1.3278 0.9914 0.0022169 0.005111

d.control.drug -0.2391 1.2791 0.0028602 0.007237

d.drug.drug_AcuStim -2.1805 0.5380 0.0012030 0.001898

d.drug.drug_psych -2.9224 0.8997 0.0020117 0.002387

sd.d 1.1490 0.4137 0.0009251 0.002517

2. Quantiles for each variable:

2.5% 25% 50% 75% 97.5%

d.control.AcuStim -3.2862 -1.9586 -1.3287 -0.6985 0.6347

d.control.drug -2.7654 -1.0582 -0.2441 0.5725 2.3123

d.drug.drug_AcuStim -3.2762 -2.5060 -2.1727 -1.8478 -1.1254

d.drug.drug_psych -4.7191 -3.4615 -2.9216 -2.3825 -1.1183

sd.d 0.5879 0.8633 1.0690 1.3405 2.1887

-- Model fit (residual deviance):

Dbar pD DIC

22.67922 21.98863 44.66785

24 data points, ratio 0.945, I^2 = 0%

## ume

Results on the Mean Difference scale

Iterations = 20001:70000

Thinning interval = 1

Number of chains = 4

Sample size per chain = 50000

1. Empirical mean and standard deviation for each variable,

plus standard error of the mean:

Mean SD Naive SE Time-series SE

d.AcuStim.control 1.2655 1.1601 0.002594 0.005040

d.AcuStim.drug_AcuStim -1.0240 1.3795 0.003085 0.003505

d.control.drug -0.4219 1.9413 0.004341 0.012859

d.drug.drug_AcuStim -2.1987 0.5939 0.001328 0.001906

d.drug.drug_psych -2.9239 0.9802 0.002192 0.002494

sd.d 1.2426 0.4778 0.001068 0.003102

2. Quantiles for each variable:

2.5% 25% 50% 75% 97.5%

d.AcuStim.control -1.0268 0.5332 1.2691 1.9970 3.5617

d.AcuStim.drug_AcuStim -3.8016 -1.8339 -1.0241 -0.2110 1.7414

d.control.drug -4.2511 -1.6841 -0.4172 0.8372 3.3951

d.drug.drug_AcuStim -3.4127 -2.5492 -2.1885 -1.8389 -1.0345

d.drug.drug_psych -4.8886 -3.4998 -2.9236 -2.3474 -0.9575

sd.d 0.6109 0.9136 1.1432 1.4545 2.4698

-- Model fit (residual deviance):

Dbar pD DIC

23.08263 22.53863 45.62126

24 data points, ratio 0.9618, I^2 = 0.4%

# FSH

## consistency

Results on the Mean Difference scale

Iterations = 20001:70000

Thinning interval = 1

Number of chains = 4

Sample size per chain = 50000

1. Empirical mean and standard deviation for each variable,

plus standard error of the mean:

Mean SD Naive SE Time-series SE

d.drug.AcuStim -0.1216 1.6460 0.003681 0.007762

d.drug.control 0.8824 2.8200 0.006306 0.017555

d.drug.drug_AcuStim -5.7962 0.9704 0.002170 0.003742

d.drug.drug_psych -10.9000 3.1822 0.007116 0.009363

d.drug_psych.psych 10.4659 4.6009 0.010288 0.025590

sd.d 2.8174 0.7780 0.001740 0.005269

2. Quantiles for each variable:

2.5% 25% 50% 75% 97.5%

d.drug.AcuStim -3.401 -1.1862 -0.1166 0.9549 3.124

d.drug.control -4.698 -0.9835 0.8829 2.7570 6.400

d.drug.drug_AcuStim -7.765 -6.4103 -5.7814 -5.1693 -3.912

d.drug.drug_psych -17.236 -12.9190 -10.9048 -8.8794 -4.542

d.drug_psych.psych 1.387 7.4052 10.4704 13.5307 19.491

sd.d 1.593 2.2687 2.7174 3.2498 4.630

-- Model fit (residual deviance):

Dbar pD DIC

45.59406 39.72875 85.32281

48 data points, ratio 0.9499, I^2 = 0%

## ume

Results on the Mean Difference scale

Iterations = 20001:70000

Thinning interval = 1

Number of chains = 4

Sample size per chain = 50000

1. Empirical mean and standard deviation for each variable,

plus standard error of the mean:

Mean SD Naive SE Time-series SE

d.AcuStim.control 2.8555 3.4998 0.007826 0.017369

d.AcuStim.drug -0.9678 2.3784 0.005318 0.009587

d.AcuStim.drug_AcuStim -5.4029 2.5749 0.005758 0.008561

d.control.drug 1.9860 4.2931 0.009600 0.028516

d.drug.drug_AcuStim -5.8535 1.0715 0.002396 0.003805

d.drug.drug_psych -10.9229 3.3867 0.007573 0.009840

d.drug_psych.psych 10.4987 4.7612 0.010646 0.024943

sd.d 3.0182 0.8587 0.001920 0.005947

2. Quantiles for each variable:

2.5% 25% 50% 75% 97.5%

d.AcuStim.control -3.979 0.5265 2.8348 5.1529 9.8308

d.AcuStim.drug -5.699 -2.5049 -0.9541 0.5828 3.6878

d.AcuStim.drug_AcuStim -10.581 -7.0354 -5.3893 -3.7497 -0.3336

d.control.drug -6.337 -0.9068 1.9654 4.8362 10.5091

d.drug.drug_AcuStim -8.002 -6.5326 -5.8402 -5.1619 -3.7522

d.drug.drug_psych -17.672 -13.0557 -10.9135 -8.8086 -4.1677

d.drug_psych.psych 1.106 7.3500 10.4975 13.6492 19.8936

sd.d 1.695 2.4152 2.8972 3.4856 5.0306

-- Model fit (residual deviance):

Dbar pD DIC

45.32315 40.96048 86.28363

48 data points, ratio 0.9442, I^2 = 0%

# LH

## consistency

Results on the Mean Difference scale

Iterations = 20001:70000

Thinning interval = 1

Number of chains = 4

Sample size per chain = 50000

1. Empirical mean and standard deviation for each variable,

plus standard error of the mean:

Mean SD Naive SE Time-series SE

d.AcuStim.control -0.54341 3.182 0.007115 0.011742

d.AcuStim.drug -0.08635 2.213 0.004949 0.012746

d.AcuStim.drug_AcuStim -6.81679 2.369 0.005296 0.013647

sd.d 5.10720 1.209 0.002703 0.007443

2. Quantiles for each variable:

2.5% 25% 50% 75% 97.5%

d.AcuStim.control -6.944 -2.577 -0.51083 1.529 5.664

d.AcuStim.drug -4.503 -1.505 -0.08884 1.332 4.324

d.AcuStim.drug_AcuStim -11.632 -8.305 -6.78484 -5.279 -2.216

sd.d 3.158 4.254 4.96815 5.805 7.859

-- Model fit (residual deviance):

Dbar pD DIC

56.4188 47.2692 103.6880

50 data points, ratio 1.128, I^2 = 13%

## ume

Results on the Mean Difference scale

Iterations = 20001:70000

Thinning interval = 1

Number of chains = 4

Sample size per chain = 50000

1. Empirical mean and standard deviation for each variable,

plus standard error of the mean:

Mean SD Naive SE Time-series SE

d.AcuStim.control -1.0165 4.364 0.009757 0.012297

d.AcuStim.drug -1.3257 3.103 0.006939 0.008765

d.AcuStim.drug_AcuStim -4.3871 4.099 0.009166 0.010081

d.control.drug 0.0196 4.676 0.010457 0.016323

d.drug.drug_AcuStim -7.1343 1.534 0.003430 0.004333

sd.d 5.4698 1.328 0.002970 0.008219

2. Quantiles for each variable:

2.5% 25% 50% 75% 97.5%

d.AcuStim.control -9.679 -3.815 -1.0153 1.7700 7.655

d.AcuStim.drug -7.610 -3.292 -1.2914 0.6896 4.728

d.AcuStim.drug_AcuStim -12.620 -6.966 -4.3670 -1.7868 3.745

d.control.drug -9.039 -3.046 -0.0694 3.0036 9.530

d.drug.drug_AcuStim -10.314 -8.089 -7.0917 -6.1326 -4.206

sd.d 3.313 4.531 5.3193 6.2361 8.491

-- Model fit (residual deviance):

Dbar pD DIC

55.50857 47.88530 103.39387

50 data points, ratio 1.11, I^2 = 12%

# E2

## consistency

Results on the Mean Difference scale

Iterations = 20001:70000

Thinning interval = 1

Number of chains = 4

Sample size per chain = 50000

1. Empirical mean and standard deviation for each variable,

plus standard error of the mean:

Mean SD Naive SE Time-series SE

d.drug.AcuStim 5.279 3.220 0.007200 0.010913

d.drug.control -2.045 5.055 0.011303 0.021378

d.drug.drug_AcuStim 5.125 2.062 0.004611 0.006011

d.drug.drug_psych 10.672 7.838 0.017527 0.017665

d.drug_psych.psych -17.058 5.845 0.013071 0.016117

sd.d 7.652 1.541 0.003447 0.008275

2. Quantiles for each variable:

2.5% 25% 50% 75% 97.5%

d.drug.AcuStim -1.039 3.172 5.255 7.365 11.725

d.drug.control -12.187 -5.317 -1.988 1.301 7.759

d.drug.drug_AcuStim 1.111 3.772 5.108 6.453 9.271

d.drug.drug_psych -4.927 5.644 10.644 15.712 26.242

d.drug_psych.psych -28.508 -20.892 -17.100 -13.307 -5.308

sd.d 5.164 6.566 7.470 8.538 11.185

-- Model fit (residual deviance):

Dbar pD DIC

58.38360 52.55811 110.94171

57 data points, ratio 1.024, I^2 = 4%

## ume

Results on the Mean Difference scale

Iterations = 20001:70000

Thinning interval = 1

Number of chains = 4

Sample size per chain = 50000

1. Empirical mean and standard deviation for each variable,

plus standard error of the mean:

Mean SD Naive SE Time-series SE

d.AcuStim.control -1.978 5.590 0.012499 0.015814

d.AcuStim.drug -10.230 3.959 0.008853 0.012097

d.AcuStim.drug_AcuStim 2.416 4.332 0.009688 0.011445

d.control.drug 10.599 7.170 0.016032 0.037537

d.drug.drug_AcuStim 4.288 2.099 0.004694 0.005763

d.drug.drug_psych 10.641 7.327 0.016383 0.016447

d.drug_psych.psych -17.184 5.484 0.012263 0.015550

sd.d 7.132 1.535 0.003432 0.008843

2. Quantiles for each variable:

2.5% 25% 50% 75% 97.5%

d.AcuStim.control -13.0406 -5.5875 -1.999 1.630 9.125

d.AcuStim.drug -18.1813 -12.7923 -10.197 -7.647 -2.465

d.AcuStim.drug_AcuStim -6.2692 -0.3412 2.436 5.224 10.940

d.control.drug -3.3652 5.8468 10.546 15.269 24.965

d.drug.drug_AcuStim 0.2282 2.9172 4.247 5.618 8.567

d.drug.drug_psych -3.9926 5.9849 10.634 15.332 25.183

d.drug_psych.psych -27.8920 -20.7623 -17.236 -13.703 -6.067

sd.d 4.6486 6.0495 6.954 8.014 10.615

-- Model fit (residual deviance):

Dbar pD DIC

58.08460 52.64429 110.72889

57 data points, ratio 1.019, I^2 = 4%
