# Supplementary material for: Pharmacotherapy, acupoint stimulation, and psychotherapy for perimenopausal women with anxiety, depression, and panic disorder: a systematic review and network meta-analysis of randomized controlled trials
Source: Front Psychiatry. 2026 Jul 17;17:1845876. doi: 10.3389/fpsyt.2026.1845876 (PMC13423873; doi:10.3389/fpsyt.2026.1845876)
Supplement: Supplementary file 1 [file Supplementaryfile1.zip › Manuscript_Supplementary_Figure_Table/Supplementary Material 5-loop inconsitstency results.docx]

## HAMD-ALL

Treatments used

A (reference): AcuStim

B: AcuStim_psych

C: control

D: drug

E: drug_AcuStim

F: drug_psych

## HAMD-drug

Treatments used

A (reference): ADs

B: ADs_AP

C: ADs_HRT

D: ADs_TCM

E: HRT

F: SNRI

G: SSRI

H: TCA

I: TCM

J: control

K: delexin

## HAMA-ALL

Treatments used

A (reference): AcuStim

B: AcuStim_psych

C: control

D: drug

E: drug_AcuStim

F: drug_psych

## HAMA-drug

Treatments used

A (reference): ADs_AP

B: ADs_HRT

C: BZD

D: HRT

E: SNRI

F: SSRI

G: TCA

H: TCM

I: control

## clinical efficacy-all

Treatments used

A (reference): AcuStim

B: control

C: drug

D: drug_AcuStim

E: drug_psych

F: psych

## clinical efficacy-drug

Treatments used

A (reference): ADs_AP

B: ADs_HRT

C: ADs_HRT_TCM

D: ADs_TCM

E: HRT

F: SSRI

G: TCA

H: delexin

I: oryzanol

## AE-ALL

Treatments used

A (reference): AcuStim

B: control

C: drug

D: drug_AcuStim

## AE-drug

Treatments used

A (reference): ADs_HRT

B: ADs_TCM

C: SNRI

D: SSRI

E: TCA

F: control

G: delexin

## KI

Treatments used

A (reference): AcuStim

B: control

C: drug

D: drug_AcuStim

E: drug_psych

## SDS

Treatments used

A (reference): AcuStim

B: control

C: drug

D: drug_AcuStim

E: drug_psych

F: psych

## SAS

Treatments used

A (reference): AcuStim

B: control

C: drug

D: drug_AcuStim

E: drug_psych

F: psych

## PSQI

Treatments used

A (reference): AcuStim

B: control

C: drug

D: drug_AcuStim

E: drug_psych

## FSH

Treatments used

A (reference): AcuStim

B: control

C: drug

D: drug_AcuStim

E: drug_psych

F: psych

## LH

Treatments used

A (reference): AcuStim

B: control

C: drug

D: drug_AcuStim

## E2

Treatments used

A (reference): AcuStim

B: control

C: drug

D: drug_AcuStim

E: drug_psych

F: psych
