# Supplementary material for: Pharmacotherapy, acupoint stimulation, and psychotherapy for perimenopausal women with anxiety, depression, and panic disorder: a systematic review and network meta-analysis of randomized controlled trials
Source: Front Psychiatry. 2026 Jul 17;17:1845876. doi: 10.3389/fpsyt.2026.1845876 (PMC13423873; doi:10.3389/fpsyt.2026.1845876)
Supplement: Supplementary file 1 [file Supplementaryfile1.zip › Manuscript_Supplementary_Figure_Table/Supplementary Material 7-forest plots.docx]

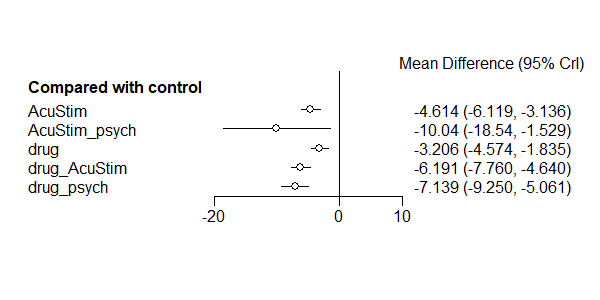
Figure S2. 1 The forest plot for HAMD within the overall network of the three major intervention categories (drug, Acupoint Stimulation, and psychotherapy).


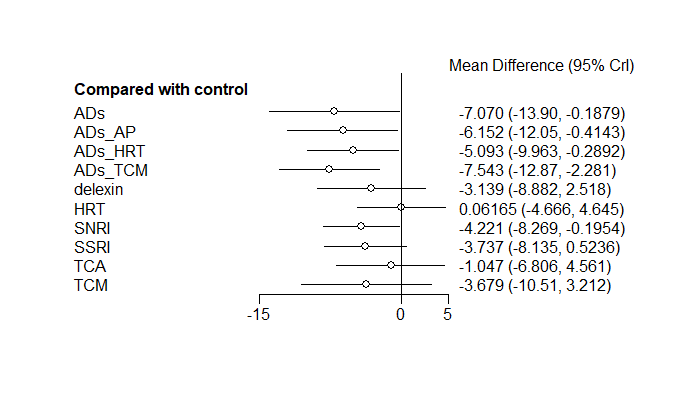


Figure S2. 2 The forest plot for HAMD within the pharmacotherapy subgroups.


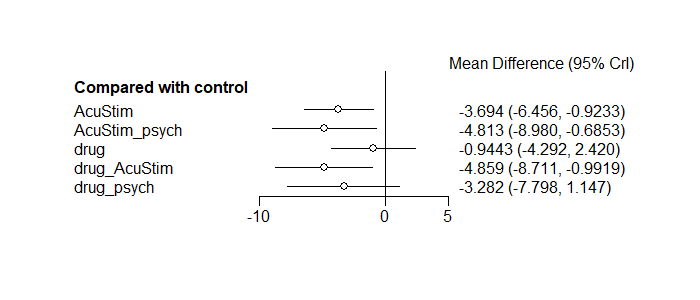


Figure S2. 3 The forest plot for HAMA within the overall network of the three major intervention categories (drug, Acupoint Stimulation, and psychotherapy).


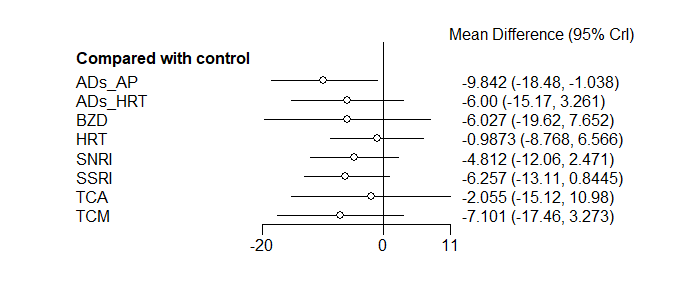
Figure S2. 4 The forest plot for HAMA within the pharmacotherapy subgroups.


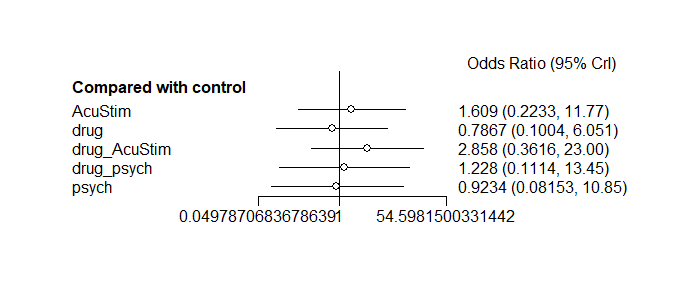


Figure S2. 5 The forest plot for clinical efficacy within the overall network of the three major intervention categories (drug, Acupoint Stimulation, and psychotherapy).


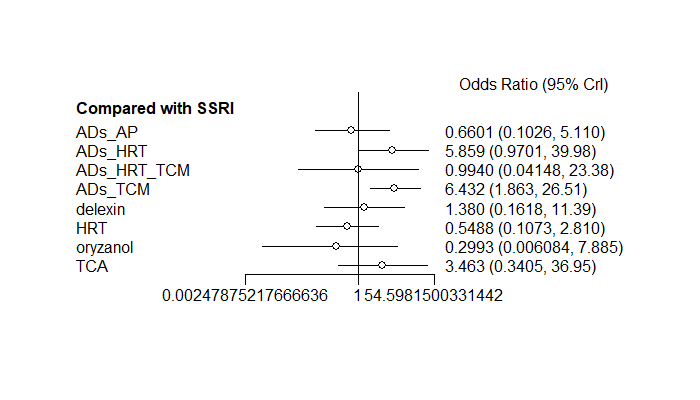
Figure S2. 6 The forest plot for clinical efficacy within the pharmacotherapy subgroups.


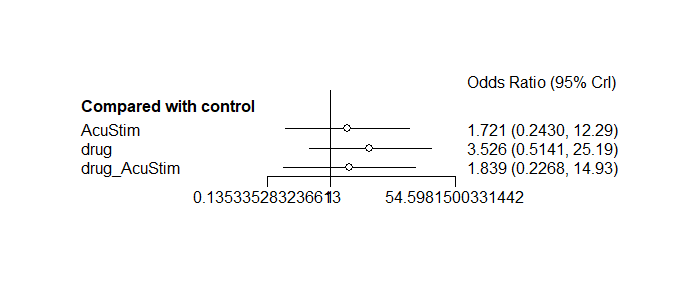


Figure S2. 7 The forest plot for AE within the overall network of the three major intervention categories (drug, Acupoint Stimulation, and psychotherapy).


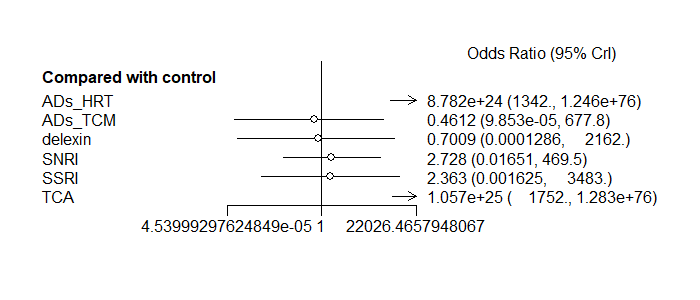


Figure S2. 8 The forest plot for clinical efficacy within the pharmacotherapy subgroups.


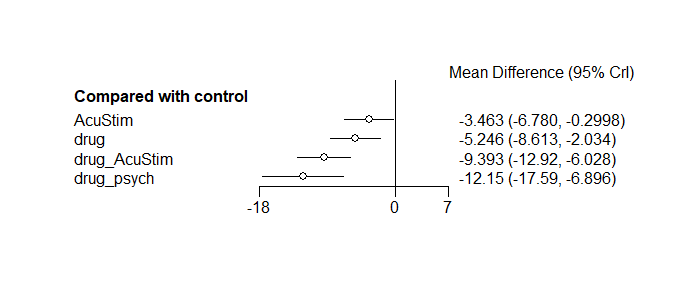


Figure S2. 9 The forest plot for KI within the overall network of the three major intervention categories (drug, Acupoint Stimulation, and psychotherapy).


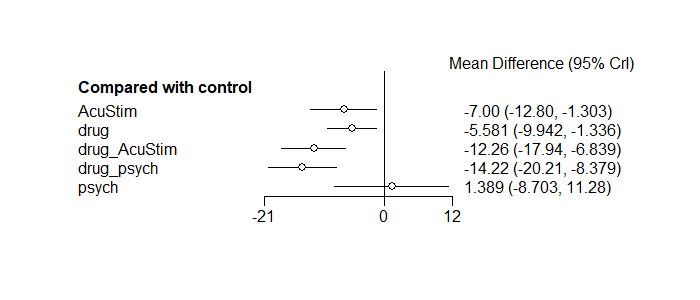


Figure S2. 10 The forest plot for SDS within the overall network of the three major intervention categories (drug, Acupoint Stimulation, and psychotherapy).


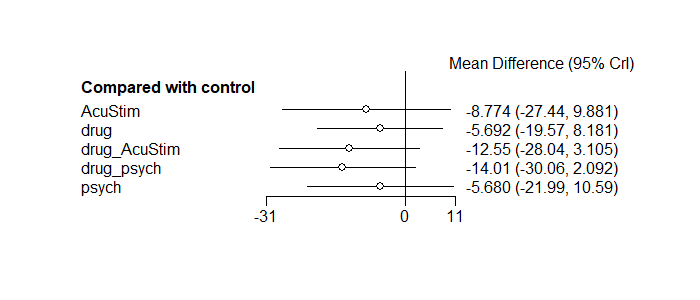


Figure S2. 11 The forest plot for SAS within the overall network of the three major intervention categories (drug, Acupoint Stimulation, and psychotherapy).


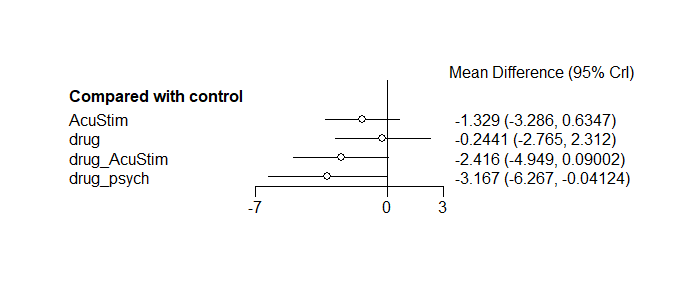


Figure S2. 12 The forest plot for PSQI within the overall network of the three major intervention categories (drug, Acupoint Stimulation, and psychotherapy).


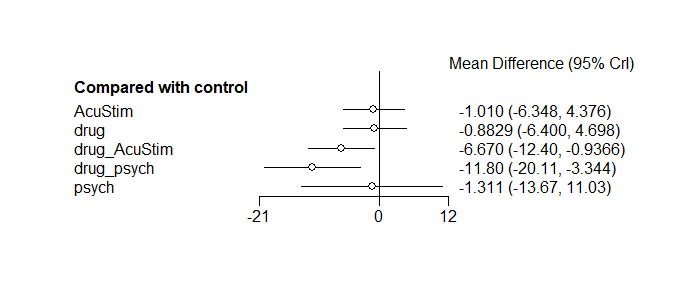


Figure S2. 13 The forest plot for FSH within the overall network of the three major intervention categories (drug, Acupoint Stimulation, and psychotherapy).


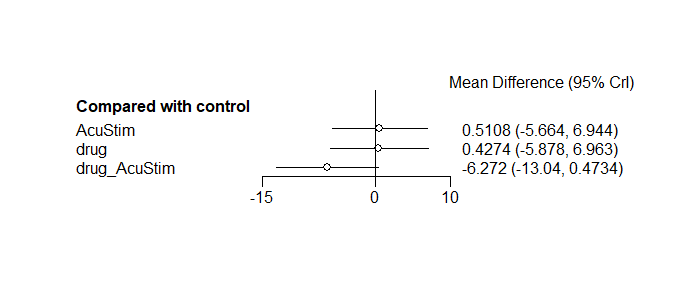


Figure S2. 14 The forest plot for LH within the overall network of the three major intervention categories (drug, Acupoint Stimulation, and psychotherapy).


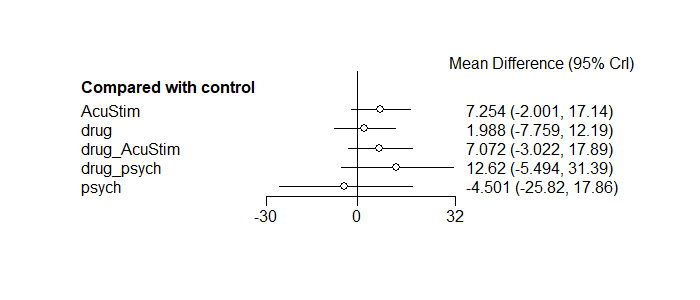


Figure S2. 15 The forest plot for E2 within the overall network of the three major intervention categories (drug, Acupoint Stimulation, and psychotherapy).
