# Supplementary material for: Pharmacotherapy, acupoint stimulation, and psychotherapy for perimenopausal women with anxiety, depression, and panic disorder: a systematic review and network meta-analysis of randomized controlled trials
Source: Front Psychiatry. 2026 Jul 17;17:1845876. doi: 10.3389/fpsyt.2026.1845876 (PMC13423873; doi:10.3389/fpsyt.2026.1845876)
Supplement: Supplementary file 1 [file Supplementaryfile1.zip › Manuscript_Supplementary_Figure_Table/Supplementary Material 8-subgroup analysis.docx]

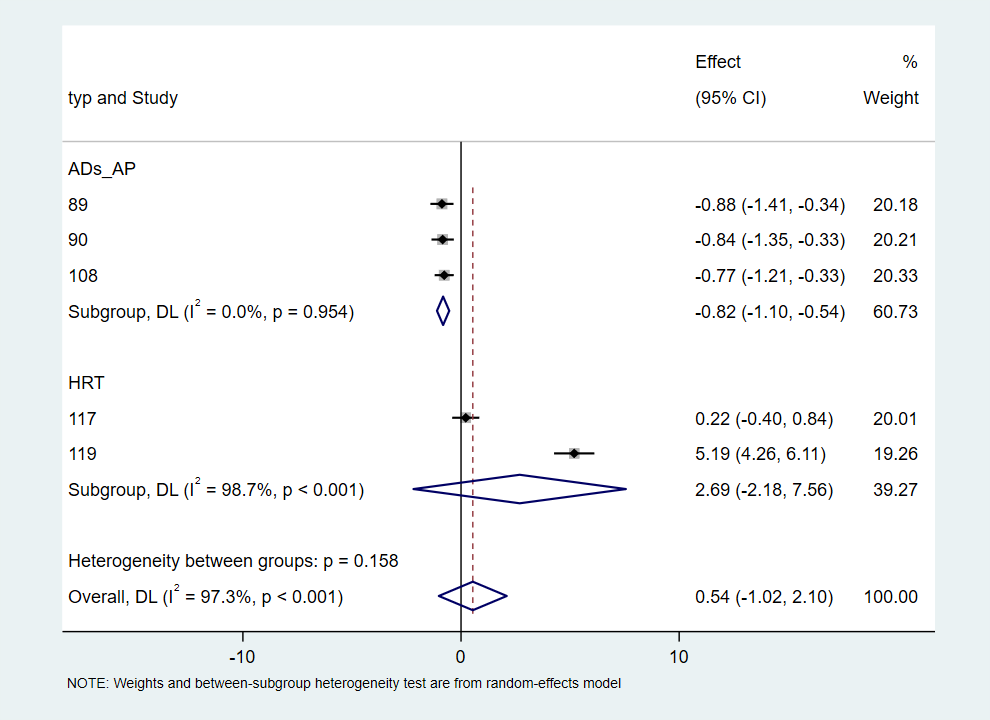


Figure S3 1 Subgroup analysis forest plot for HAMA, stratified by pharmacotherapy type, with SSRI as the reference.


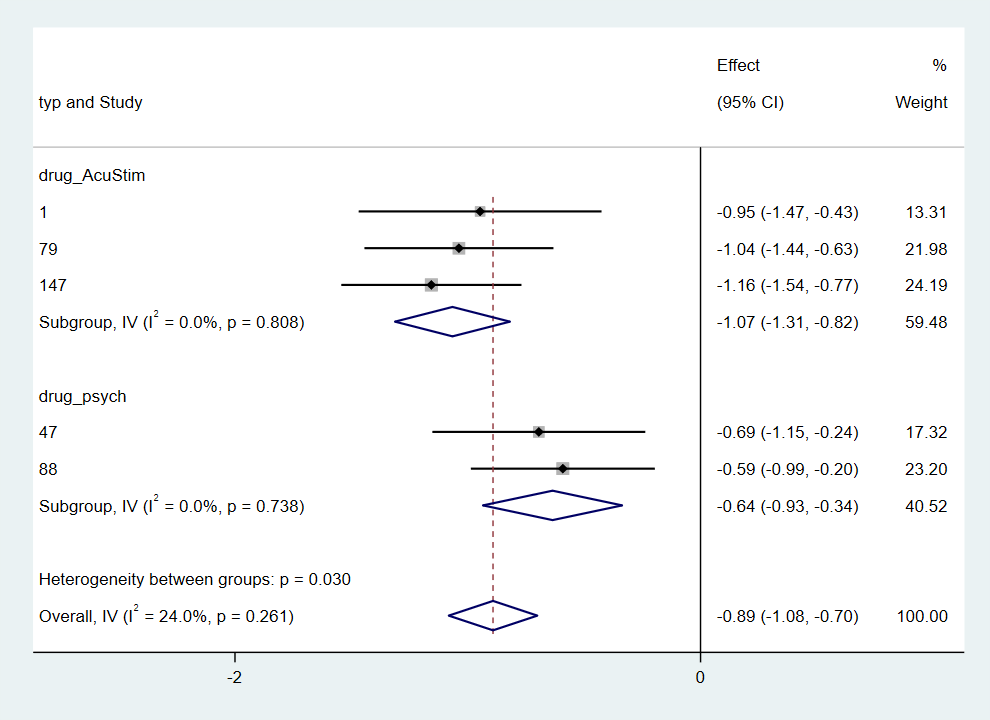


Figure S3 2 Subgroup analysis forest plot for SAS, stratified by intervention type, with drug as the reference.
